# Circulating human microRNAs are not linked to JC polyomavirus serology or urinary viral load in healthy subjects

**DOI:** 10.1186/1743-422X-11-41

**Published:** 2014-03-03

**Authors:** Ole Lagatie, Tom Van Loy, Luc Tritsmans, Lieven J Stuyver

**Affiliations:** 1 Janssen Diagnostics, Turnhoutseweg 30, 2340 Beerse, Belgium; 2 Janssen Research and Development, Turnhoutseweg 30, 2340 Beerse, Belgium

**Keywords:** JC polyomavirus, microRNA, Circulating microRNA, Plasma, Serology, Viral load

## Abstract

**Background:**

JC polyomavirus (JCPyV) is a widespread human polyomavirus that usually resides latently in its host. It can be reactivated under immunomodulating conditions and cause Progressive Multifocal Leukoencephalopathy (PML). Circulating microRNAs (miRNAs) are emerging as promising biomarkers for several pathologies. In this study, we have investigated whether circulating miRNAs exist that are differentially expressed between JCPyV seropositive and JCPyV seronegative on the one hand or between JCPyV shedders and JCPyV non-shedders on the other hand.

**Methods:**

Human miRNA expression profiling was performed in a small set of plasma samples obtained from seronegative subjects, seropositive shedders and seropositive non-shedders. A set of 10 miRNAs was selected for further analysis in a larger group of samples.

**Results:**

Based on the plasma profiling experiment of 30 samples, 6 miRNAs were selected that were possibly differentially expressed between seropositive and seronegative subjects and 4 miRNAs were selected that were possibly differentially expressed between shedders and non-shedders. Subsequently, expression of these 10 selected miRNAs was assessed in an independent set of 100 plasma samples. Results indicated that none of them were differentially expressed.

**Conclusion:**

This study could not identify circulating human miRNAs that were differentially expressed between plasma from JCPyV seropositive and JCPyV seronegative subjects or between JCPyV shedders and JCPyV non-shedders.

## Background

Progressive Multifocal Leukoencephalopathy (PML) is a demyelinating disease of the brain caused by reactivation of the human JC polyomavirus (JCPyV) resulting in lytic infection of oligodendrocytes [1]. JCPyV is a circular double-stranded DNA virus with very restricted cellular tropism, infecting oligodendrocytes, astrocytes, kidney epithelial cells and peripheral blood cells [2]. It is thought that infection usually occurs asymptomatically in childhood, after which the virus remains latent in the body [3-5]. Under certain immunocompromised conditions, such as treatment with immunomodulatory drugs or infection with Human Immunodeficiency Virus (HIV), the virus can be reactivated and actively replicate into the brain, leading to PML. Current diagnostic tools to assess risk for developing PML consist of the detection of antibodies against VP1, the major capsid protein and the detection of viral DNA in urine. It has been reported that 50 to 60% of humans are seropositive for JCPyV [6,7]. Approximately one fifth of the population sheds JCPyV in the urine [8,9]. The prognostic value of both tests (viruria and antibody titer) is being debated [10,11]. Also, healthy seropositive subjects or subjects who shed virus in the urine are not at risk of developing PML, indicating the limited prognostic value of these two tests. Recent work has also shown that most healthy subjects have antibodies against peptides derived from the JCPyV proteome, suggesting that current serology tests might underestimate the real infection rate [12]. JCPyV is very rarely detected in peripheral blood in immunocompetent people but can sometimes be detected in patients treated with immunomodulatory therapies such as natalizumab [13]. However, the detection of JCPyV in serum has not been correlated with progression to PML [14]. Development of new tools for stratification of those patients at risk for developing PML is therefore of great interest.

MicroRNAs (miRNAs) are small, non-coding RNAs that play an important role in fine-tuning the expression of specific gene products through translational repression and/or mRNA degradation [15]. Due to the role they play in many cellular processes, they are implicated in many diseases [16,17]. Cellular miRNAs can also be released in small vesicles and the levels of these extracellular miRNAs in biological fluids have become very valuable markers of several diseases, such as cancer, Alzheimer’s disease and diabetes [18-20]. The possible use of circulating miRNAs as diagnostic marker for viral infection has also been tested for viruses like influenza A virus, hepatitis B virus, hepatitis C virus and Epstein-Barr [21-24].

Whether circulating human miRNAs play a role in JCPyV related pathology or whether different plasma miRNA expression patterns can be observed in JCPyV infected or uninfected subjects, is not known [25]. In this study we have therefore investigated the plasma miRNA profile of healthy subjects and assessed whether certain miRNAs could be identified that were linked to JCPyV serostatus or urinary viral load.

## Methods

### Ethics statement

The Ethics Committee [“Commissie voor Medische Ethiek - ZiekenhuisNetwerk Antwerpen (ZNA) and the Ethics committee University Hospital Antwerp] approved the Protocols, and Informed consents, which were signed by all subjects.

### Healthy subject samples

A total of 254 healthy subjects (HSs) were selected in Belgium for this study: 135 women and 119 men with an age ranging from 19 up to 66 years (median 42 y, average 42.2 y, 25% percentile 35 y, 75% percentile 50 y). Plasma samples and urine samples were collected from all these HSs and stored at −80°C until further processing.

### JC polyomavirus viral load assay

Analysis of the urinary viral load was performed as described previously [9].

### JC polyomavirus VP1 serology assay

The anti- JCPyV antibody assay was performed similarly to the method described earlier [26]. The assay utilized *Saccharomyces cerevisiae* expressed JCPyV VP1 capsid protein (Abcam, UK). In the ELISA, JCPyV VP1 was immobilized onto a microtiter plate and blocked to minimize nonspecific binding. Diluted (1:200) plasma samples were added to the plate and incubated. The plate was then washed, and mouse monoclonal anti-human immunoglobulin G (IgG) (OCD) conjugated with horseradish peroxidase (HRP) was added. After a wash step, substrate solution was added and incubated. The reaction was stopped with acid solution before measuring the optical density (OD) values at 450 nm using a plate reader. Samples were considered positive if OD values were higher than OD value + 3 SD of the negative control plasma sample.

### miRNA expression profiling

miRNA expression profiling was performed by Biogazelle (Gent, Belgium) using a validated miRNA screening pipeline that allows for accurate and sensitive expression analysis of 755 human microRNAs by means of quantitative reverse transcriptase PCR (qRT-PCR) with hydrolysis probe based miRNA assays [27]. Briefly, RNA was isolated from 200 μL plasma using the miRNeasy kit (Qiagen) according to the manufacturer’s instructions. Six μL of total RNA (representing 40% of total RNA extract) was reverse transcribed using the Megaplex RT stem-loop primer pool (Life Technologies), enabling miRNA specific cDNA synthesis of 755 different human miRNAs and small RNA controls. Subsequently, the Megaplex RT product was pre-amplified by means of a 12-cycle PCR reaction with a miRNA specific forward primer and universal reverse primer to increase detection sensitivity. Diluted pre-amplified miRNA cDNA was then used as input for a 40-cycle qPCR reaction with miRNA specific hydrolysis probes and primers (Life Technologies). All reactions were performed in Taqman arrays on the ViiA7 instrument (Life Technologies) using the gene maximization strategy. Quantification cycle (Cq) values were filtered using a detection cut-off of 32 cycles. Cq values above 32 were considered noise. Data normalization was done in qbase + following the modified global mean normalization procedure (common targets), as previously described [28]. Note that, while Cq-values are log_2_-based, normalized expression data is log_10_-based.

### Expression analysis of selected miRNAs

Expression of selected miRNAs was determined similarly as described above, except that 3 μL of total RNA was used for reverse transcription. Three stable miRNAs (hsa-miR-26a, has-miR-30b and mmu-miR-93) were included for normalization. Note that, while Cq-values are log_2_-based, normalized expression data is log_10_-based.

### Statistical analysis

The differences between groups were assessed using a Mann-Whitney test. For analysis of miRNA profiling data, Mann-Whitney *p*-values were corrected for multiple testing using the Benjamini-Hochberg procedure [29]. Differences were considered statistically significant at *p* < 0.05. For selection of miRNAs from the profiling study for subsequent confirmation, miRNAs were first selected based on log_2_ (fold change) > 0.8 or log_2_ (fold change) < -0.8 and were then ranked based on *p*-value. The 6 miRNAs with the lowest *p*-value for comparison of seronegative subjects vs. seropositive subjects and the 4 miRNAs with the lowest *p*-value for comparison of shedders vs. non-shedders were selected.

## Results

### Subject classification

Urine samples and plasma samples were donated by 254 HSs. All plasma samples were screened for the presence of anti- JCPyV VP1 antibodies by ELISA. 193 out of 254 HSs (~76.0%) had anti- JCPyV antibodies in their plasma. All urine samples were screened for the presence of JCPyV DNA by quantitative PCR. Within the group of seronegative subjects, none of the HSs had shed viral DNA into their urine. Within the group of seropositive subjects, 63 (~24.8%) had shed viral DNA into their urine. Based on these results, subjects were classified as seronegative (Ab^−^), seropositive shedder (Ab^+^ VL^+^) or seropositive non-shedder (Ab^+^ VL^−^). Selection of subjects for miRNA profiling or subsequent validation studies was done based on these classifications. Characteristics of subjects selected for the miRNA profiling and for the miRNA validation/confirmation are shown in Tables 1 and 2, respectively.

**Table 1:** Characteristics of healthy subjects in the miRNA profiling study

		**Median age [range] or number of subjects (%)**			
		**Ab**^**−**^	**Ab**^**+ **^**VL**^**+**^	**Ab**^**+ **^**VL**^**−**^	
		**n = 10**	**n = 10**	**n = 10**	***p*-value**^**1**^
Age		40 [27-55]	49 [38-59]	37 [26-54]	0.468/0.012*
Sex	Male	3 (30.0)	5 (50.0)	5 (50.0)	
	Female	7 (70.0)	5 (50.0)	5 (50.0)	

^1^Ab^−^ vs. Ab^+^ / Ab^+^VL^+^ vs. Ab^+^VL^−^.

*significant difference (*p* < 0.05).

**Table 2:** Characteristics of healthy subjects in the miRNA validation/confirmation study

		**Median age [range] or number of subjects (%)**			
		**Ab**^**−**^	**Ab**^**+ **^**VL**^**+**^	**Ab**^**+ **^**VL**^**−**^	
		**n = 35**	**n = 25**	**n = 40**	***p*-value**^**1**^
Age		44 [21-65]	51 [24-64]	47 [20-66]	0.115/0.830
Sex	Male	16 (45.7)	12 (48.0)	16 (40.0)	
	Female	19 (54.3)	13 (52.0)	24 (60.0)	

^1^Ab^−^ vs. Ab^+^ / Ab^+^VL^+^ vs. Ab^+^VL^−^.

### Plasma miRNA profiling

**Figure 1:**
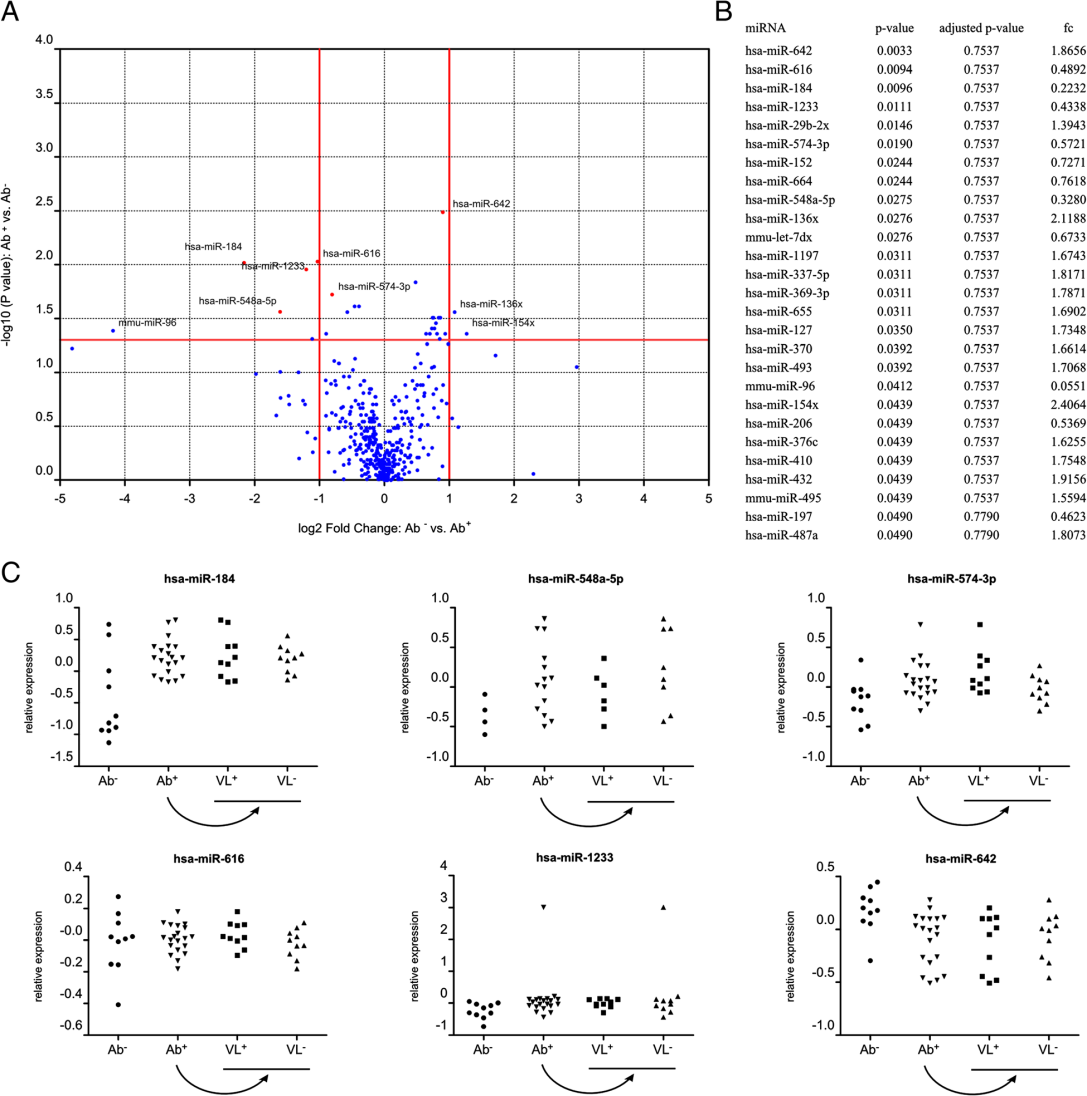
**Comparison of all miRNAs assessed by RT-qPCR analysis of RNA isolated from plasma of JCPyV seronegative subjects (Ab**^**−**^**, n = 10) or JCPyV seropositive subjects (Ab**^**+**^**, n = 20). A**. Volcano plot of pairwise comparisons. The volcano plot displays the relationship between fold-change and significance between the two groups, using a scatter plot view. The y-axis is the negative log10 of P values (a higher value indicates greater significance) and the x-axis is the difference in expression between two experimental groups as measured in log2 space. MiRNAs that were selected for further analysis are indicated in red on the plot. **B**. Mann-Whitney p-values, adjusted p-values and fold changes (fc) of JCPyV seronegative subjects (Ab^−^) vs. JCPyV seropositive subjects (Ab^+^) for miRNAs with p-value < 0.05. **C**. Relative expression levels of selected circulating miRNAs in subjects from each group. Data is presented relative to the mean of the entire population in log10 scale (n = 30).

**Figure 2:**
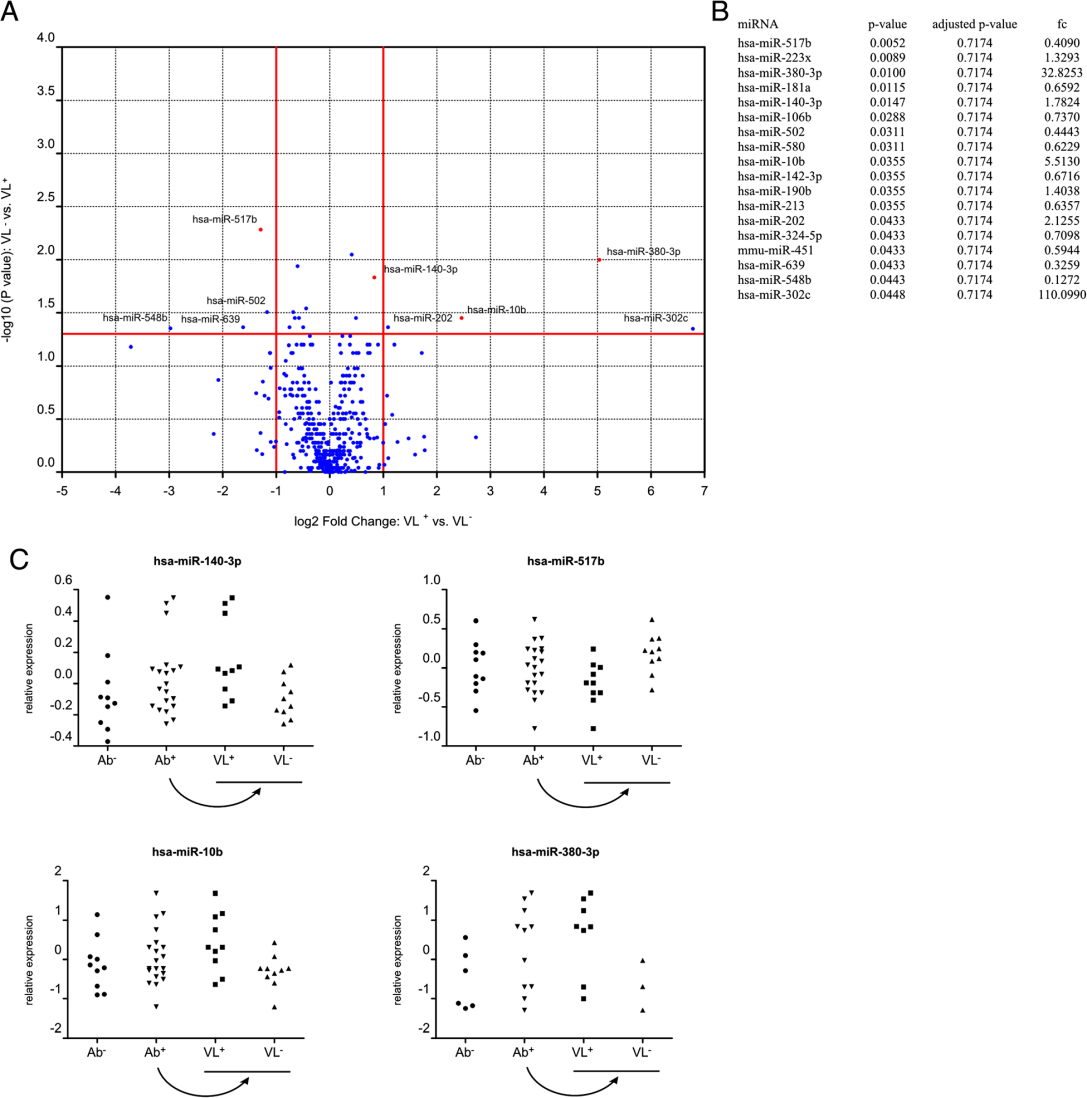
**Comparison of all miRNAs assessed by RT-qPCR analysis of RNA isolated from plasma of subjects with negative urine JCPyV Viral Load (VL**^**−**^**, n = 10) or subjects with positive urine JCPyV Viral Load (VL**^**+**^**, n = 10). A**. Volcano plot of pairwise comparisons. The volcano plot displays the relationship between fold-change and significance between the two groups, using a scatter plot view. The y-axis is the negative log10 of P values (a higher value indicates greater significance) and the x-axis is the difference in expression between two experimental groups as measured in log2 space. MiRNAs that were selected for further analysis are indicated in red on the plot. **B**. Mann-Whitney p-values, adjusted p-values and fold changes (fc) of subjects with positive urine JCPyV Viral Load (VL^+^) vs. subjects with negative urine JCPyV Viral Load (VL^−^) for miRNAs with *p*-value < 0.05. **C**. Relative expression levels of selected circulating miRNAs in subjects from each group. Data is presented relative to the mean of the entire population in log10 scale (n = 30).

We initially performed expression profiling of 755 miRNAs in the plasma samples of 30 HSs: 10 Ab^−^, 10 Ab^+^ VL^+^ and 10 Ab^+^ VL^−^. A list with all miRNA assays used, including the miRBase ID and sequence of the corresponding hsa-miR, is provided (Additional file 1: Table S1), as well as a table with all raw Cq values (Additional file 2: Table S2). Volcano plots were constructed to provide information about the significance and magnitude of expressive alteration of these miRNAs between 10 seronegative subjects and 20 seropositive subjects on the one hand (Figure 1) and 10 shedders and 10 non-shedders on the other hand (Figure 2). 27 miRNAs were found to be differentially expressed between seronegative subjects and seropositive subjects (*p* < 0.05), of which 2 had a fold change > 2 and 4 had a fold change < 0.5. 18 miRNAs were found to be differentially expressed between shedders and non-shedders (*p* < 0.05), of which 4 had a fold change > 2 and 4 had a fold change < 0.5. However, upon correction for multiple testing using the Benjamini-Hochberg multiple testing correction procedure, none of these remained significant. This might be due to the relatively small sample size, but might also be indicative of true absence of differentially expressed miRNAs between groups.

### Validation/confirmation study

**Figure 3:**
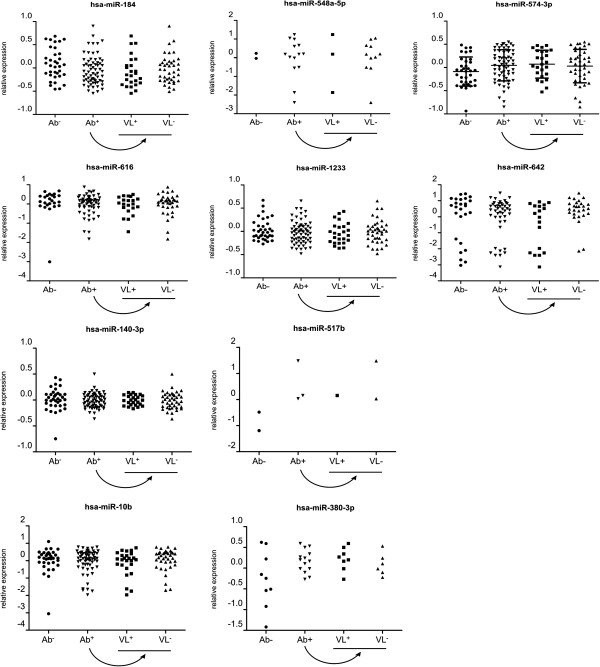
**Relative expression levels of selected circulating miRNAs in subjects from each group in the validation study (Ab**^**−**^**, Ab**^**+**^**, VL**^**−**^**, VL**^**+**^**).** Data is presented relative to the mean of the entire population in log10 scale (n = 100). * *p*-value < 0.05.

Next, we performed qRT-PCR on RNA isolated from plasma of another 100 HSs: 35 Ab^−^, 25 Ab^+^ VL^+^ and 40 Ab^+^ VL^−^. We evaluated the levels of miRNAs that, based on our profiling study, tended to have different levels of expression between two groups. This analysis included miR-184, miR-548a-5p, miR-574-3p, miR-616, miR-1233, miR-642, miR-140-3p, miR-517b, miR-10b and miR-380-3p (Figure 3, Tables 3 and 4 and Additional file 3: Table S3). We found that of these 10 selected miRNAs only miR-548a-5p and miR-574-3p were differentially expressed between seronegative subjects (Ab^−^) and seropositive subjects (Ab^+^) with statistical significance (*p* < 0.05). However, careful examination of the data learns that for miR-548a-5p the number of subjects with detectable levels of this miRNA is too low for proper statistical interpretation. For miR-574-3p the difference between both groups is statistically significant (*p* = 0.028) but since the fold change is only 1.36 this difference is unlikely to be biologically significant. Between shedders (VL^+^) and non-shedders (VL^−^) none of the miRNAs were found to be differentially expressed. Also, signal intensities for miR-548a-5p, miR-517b and miR-380-3p were very low and not detected in most samples. For comprehensiveness, it should also be noted that analysis of the data for gender-driven differences showed that there is a significantly higher expression of hsa-miR-10b and hsa-miR-642 in males compared to females. However, re-analysis of the data using gender as a co-variable did not show any significant difference between seropositive and seronegative subjects, nor between shedders and non-shedders (results not shown).

**Table 3:** **Number of subjects in the validation study with detectable levels of selected circulating miRNAs, Mann-Whitney *p*-values and fold changes (fc) of Ab**^**− **^**vs. Ab**^**+ **^**subjects**

**miRNA**	**Ab**^**− **^**(n = 35)**	**Ab**^**+ **^**(n = 65)**	***p*-value**	**fc**
Hsa-miR-10b	33	62	0.9856	−0.053
Hsa-miR-140-3p	35	65	0.3456	−0.084
Hsa-miR-380-3p	9	14	0.8293	0.103
Hsa-miR-517b	2	3	0.8633	0.190
Hsa-miR-1233	35	65	0.3779	−0.179
Hsa-miR-184	35	65	0.0697	−0.415
Hsa-miR-548a-5p	2	14	0.0410	1.393
Hsa-miR-574-3p	35	65	0.0280	0.447
Hsa-miR-616	24	56	0.6294	1.678
Hsa-miR-642	26	45	0.3721	−0.458

**Table 4:** **Number of subjects in the validation study with detectable levels of selected circulating miRNAs, Mann-Whitney *p*-values and fold changes (fc) of VL**^**− **^**vs. VL**^**+ **^**subjects**

**miRNA**	**VL**^**− **^**(n = 40)**	**VL**^**+ **^**(n = 25)**	***p*-value**	**fc**
Hsa-miR-10b	37	25	0.576	−0.196
Hsa-miR-140-3p	40	25	0.736	−0.019
Hsa-miR-380-3p	6	8	0.083	−1.164
Hsa-miR-517b	2	1	0.867	0.155
Hsa-miR-1233	40	25	0.504	0.108
Hsa-miR-184	40	25	0.325	0.190
Hsa-miR-548a-5p	11	3	0.157	1.470
Hsa-miR-574-3p	40	25	0.793	−0.139
Hsa-miR-616	35	21	0.438	0.607
Hsa-miR-642	27	18	0.396	1.635

## Discussion

Several studies indicate that there is a potential role for circulating miRNA levels as valuable biomarkers for certain physiological events or disease. It has been shown in different studies that a large portion of the human population has been exposed to JCPyV as they carry antibodies against the JCPyV VP1 capsid protein [30,31]. This might raise the question why certain individuals are seropositive while others are not and whether there is an intrinsic driving factor that determines susceptibility for the virus. Within the subpopulation of exposed subjects a smaller subset excretes large amounts of JCPyV in the urine [31,32]. It is unclear why some subjects excrete the virus while others don’t. We therefore wanted to investigate whether miRNA expression levels in plasma are correlated to JCPyV serology or JCPyV viruria. In a first experiment 755 human miRNAs were analyzed in a small set of samples from seronegative subjects, shedders and non-shedder. Based on these results, 10 miRNAs were selected for further analysis and in a subsequent experiment another 100 samples were investigated for these 10 miRNAs. None of them were found to have differential expression between seropositive and seronegative subjects or between shedders and non-shedders. The fact that in the initial profiling experiment, none of the *p*-values remained significant (*p* < 0.05) after correction for multiple testing already indicated that the statistical significance of the differences observed was possibly caused by chance [33].

The lack of different miRNA patterns between seropositive and seronegative subjects is at first sight not that surprising as the presence or lack of antibodies against JCPyV might simply be explained by the fact that some subjects have and some other subjects have not been exposed to the virus. The lack of differential miRNA patterns between both groups might indeed be indicating that a subject’s JCPyV serostatus is driven by viral exposure and is only limitedly influenced by the background of the subject itself. One should however be mindful that JCPyV VP1 serology might actually underestimate real infection rates [12]. As a consequence this study would then effectively be comparing two groups of infected individuals, which may account for the lack of a difference. It should also be mentioned that to date 12 human polyomaviruses have been identified, all with high seroprevalence, making it likely that an individual is infected with several human polyomaviruses [34-37]. The differential plasma miRNA profile that may exist between JCPyV seropositive and seronegative individuals may therefore be masked by infection with other human polyomaviruses. Further studies might therefore require full human polyomavirus serotyping of the subjects examined.

The lack of different miRNA patterns between shedders and non-shedders on the other hand was more surprising as both groups have been exposed to the virus (both are seropositive) but only shedders appear to have active replication of the virus. One might have expected that some difference in biological or genetic background is needed to explain this observation. However, although limited to the analysis of circulating miRNAs only, our data presented in this study show this is not the case.

There is one observation that might be of interest for further study, even though it is not JCPyV related. Based on the analysis of hsa-miR-642, the population of 100 healthy subjects clearly can be separated in 2 groups, one with relatively high levels of hsa-miR-642 and one with low or undetectable levels of hsa-miR-642. Also, more female subjects appear to fall in the low expression group compared to male subjects. As it has been shown that hsa-miR-642 is an adipocyte-specific miRNA, it will be of interest to see whether this hsa-miR-642 based clustering is linked to specific metabolic parameters, such as Body Mass Index or blood cholesterol and fatty acids levels, or insulin resistance [38].

It should also be noted that only 755 miRNAs were analyzed in this study and it cannot be excluded that differential expression might be present for other miRNAs that were not analyzed here. Also, differences in circulating miRNAs that are expressed at low levels may have been missed. Other techniques that are not limited in the number of miRNAs, such as small RNA sequencing using deep sequencing platforms, have been shown to be very useful to investigate this [39,40]. Besides the use of a different analytical platform, one might also consider using PAXgene blood instead of plasma. This might show a totally different picture as it does not only include circulating miRNAs but also cellular miRNAs present in blood cells [41,42].

Although this study did not result in the identification of circulating miRNAs related to JCPyV infection, circulating miRNAs might still hold potential as biomarkers for PML progression in immunocompromised patients. It will therefore be of interest to investigate the miRNA expression profile of samples obtained from PML patients.

## Conclusions

Based on the experiments described in this work, we conclude that no differences in circulating human miRNAs can be observed between JCPyV seropositive subjects and JCPyV seronegative subjects on the one hand and between JCPyV shedders and non-shedders on the other hand.

## Abbreviations

PML: Progressive Multifocal Leukoencephalopathy; JCPyV: JC polyomavirus; MiRNA: microRNA; HSs: healthy subjects; qPCR: quantitative Polymerase Chain Reaction.

## Competing interests

Authors are current employees of Janssen Diagnostics BVBA or Janssen Research and Development, both being Johnson and Johnson Companies and may own stock or stock options in that company.

## Authors’ contributions

OL and LJS designed the study setup. OL performed the analysis of the data. TVL carried out the ELISA and viral load assays. LT and LJS contributed to revising the manuscript critically for important intellectual content and gave final approval of the version. All authors read and approved the final manuscript.

## Supplementary Material

Additional file 1: Table S1 List of 755 miRNA Assays and 3 controls (Assay IDs 001006, 001094 and 001973) used in the plasma miRNA profiling study. Click here for file

Additional file 2: Table S2 Raw Cq values of plasma miRNA profiling study. Click here for file

Additional file 3: Table S3 Raw Cq values of plasma miRNA validation/confirmation study. Click here for file
