# Supplementary material for: Circulating human microRNAs are not linked to JC polyomavirus serology or urinary viral load in healthy subjects
Source: Virol J. 2014 Mar 3;11:41. doi: 10.1186/1743-422X-11-41 (PMC3945012; doi:10.1186/1743-422X-11-41)
Supplement: Additional file 1: Table S1 — List of 755 miRNA Assays and 3 controls (Assay IDs 001006, 001094 and 001973) used in the plasma miRNA profiling study. [file 1743-422X-11-41-S1.docx]

Table S1 - List of 755 miRNA Assays and 3 controls (Assay IDs 001006, 001094 and 001973) used in the plasma miRNA profiling study.

| Assay ID | Assay Name | miRBase ID | miRBase Accession Number | miRBase Alias | Target Sequence |
| --- | --- | --- | --- | --- | --- |
| 000186 | mmu-miR-96 | bfl-miR-96-5p::bta-miR-96::ccr-miR-96::cfa-miR-96::dre-miR-96::eca-miR-96::fru-miR-96::hsa-miR-96-5p::mdo-miR-96::mmu-miR-96-5p::oan-miR-96-5p::ppy-miR-96::rno-miR-96-5p::tni-miR-96::xtr-miR-96 | MIMAT0000095::MIMAT0000541::MIMAT0000818::MIMAT0001811::MIMAT0003091::MIMAT0003092::MIMAT0003661::MIMAT0004147::MIMAT0006803::MIMAT0009388::MIMAT0009480::MIMAT0009861::MIMAT0012950::MIMAT0015745::MIMAT0026326 | bfl-miR-96(18)::hsa-miR-96(17)::mmu-miR-96(17)::oan-miR-96(18)::rno-miR-96(18) | UUUGGCACUAGCACAUUUUUGCU |
| 000268 | dme-miR-7 | aae-miR-7::aca-miR-7-5p::aga-miR-7::ame-miR-7::bfl-miR-7::bmo-miR-7-5p::cfa-miR-7::cgr-miR-7a::cqu-miR-7::csa-miR-7::dan-miR-7::der-miR-7::dgr-miR-7::dme-miR-7-5p::dmo-miR-7::dpe-miR-7::dps-miR-7::dpu-miR-7::dre-miR-7a::dse-miR-7::dsi-miR-7::dvi-miR-7::dwi-miR-7::dya-miR-7::eca-miR-7::hsa-miR-7-5p::isc-miR-7::mml-miR-7::mmu-miR-7a-5p::nlo-miR-7::nvi-miR-7::oan-miR-7-5p::pma-miR-7a-5p::ptr-miR-7::rno-miR-7a-5p::sko-miR-7-5p::spu-miR-7::tgu-miR-7-5p::tni-miR-7 | MIMAT0000112::MIMAT0000252::MIMAT0000606::MIMAT0000677::MIMAT0001209::MIMAT0001266::MIMAT0001489::MIMAT0001523::MIMAT0002485::MIMAT0003010::MIMAT0004192::MIMAT0006120::MIMAT0006159::MIMAT0006634::MIMAT0006986::MIMAT0008410::MIMAT0008537::MIMAT0008622::MIMAT0008692::MIMAT0008742::MIMAT0008771::MIMAT0008901::MIMAT0008931::MIMAT0009009::MIMAT0009119::MIMAT0009464::MIMAT0009608::MIMAT0009651::MIMAT0012666::MIMAT0012702::MIMAT0012905::MIMAT0014290::MIMAT0014423::MIMAT0014510::MIMAT0015659::MIMAT0018436::MIMAT0019370::MIMAT0020368::MIMAT0021983 | aca-miR-7(18)::bmo-miR-7(18)::cgr-miR-7(18)::dme-miR-7(16)::hsa-miR-7(17)::mmu-miR-7a(17)::oan-miR-7(18)::pma-miR-7a(18)::rno-miR-7a(18)::sko-miR-7(18)::tca-miR-7(16)::tgu-miR-7(18) | UGGAAGACUAGUGAUUUUGUUGU |
| 000338 | ath-miR159a | ahy-miR159::aly-miR159a-3p::ath-miR159a::bna-miR159::bra-miR159a::cme-miR159a::csi-miR159::gma-miR159a-3p::gma-miR159e-3p::hbr-miR159a::htu-miR159a::mes-miR159::mtr-miR159a::nta-miR159::ptc-miR159a::ptc-miR159b::ptc-miR159c::pvu-miR159a.1::rco-miR159::sly-miR159::vvi-miR159c | MIMAT0000177::MIMAT0001675::MIMAT0001901::MIMAT0001902::MIMAT0001903::MIMAT0005635::MIMAT0005650::MIMAT0009141::MIMAT0010153::MIMAT0011166::MIMAT0014152::MIMAT0015301::MIMAT0016321::MIMAT0017424::MIMAT0018450::MIMAT0021641::MIMAT0024410::MIMAT0024639::MIMAT0025283::MIMAT0025497::MIMAT0026160 | aly-miR159a(18)::gma-miR159(11)::gma-miR159a(17) | UUUGGAUUGAAGGGAGCUCUA |
| 000377 | hsa-let-7a | aca-let-7a-5p::asu-let-7-5p::bfl-let-7a-5p::bma-let-7::bta-let-7a-5p::cbr-let-7::ccr-let-7a::cel-let-7-5p::cfa-let-7a::cgr-let-7a::crm-let-7::dre-let-7a::eca-let-7a::fru-let-7a::gga-let-7a::gga-let-7j::hsa-let-7a-5p::lgi-let-7::mdo-let-7a::mml-let-7a::mmu-let-7a-5p::ola-let-7a::pma-let-7a::ppc-let-7::ppy-let-7a::ptr-let-7a::rno-let-7a-5p::sko-let-7::ssc-let-7a::tgu-let-7a-5p::tni-let-7a::xtr-let-7a | MIMAT0000001::MIMAT0000062::MIMAT0000463::MIMAT0000521::MIMAT0000774::MIMAT0001101::MIMAT0001181::MIMAT0001759::MIMAT0002928::MIMAT0002929::MIMAT0003667::MIMAT0003844::MIMAT0004141::MIMAT0006151::MIMAT0006594::MIMAT0007936::MIMAT0009462::MIMAT0009604::MIMAT0009644::MIMAT0011519::MIMAT0011657::MIMAT0012979::MIMAT0013865::MIMAT0014094::MIMAT0014519::MIMAT0015721::MIMAT0019564::MIMAT0021417::MIMAT0021692::MIMAT0022566::MIMAT0023716::MIMAT0026189 | aca-let-7a(18)::asu-let-7(18)::bfl-let-7(16)::bfl-let-7a(18)::bta-let-7a(18)::cel-let-7(17)::gga-let-7a-1(5)::gga-let-7a-2(5)::gga-let-7a-3(5)::hsa-let-7a(17)::mmu-let-7a(17)::rno-let-7a(18)::tgu-let-7a(18) | UGAGGUAGUAGGUUGUAUAGUU |
| 000379 | hsa-let-7c | aca-let-7c-5p::bta-let-7c::cfa-let-7c::dre-let-7c::eca-let-7c::gga-let-7c::hsa-let-7c::mml-let-7c::mmu-let-7c-5p::pol-let-7a-5p::ppy-let-7c::ptr-let-7c::rno-let-7c-5p::ssc-let-7c::tgu-let-7c::xtr-let-7c | MIMAT0000064::MIMAT0000523::MIMAT0000776::MIMAT0001104::MIMAT0001761::MIMAT0002151::MIMAT0003644::MIMAT0004332::MIMAT0006153::MIMAT0006669::MIMAT0007938::MIMAT0013181::MIMAT0014520::MIMAT0015723::MIMAT0021696::MIMAT0025416 | aca-let-7c(18)::mmu-let-7c(17)::rno-let-7c(18) | UGAGGUAGUAGGUUGUAUGGUU |
| 000382 | hsa-let-7f | aca-let-7f-5p::bta-let-7f::cfa-let-7f::cgr-let-7f::dre-let-7f::eca-let-7f::gga-let-7f::hsa-let-7f-5p::mdo-let-7f::mml-let-7f::mmu-let-7f-5p::oan-let-7f-5p::ppy-let-7f::ptr-let-7f::rno-let-7f-5p::ssc-let-7f::tgu-let-7f::xtr-let-7f | MIMAT0000067::MIMAT0000525::MIMAT0000778::MIMAT0001162::MIMAT0001764::MIMAT0002152::MIMAT0003519::MIMAT0003645::MIMAT0004161::MIMAT0006156::MIMAT0006610::MIMAT0007179::MIMAT0007941::MIMAT0013111::MIMAT0014530::MIMAT0015726::MIMAT0021703::MIMAT0023721 | aca-let-7f(18)::hsa-let-7f(17)::mmu-let-7f(17)::oan-let-7f(18)::rno-let-7f(18) | UGAGGUAGUAGAUUGUAUAGUU |
| 000387 | hsa-miR-10a | aca-miR-10a-5p::age-miR-10a::bfl-miR-10a-5p::bta-miR-10a::eca-miR-10a::ggo-miR-10a::hsa-miR-10a-5p::mdo-miR-10a::mml-miR-10a::mmu-miR-10a-5p::ppa-miR-10a::ppy-miR-10a::ptr-miR-10a::rno-miR-10a-5p::sko-miR-10::sla-miR-10a::xtr-miR-10a | MIMAT0000253::MIMAT0000648::MIMAT0000782::MIMAT0002486::MIMAT0002487::MIMAT0002488::MIMAT0002489::MIMAT0002490::MIMAT0003557::MIMAT0003786::MIMAT0004089::MIMAT0006161::MIMAT0007944::MIMAT0009466::MIMAT0009611::MIMAT0013019::MIMAT0021717 | aca-miR-10a(18)::bfl-miR-10a(18)::dre-miR-10a(6)::hsa-miR-10a(17)::mmu-miR-10a(17)::rno-miR-10a(9.2) | UACCCUGUAGAUCCGAAUUUGUG |
| 000389 | hsa-miR-15a | age-miR-15a::eca-miR-15a::ggo-miR-15a::hsa-miR-15a-5p::lca-miR-15a::lla-miR-15a::mml-miR-15a::mmu-miR-15a-5p::mne-miR-15a::ppa-miR-15a::ppy-miR-15a::ptr-miR-15a::sla-miR-15a::xtr-miR-15a | MIMAT0000068::MIMAT0000526::MIMAT0002638::MIMAT0002640::MIMAT0002642::MIMAT0002644::MIMAT0002646::MIMAT0002648::MIMAT0002650::MIMAT0002652::MIMAT0002654::MIMAT0002656::MIMAT0003560::MIMAT0013083 | gga-miR-15a(8.1)::hsa-miR-15a(17)::mmu-miR-15a(17) | UAGCAGCACAUAAUGGUUUGUG |
| 000390 | hsa-miR-15b | age-miR-15b::bta-miR-15b::cgr-miR-15b-5p::eca-miR-15b::ggo-miR-15b::hsa-miR-15b-5p::lla-miR-15b::mml-miR-15b::mmu-miR-15b-5p::mne-miR-15b::ppa-miR-15b::ppy-miR-15b::ptr-miR-15b::rno-miR-15b-5p::ssc-miR-15b | MIMAT0000124::MIMAT0000417::MIMAT0000784::MIMAT0002125::MIMAT0002202::MIMAT0002203::MIMAT0002204::MIMAT0002205::MIMAT0002206::MIMAT0002207::MIMAT0002208::MIMAT0002209::MIMAT0003792::MIMAT0012954::MIMAT0023791 | hsa-miR-15b(17)::mmu-miR-15b(17)::rno-miR-15b(18) | UAGCAGCACAUCAUGGUUUACA |
| 000391 | hsa-miR-16 | age-miR-16::cfa-miR-16::cgr-miR-16-5p::eca-miR-16::ggo-miR-16::hsa-miR-16-5p::lla-miR-16::mdo-miR-16::mml-miR-16::mmu-miR-16-5p::mne-miR-16::oan-miR-16a-5p::ppa-miR-16::ptr-miR-16::rno-miR-16-5p::sla-miR-16::ssc-miR-16 | MIMAT0000069::MIMAT0000527::MIMAT0000785::MIMAT0002639::MIMAT0002641::MIMAT0002643::MIMAT0002645::MIMAT0002647::MIMAT0002651::MIMAT0002655::MIMAT0002657::MIMAT0004145::MIMAT0006648::MIMAT0007166::MIMAT0007754::MIMAT0012955::MIMAT0023793 | hsa-miR-16(17)::mmu-miR-16(17)::oan-miR-16a(18)::ppy-miR-16(14)::rno-miR-16(18) | UAGCAGCACGUAAAUAUUGGCG |
| 000395 | hsa-miR-19a | age-miR-19a::bta-miR-19a::cfa-miR-19a::cgr-miR-19a::dre-miR-19a-3p::eca-miR-19a::fru-miR-19a::gga-miR-19a::ggo-miR-19a::hsa-miR-19a-3p::lca-miR-19a::lla-miR-19a::mdo-miR-19a::mml-miR-19a::mmu-miR-19a-3p::mne-miR-19a::oan-miR-19a-3p::ppa-miR-19a::ppy-miR-19a::ptr-miR-19a::rno-miR-19a-3p::sla-miR-19a::ssc-miR-19a::tni-miR-19a::xtr-miR-19a | MIMAT0000073::MIMAT0000651::MIMAT0000789::MIMAT0001112::MIMAT0001782::MIMAT0002128::MIMAT0002661::MIMAT0002668::MIMAT0002675::MIMAT0002682::MIMAT0002689::MIMAT0002696::MIMAT0002703::MIMAT0002710::MIMAT0002717::MIMAT0002724::MIMAT0002920::MIMAT0002921::MIMAT0003566::MIMAT0004168::MIMAT0004336::MIMAT0006650::MIMAT0007148::MIMAT0013086::MIMAT0023839 | dre-miR-19a(18)::hsa-miR-19a(17)::mmu-miR-19a(17)::oan-miR-19a(18)::rno-miR-19a(18) | UGUGCAAAUCUAUGCAAAACUGA |
| 000396 | hsa-miR-19b | age-miR-19b::bta-miR-19b::cgr-miR-19b-3p::dre-miR-19b-3p::eca-miR-19b::fru-miR-19b::gga-miR-19b::ggo-miR-19b::hsa-miR-19b-3p::lca-miR-19b::lla-miR-19b::mdo-miR-19b::mml-miR-19b::mmu-miR-19b-3p::mne-miR-19b::oan-miR-19b-3p::pma-miR-19b-3p::ppa-miR-19b::ppy-miR-19b::ptr-miR-19b::rno-miR-19b-3p::sla-miR-19b::ssc-miR-19b::tgu-miR-19b::tni-miR-19b::xla-miR-19b::xtr-miR-19b | MIMAT0000074::MIMAT0000513::MIMAT0000788::MIMAT0001110::MIMAT0001347::MIMAT0001783::MIMAT0002663::MIMAT0002670::MIMAT0002677::MIMAT0002684::MIMAT0002691::MIMAT0002698::MIMAT0002705::MIMAT0002712::MIMAT0002719::MIMAT0002726::MIMAT0002922::MIMAT0002923::MIMAT0003567::MIMAT0004170::MIMAT0004337::MIMAT0006856::MIMAT0013087::MIMAT0013950::MIMAT0014517::MIMAT0019393::MIMAT0023841 | dre-miR-19b(18)::hsa-miR-19b(17)::mmu-miR-19b(17)::oan-miR-19b(18)::pma-miR-19b(18)::rno-miR-19b(18) | UGUGCAAAUCCAUGCAAAACUGA |
| 000397 | hsa-miR-21 | aca-miR-21-5p::age-miR-21::cfa-miR-21::cgr-miR-21-5p::eca-miR-21::gga-miR-21::ggo-miR-21::hsa-miR-21-5p::mdo-miR-21::mml-miR-21::mmu-miR-21a-5p::mne-miR-21::oan-miR-21-5p::ppa-miR-21::ppy-miR-21::ptr-miR-21::rno-miR-21-5p::ssc-miR-21::tgu-miR-21-5p | MIMAT0000076::MIMAT0000530::MIMAT0000790::MIMAT0002165::MIMAT0002320::MIMAT0002321::MIMAT0002322::MIMAT0002323::MIMAT0002324::MIMAT0002325::MIMAT0002326::MIMAT0003774::MIMAT0004091::MIMAT0004417::MIMAT0006741::MIMAT0007160::MIMAT0013029::MIMAT0014527::MIMAT0021865 | aca-miR-21(18)::cgr-miR-21(18)::hsa-miR-21(17)::mmu-miR-21(17)::mmu-miR-21-5p(18)::oan-miR-21(18)::rno-miR-21(18)::tgu-miR-21(18) | UAGCUUAUCAGACUGAUGUUGA |
| 000398 | hsa-miR-22 | age-miR-22::cfa-miR-22::cgr-miR-22-3p::eca-miR-22::gga-miR-22-3p::hsa-miR-22-3p::lca-miR-22::lla-miR-22::mml-miR-22::mmu-miR-22-3p::mne-miR-22::oan-miR-22-3p::ppa-miR-22::ppy-miR-22::ptr-miR-22::rno-miR-22-3p::sla-miR-22::ssc-miR-22-3p::xtr-miR-22-3p | MIMAT0000077::MIMAT0000531::MIMAT0000791::MIMAT0002327::MIMAT0002328::MIMAT0002329::MIMAT0002330::MIMAT0002331::MIMAT0002332::MIMAT0002333::MIMAT0002334::MIMAT0002335::MIMAT0003672::MIMAT0006733::MIMAT0006962::MIMAT0007288::MIMAT0013031::MIMAT0015710::MIMAT0023857 | gga-miR-22(18)::hsa-miR-22(17)::mmu-miR-22(17)::oan-miR-22(18)::rno-miR-22(18)::xtr-miR-22(18) | AAGCUGCCAGUUGAAGAACUGU |
| 000399 | hsa-miR-23a | aca-miR-23a-3p::age-miR-23a::ccr-miR-23a::cgr-miR-23a-3p::eca-miR-23a::ggo-miR-23a::hsa-miR-23a-3p::lca-miR-23a::mml-miR-23a::mmu-miR-23a-3p::mne-miR-23a::ppa-miR-23a::ppy-miR-23a::ptr-miR-23a::rno-miR-23a-3p::sla-miR-23a::ssc-miR-23a::xla-miR-23a::xtr-miR-23a | MIMAT0000078::MIMAT0000532::MIMAT0000792::MIMAT0002133::MIMAT0002738::MIMAT0002741::MIMAT0002743::MIMAT0002745::MIMAT0002747::MIMAT0002749::MIMAT0002752::MIMAT0002754::MIMAT0002756::MIMAT0003568::MIMAT0011145::MIMAT0012986::MIMAT0021896::MIMAT0023863::MIMAT0026277 | aca-miR-23a(18)::hsa-miR-23a(17)::mmu-miR-23a(17)::rno-miR-23a(18) | AUCACAUUGCCAGGGAUUUCC |
| 000400 | hsa-miR-23b | aca-miR-23b-3p::ccr-miR-23b::cgr-miR-23b-3p::eca-miR-23b::gga-miR-23b::hsa-miR-23b-3p::mdo-miR-23b::mml-miR-23b::mmu-miR-23b-3p::rno-miR-23b-3p::tgu-miR-23-3p::xtr-miR-23b | MIMAT0000125::MIMAT0000418::MIMAT0000793::MIMAT0001186::MIMAT0003673::MIMAT0004176::MIMAT0006165::MIMAT0013113::MIMAT0014566::MIMAT0021898::MIMAT0023865::MIMAT0026278 | aca-miR-23b(18)::hsa-miR-23b(17)::mmu-miR-23b(17)::rno-miR-23b(18)::tgu-miR-23(18) | AUCACAUUGCCAGGGAUUACC |
| 000402 | hsa-miR-24 | aca-miR-24-3p::bta-miR-24-3p::ccr-miR-24::dre-miR-24::eca-miR-24::fru-miR-24-3p::gga-miR-24::ggo-miR-24::hsa-miR-24-3p::mml-miR-24-3p::mmu-miR-24-3p::mne-miR-24-3p::oan-miR-24-3p::ola-miR-24a::pma-miR-24::ppa-miR-24-3p::ppy-miR-24-3p::ptr-miR-24::rno-miR-24-3p::ssc-miR-24-3p::tgu-miR-24::tni-miR-24::xtr-miR-24a-3p | MIMAT0000080::MIMAT0000219::MIMAT0000794::MIMAT0001188::MIMAT0001792::MIMAT0002134::MIMAT0002337::MIMAT0002339::MIMAT0002341::MIMAT0002343::MIMAT0002740::MIMAT0002751::MIMAT0003065::MIMAT0003066::MIMAT0003654::MIMAT0003840::MIMAT0006955::MIMAT0012987::MIMAT0014539::MIMAT0019405::MIMAT0021900::MIMAT0022545::MIMAT0026279 | aca-miR-24(18)::bta-miR-24(11)::fru-miR-24(17)::hsa-miR-24(17)::mml-miR-24(18)::mmu-miR-24(17)::mne-miR-24(18)::oan-miR-24(18)::ppa-miR-24(18)::ppy-miR-24(18)::rno-miR-24(18)::ssc-miR-24(18)::xtr-miR-24a(17) | UGGCUCAGUUCAGCAGGAACAG |
| 000403 | hsa-miR-25 | bta-miR-25::ccr-miR-25::cfa-miR-25::cgr-miR-25-3p::dre-miR-25::eca-miR-25::fru-miR-25::ggo-miR-25::hsa-miR-25-3p::lla-miR-25::mdo-miR-25::mml-miR-25::mmu-miR-25-3p::mne-miR-25::ppa-miR-25::ppy-miR-25::rno-miR-25-3p::tni-miR-25::xtr-miR-25 | MIMAT0000081::MIMAT0000652::MIMAT0000795::MIMAT0001793::MIMAT0002760::MIMAT0002765::MIMAT0002768::MIMAT0002774::MIMAT0002779::MIMAT0002782::MIMAT0002914::MIMAT0002915::MIMAT0003674::MIMAT0003853::MIMAT0004179::MIMAT0006697::MIMAT0013057::MIMAT0023870::MIMAT0026280 | hsa-miR-25(17)::mmu-miR-25(17)::ptr-miR-25(9)::rno-miR-25(18) | CAUUGCACUUGUCUCGGUCUGA |
| 000405 | hsa-miR-26a | bta-miR-26a::ccr-miR-26a::cfa-miR-26a::dre-miR-26a::eca-miR-26a::fru-miR-26::ggo-miR-26a::hsa-miR-26a-5p::lla-miR-26a::mml-miR-26a::mmu-miR-26a-5p::mne-miR-26a::oan-miR-26-5p::pma-miR-26a-5p::ppa-miR-26a::ppy-miR-26a::ptr-miR-26a::rno-miR-26a-5p::ssc-miR-26a::tgu-miR-26::tni-miR-26 | MIMAT0000082::MIMAT0000533::MIMAT0000796::MIMAT0001794::MIMAT0002135::MIMAT0002344::MIMAT0002345::MIMAT0002346::MIMAT0002347::MIMAT0002348::MIMAT0002349::MIMAT0002350::MIMAT0003037::MIMAT0003038::MIMAT0003516::MIMAT0006595::MIMAT0006991::MIMAT0012975::MIMAT0014516::MIMAT0019408::MIMAT0026281 | gga-miR-26a(5)::hsa-miR-26a(17)::mmu-miR-26a(17)::oan-miR-26(18)::pma-miR-26a(18)::rno-miR-26a(18) | UUCAAGUAAUCCAGGAUAGGCU |
| 000407 | hsa-miR-26b | ggo-miR-26b::hsa-miR-26b-5p::mml-miR-26b::mmu-miR-26b-5p::ppy-miR-26b::ptr-miR-26b::rno-miR-26b-5p | MIMAT0000083::MIMAT0000534::MIMAT0000797::MIMAT0006166::MIMAT0008077::MIMAT0015733::MIMAT0024091 | hsa-miR-26b(17)::mmu-miR-26b(17)::rno-miR-26b(18) | UUCAAGUAAUUCAGGAUAGGU |
| 000408 | hsa-miR-27a | aca-miR-27a-3p::ccr-miR-27a::cgr-miR-27a-3p::eca-miR-27a::hsa-miR-27a-3p::mdo-miR-27a::mmu-miR-27a-3p::oan-miR-27a-3p::rno-miR-27a-3p::ssc-miR-27a::xtr-miR-27a | MIMAT0000084::MIMAT0000537::MIMAT0000799::MIMAT0002148::MIMAT0003570::MIMAT0004174::MIMAT0007063::MIMAT0012988::MIMAT0021906::MIMAT0023875::MIMAT0026282 | aca-miR-27a(18)::hsa-miR-27a(17)::mmu-miR-27a(17)::oan-miR-27a(18)::rno-miR-27a(18) | UUCACAGUGGCUAAGUUCCGC |
| 000409 | hsa-miR-27b | bta-miR-27b::cfa-miR-27b::cgr-miR-27b-3p::eca-miR-27b::gga-miR-27b::ggo-miR-27b::hsa-miR-27b-3p::mdo-miR-27b::mml-miR-27b::mmu-miR-27b-3p::oan-miR-27b-3p::pma-miR-27b-3p::ppy-miR-27b::ptr-miR-27b::rno-miR-27b-3p::sha-miR-27b::ssc-miR-27b-3p::tgu-miR-27::xtr-miR-27b | MIMAT0000126::MIMAT0000419::MIMAT0000798::MIMAT0001187::MIMAT0003546::MIMAT0003571::MIMAT0004177::MIMAT0006167::MIMAT0006613::MIMAT0006957::MIMAT0008078::MIMAT0013114::MIMAT0013890::MIMAT0014534::MIMAT0015734::MIMAT0019415::MIMAT0022789::MIMAT0023877::MIMAT0024112 | hsa-miR-27b(17)::mmu-miR-27b(17)::oan-miR-27b(18)::pma-miR-27b(18)::rno-miR-27b(18)::ssc-miR-27b(18) | UUCACAGUGGCUAAGUUCUGC |
| 000411 | hsa-miR-28 | age-miR-28::bta-miR-28::eca-miR-28-5p::ggo-miR-28::hsa-miR-28-5p::lla-miR-28::mml-miR-28::mmu-miR-28a-5p::mne-miR-28::ppa-miR-28::ppy-miR-28::ptr-miR-28::rno-miR-28-5p::sla-miR-28::ssc-miR-28-5p | MIMAT0000085::MIMAT0000653::MIMAT0000800::MIMAT0002136::MIMAT0002351::MIMAT0002352::MIMAT0002353::MIMAT0002354::MIMAT0002355::MIMAT0002356::MIMAT0002357::MIMAT0002358::MIMAT0002359::MIMAT0009272::MIMAT0013092 | hsa-miR-28(9.2)::mmu-miR-28(17)::mmu-miR-28-5p(18)::rno-miR-28(18) | AAGGAGCUCACAGUCUAUUGAG |
| 000413 | hsa-miR-29b | bta-miR-29b::ccr-miR-29b::cfa-miR-29b::cgr-miR-29b-3p::eca-miR-29b::gga-miR-29b::hsa-miR-29b-3p::mml-miR-29b::mmu-miR-29b-3p::rno-miR-29b-3p::ssc-miR-29b::xtr-miR-29b | MIMAT0000100::MIMAT0000127::MIMAT0000801::MIMAT0001097::MIMAT0002137::MIMAT0003573::MIMAT0003828::MIMAT0006168::MIMAT0006625::MIMAT0012941::MIMAT0023886::MIMAT0026287 | hsa-miR-29b(17)::mmu-miR-29b(17)::rno-miR-29b(18) | UAGCACCAUUUGAAAUCAGUGUU |
| 000416 | hsa-miR-30a-3p | aca-miR-30a-3p::cgr-miR-30a-3p::dre-miR-30e-3p::gga-miR-30a-3p::ggo-miR-30a-3p::hsa-miR-30a-3p::mml-miR-30a-3p::mmu-miR-30a-3p::oan-miR-30a-3p::ppa-miR-30a-3p::ppy-miR-30a-3p::ptr-miR-30a-3p::rno-miR-30a-3p::ssc-miR-30a-3p | MIMAT0000088::MIMAT0000129::MIMAT0000809::MIMAT0001136::MIMAT0002370::MIMAT0002372::MIMAT0002374::MIMAT0002376::MIMAT0002378::MIMAT0003402::MIMAT0006914::MIMAT0015300::MIMAT0021921::MIMAT0023895 | aca-miR-30a*(18)::dre-miR-30e*(18)::hsa-miR-30a(3.1)::hsa-miR-30a*(17)::mmu-miR-30a(3.1)::mmu-miR-30a*(17)::oan-miR-30a*(18)::rno-miR-30a(3.1)::rno-miR-30a*(18) | CUUUCAGUCGGAUGUUUGCAGC |
| 000417 | hsa-miR-30a-5p | aca-miR-30a-5p::gga-miR-30a-5p::ggo-miR-30a-5p::hsa-miR-30a-5p::mdo-miR-30a::mml-miR-30a-5p::mmu-miR-30a-5p::ppa-miR-30a-5p::ppy-miR-30a-5p::ptr-miR-30a-5p::rno-miR-30a-5p::ssc-miR-30a-5p::xtr-miR-30a-5p | MIMAT0000087::MIMAT0000128::MIMAT0000808::MIMAT0001135::MIMAT0002369::MIMAT0002371::MIMAT0002373::MIMAT0002375::MIMAT0002377::MIMAT0003574::MIMAT0004093::MIMAT0010193::MIMAT0021920 | aca-miR-30a(18)::hsa-miR-30a(17)::mmu-miR-30a(17)::rno-miR-30a(18)::ssc-miR-30a(14) | UGUAAACAUCCUCGACUGGAAG |
| 000419 | hsa-miR-30c | bta-miR-30c::cgr-miR-30c::eca-miR-30c::ggo-miR-30c::hsa-miR-30c-5p::lla-miR-30c::mml-miR-30c::mmu-miR-30c-5p::mne-miR-30c::ola-miR-30c::ppy-miR-30c::ptr-miR-30c::rno-miR-30c-5p::ssc-miR-30c-5p::xtr-miR-30c | MIMAT0000244::MIMAT0000514::MIMAT0000804::MIMAT0002167::MIMAT0002614::MIMAT0002615::MIMAT0002616::MIMAT0003577::MIMAT0003850::MIMAT0006170::MIMAT0012915::MIMAT0015737::MIMAT0022598::MIMAT0023898::MIMAT0024111 | gga-miR-30c(8.1)::gga-miR-30c-1(5)::gga-miR-30c-2(5)::hsa-miR-30c(17)::mmu-miR-30c(17)::rno-miR-30c(18)::ssc-miR-30c(18) | UGUAAACAUCCUACACUCUCAGC |
| 000420 | hsa-miR-30d | aca-miR-30d-5p::dre-miR-30d::eca-miR-30d::fru-miR-30d::gga-miR-30d::ggo-miR-30d::hsa-miR-30d-5p::mml-miR-30d::mmu-miR-30d-5p::mne-miR-30d::ppa-miR-30d::ppy-miR-30d::ptr-miR-30d::rno-miR-30d-5p::tni-miR-30d::xtr-miR-30d | MIMAT0000245::MIMAT0000515::MIMAT0000807::MIMAT0001129::MIMAT0001806::MIMAT0002617::MIMAT0002618::MIMAT0002619::MIMAT0002620::MIMAT0002949::MIMAT0002950::MIMAT0003657::MIMAT0006171::MIMAT0013006::MIMAT0015738::MIMAT0021926 | aca-miR-30d(18)::hsa-miR-30d(17)::mmu-miR-30d(17)::rno-miR-30d(18) | UGUAAACAUCCCCGACUGGAAG |
| 000422 | hsa-miR-30e-3p | aca-miR-30e-3p::cfa-miR-30e::cgr-miR-30e-3p::hsa-miR-30e-3p::mmu-miR-30e-3p::oan-miR-30e-3p::rno-miR-30e-3p::ssc-miR-30e-3p::tgu-miR-30a-3p | MIMAT0000249::MIMAT0000693::MIMAT0004720::MIMAT0006627::MIMAT0007204::MIMAT0013873::MIMAT0014535::MIMAT0021929::MIMAT0023901 | aca-miR-30e*(18)::hsa-miR-30e*(17)::mmu-miR-30e*(17)::oan-miR-30e*(18)::rno-miR-30e*(18) | CUUUCAGUCGGAUGUUUACAGC |
| 000426 | hsa-miR-34a | age-miR-34a::bta-miR-34a::ccr-miR-34::cfa-miR-34a::cgr-miR-34a::dre-miR-34a::eca-miR-34a::ggo-miR-34a::hsa-miR-34a-5p::lla-miR-34a::mml-miR-34a::mmu-miR-34a-5p::mne-miR-34a::ppa-miR-34a::ppy-miR-34a::ptr-miR-34a::rno-miR-34a-5p::sla-miR-34a::ssc-miR-34a::tgu-miR-34a | MIMAT0000255::MIMAT0000542::MIMAT0000815::MIMAT0001269::MIMAT0002494::MIMAT0002495::MIMAT0002496::MIMAT0002497::MIMAT0002498::MIMAT0002499::MIMAT0002500::MIMAT0002501::MIMAT0002502::MIMAT0004340::MIMAT0006690::MIMAT0007757::MIMAT0012917::MIMAT0014598::MIMAT0023926::MIMAT0026292 | dre-miR-34(18)::eca-miR-34(18)::gga-miR-34a(5)::hsa-miR-34a(17)::mmu-miR-34a(17)::rno-miR-34a(18) | UGGCAGUGUCUUAGCUGGUUGU |
| 000427 | hsa-miR-34b | hsa-miR-34b-5p | MIMAT0000685 | hsa-miR-34b(9.2)::hsa-miR-34b*(17) | UAGGCAGUGUCAUUAGCUGAUUG |
| 000428 | hsa-miR-34c | cfa-miR-34c::cgr-miR-34c-5p::eca-miR-34c::gga-miR-34c::hsa-miR-34c-5p::mml-miR-34c-5p::mmu-miR-34c-5p::oan-miR-34-5p::ppy-miR-34c-5p::rno-miR-34c-5p::ssc-miR-34c::tgu-miR-34b | MIMAT0000381::MIMAT0000686::MIMAT0000814::MIMAT0001180::MIMAT0006175::MIMAT0006693::MIMAT0007108::MIMAT0012992::MIMAT0013916::MIMAT0014537::MIMAT0015742::MIMAT0023929 | hsa-miR-34c(9.2)::mmu-miR-34c(17)::oan-miR-34(18)::rno-miR-34c(18) | AGGCAGUGUAGUUAGCUGAUUGC |
| 000431 | hsa-miR-92a | aca-miR-92a::age-miR-92::bta-miR-92a::ccr-miR-92a::cfa-miR-92a::cgr-miR-92a-3p::dre-miR-92a::eca-miR-92a::fru-miR-92::ggo-miR-92::hsa-miR-92a-3p::lca-miR-92::lla-miR-92::mml-miR-92a::mne-miR-92::oan-miR-92a-3p::ppa-miR-92::ppy-miR-92::ptr-miR-92::sla-miR-92::ssc-miR-92a::tgu-miR-92::tni-miR-92 | MIMAT0000092::MIMAT0001808::MIMAT0002664::MIMAT0002671::MIMAT0002678::MIMAT0002685::MIMAT0002692::MIMAT0002699::MIMAT0002706::MIMAT0002713::MIMAT0002720::MIMAT0002803::MIMAT0002924::MIMAT0002925::MIMAT0006653::MIMAT0006858::MIMAT0009383::MIMAT0013089::MIMAT0013908::MIMAT0014574::MIMAT0021993::MIMAT0024010::MIMAT0026323 | bta-miR-92(18)::gga-miR-92(5)::hsa-miR-92(5)::hsa-miR-92a(17)::mml-miR-92(10.1)::oan-miR-92a(18) | UAUUGCACUUGUCCCGGCCUGU |
| 000433 | hsa-miR-95 | bta-miR-95::cfa-miR-95::ggo-miR-95::hsa-miR-95::lla-miR-95::mml-miR-95::ppa-miR-95::ppy-miR-95::ptr-miR-95::sla-miR-95::ssc-miR-95 | MIMAT0000094::MIMAT0002142::MIMAT0002398::MIMAT0002399::MIMAT0002400::MIMAT0002401::MIMAT0002402::MIMAT0002403::MIMAT0006178::MIMAT0009387::MIMAT0009878 | | UUCAACGGGUAUUUAUUGAGCA |
| 000435 | hsa-miR-99a | aca-miR-99a-5p::dre-miR-99::eca-miR-99a::gga-miR-99a-5p::ggo-miR-99a::hsa-miR-99a-5p::lla-miR-99a::mml-miR-99a::mmu-miR-99a-5p::mne-miR-99a::ppa-miR-99a::ppy-miR-99a::ptr-miR-99a::rno-miR-99a-5p::ssc-miR-99a::xtr-miR-99 | MIMAT0000097::MIMAT0000131::MIMAT0000820::MIMAT0001103::MIMAT0001812::MIMAT0002410::MIMAT0002411::MIMAT0002412::MIMAT0002413::MIMAT0002414::MIMAT0002415::MIMAT0002416::MIMAT0003582::MIMAT0013184::MIMAT0013896::MIMAT0021996 | aca-miR-99a(18)::gga-miR-99a(18)::hsa-miR-99a(17)::mmu-miR-99a(17)::rno-miR-99a(18) | AACCCGUAGAUCCGAUCUUGUG |
| 000436 | hsa-miR-99b | bta-miR-99b::cfa-miR-99b::eca-miR-99b::hsa-miR-99b-5p::mml-miR-99b::mmu-miR-99b-5p::ptr-miR-99b::rno-miR-99b-5p::ssc-miR-99b | MIMAT0000132::MIMAT0000689::MIMAT0000821::MIMAT0004345::MIMAT0006018::MIMAT0006179::MIMAT0006607::MIMAT0008351::MIMAT0013018 | hsa-miR-99b(17)::mmu-miR-99b(17)::rno-miR-99b(18) | CACCCGUAGAACCGACCUUGCG |
| 000437 | hsa-miR-100 | aae-miR-100::aca-miR-100::aga-miR-100::age-miR-100::ame-miR-100::bma-miR-100b::bmo-miR-100::bta-miR-100::cqu-miR-100-5p::dre-miR-100::eca-miR-100::fru-miR-100::gga-miR-100::ggo-miR-100::hsa-miR-100-5p::lgi-miR-100::lla-miR-100::mml-miR-100::mmu-miR-100-5p::mse-miR-100::nvi-miR-100::oan-miR-100-5p::pma-miR-100a-5p::ppa-miR-100::ppy-miR-100::ptr-miR-100::rno-miR-100-5p::sko-miR-100::sla-miR-100::ssc-miR-100::tni-miR-100::xtr-miR-100 | MIMAT0000098::MIMAT0000655::MIMAT0000822::MIMAT0001178::MIMAT0001498::MIMAT0001813::MIMAT0002417::MIMAT0002418::MIMAT0002419::MIMAT0002420::MIMAT0002421::MIMAT0002422::MIMAT0002423::MIMAT0002424::MIMAT0003063::MIMAT0003064::MIMAT0003682::MIMAT0004420::MIMAT0007046::MIMAT0009215::MIMAT0009577::MIMAT0009623::MIMAT0012980::MIMAT0013596::MIMAT0013911::MIMAT0014113::MIMAT0014291::MIMAT0014387::MIMAT0015677::MIMAT0019437::MIMAT0021709::MIMAT0024438 | cqu-miR-100(18)::hsa-miR-100(17)::mmu-miR-100(17)::oan-miR-100(18)::pma-miR-100a(18)::rno-miR-100(18)::tca-miR-100(16) | AACCCGUAGAUCCGAACUUGUG |
| 000439 | hsa-miR-103 | age-miR-103::bta-miR-103::ccr-miR-103::cfa-miR-103::cgr-miR-103-3p::dre-miR-103::eca-miR-103::eca-miR-107a::fru-miR-103::gga-miR-103::ggo-miR-103::hsa-miR-103a-3p::lla-miR-103::mdo-miR-103::mml-miR-103::mmu-miR-103-3p::mne-miR-103::oan-miR-103-3p::pma-miR-103b-3p::ppa-miR-103::ppy-miR-103::ptr-miR-103::rno-miR-103-3p::ssc-miR-103::tgu-miR-103::tni-miR-103::xtr-miR-103 | MIMAT0000101::MIMAT0000546::MIMAT0000824::MIMAT0001145::MIMAT0001816::MIMAT0002154::MIMAT0002442::MIMAT0002443::MIMAT0002444::MIMAT0002445::MIMAT0002446::MIMAT0002447::MIMAT0002448::MIMAT0002449::MIMAT0003041::MIMAT0003042::MIMAT0003521::MIMAT0003663::MIMAT0004099::MIMAT0006687::MIMAT0006942::MIMAT0013061::MIMAT0013105::MIMAT0014523::MIMAT0019443::MIMAT0023731::MIMAT0026198 | gga-miR-103-1(5)::gga-miR-103-2(5)::hsa-miR-103(16)::hsa-miR-103a(17)::mmu-miR-103(17)::oan-miR-103(18)::pma-miR-103b(18)::rno-miR-103(18) | AGCAGCAUUGUACAGGGCUAUGA |
| 000442 | hsa-miR-106b | age-miR-106b::bta-miR-106b::cfa-miR-106b::eca-miR-106b::ggo-miR-106b::hsa-miR-106b-5p::lla-miR-106b::mml-miR-106b::mmu-miR-106b-5p::mne-miR-106b::ppa-miR-106b::ppy-miR-106b::ptr-miR-106b::rno-miR-106b-5p::sla-miR-106b | MIMAT0000386::MIMAT0000680::MIMAT0000825::MIMAT0002758::MIMAT0002761::MIMAT0002763::MIMAT0002766::MIMAT0002769::MIMAT0002772::MIMAT0002775::MIMAT0002777::MIMAT0002780::MIMAT0006695::MIMAT0009218::MIMAT0013054 | hsa-miR-106b(17)::mmu-miR-106b(17)::rno-miR-106b(18) | UAAAGUGCUGACAGUGCAGAU |
| 000443 | hsa-miR-107 | dre-miR-107a::eca-miR-107b::fru-miR-107::gga-miR-107::ggo-miR-107::hsa-miR-107::lla-miR-107::mdo-miR-107::mml-miR-107::mmu-miR-107-3p::mne-miR-107::ppa-miR-107::ppy-miR-107::ptr-miR-107::rno-miR-107-3p::ssc-miR-107::tgu-miR-107::tni-miR-107::xtr-miR-107 | MIMAT0000104::MIMAT0000647::MIMAT0000826::MIMAT0001147::MIMAT0001817::MIMAT0002155::MIMAT0002458::MIMAT0002459::MIMAT0002460::MIMAT0002461::MIMAT0002462::MIMAT0002463::MIMAT0002464::MIMAT0003043::MIMAT0003044::MIMAT0003584::MIMAT0004100::MIMAT0012886::MIMAT0014556 | dre-miR-107(18)::mmu-miR-107(17)::rno-miR-107(18) | AGCAGCAUUGUACAGGGCUAUCA |
| 000449 | hsa-miR-125b | aae-miR-125-5p::aca-miR-125b::aga-miR-125::age-miR-125b::bfl-miR-125a-5p::bta-miR-125b::ccr-miR-125b::cfa-miR-125b::cgr-miR-125b-5p::cqu-miR-125-5p::cte-miR-125::dan-miR-125::der-miR-125::dgr-miR-125::dme-miR-125-5p::dmo-miR-125::dpe-miR-125::dps-miR-125::dre-miR-125b::dse-miR-125::dsi-miR-125::dvi-miR-125::dwi-miR-125::dya-miR-125::eca-miR-125b-5p::fru-miR-125b::gga-miR-125b::ggo-miR-125b::hsa-miR-125b-5p::lca-miR-125b::lla-miR-125b::mdo-miR-125b::mml-miR-125b::mmu-miR-125b-5p::mne-miR-125b::nlo-miR-125::nvi-miR-125::oan-miR-125-5p::ola-miR-125b::pma-miR-125-5p::ppa-miR-125b::ppy-miR-125b::ptr-miR-125b::rno-miR-125b-5p::sha-miR-125a::sko-miR-125a::sla-miR-125b::spu-miR-125-5p::ssc-miR-125b::tca-miR-125-5p::tgu-miR-125-5p::tni-miR-125b::xtr-miR-125b | MIMAT0000136::MIMAT0000397::MIMAT0000423::MIMAT0000830::MIMAT0001105::MIMAT0001230::MIMAT0001500::MIMAT0001821::MIMAT0002120::MIMAT0002220::MIMAT0002221::MIMAT0002222::MIMAT0002223::MIMAT0002224::MIMAT0002225::MIMAT0002226::MIMAT0002227::MIMAT0002228::MIMAT0002280::MIMAT0003061::MIMAT0003062::MIMAT0003539::MIMAT0003586::MIMAT0004103::MIMAT0006670::MIMAT0007048::MIMAT0008371::MIMAT0008473::MIMAT0008526::MIMAT0008610::MIMAT0008669::MIMAT0008725::MIMAT0008826::MIMAT0008846::MIMAT0008917::MIMAT0009032::MIMAT0009096::MIMAT0009483::MIMAT0009525::MIMAT0009626::MIMAT0009667::MIMAT0012981::MIMAT0014258::MIMAT0014355::MIMAT0014505::MIMAT0015679::MIMAT0018421::MIMAT0019446::MIMAT0021729::MIMAT0022627::MIMAT0022802::MIMAT0023743::MIMAT0026204 | aae-miR-125(18)::aae-miR-125*(18)::bfl-miR-125(16)::bfl-miR-125a(18)::cap-miR-125(14)::cqu-miR-125(18)::dme-miR-125(16)::hsa-miR-125b(17)::mmu-miR-125b(9.2)::oan-miR-125(18)::pma-miR-125(18)::rno-miR-125b(9.2)::sko-miR-125(16)::spu-miR-125(18)::tca-miR-125(16)::tgu-miR-125(18) | UCCCUGAGACCCUAACUUGUGA |
| 000451 | hsa-miR-126# | aca-miR-126-5p::bta-miR-126-5p::ccr-miR-126-5p::cfa-miR-126::dre-miR-126a-5p::dre-miR-126b-5p::eca-miR-126-5p::gga-miR-126-5p::hsa-miR-126-5p::mdo-miR-126::mmu-miR-126-5p::oan-miR-126-5p::ola-miR-126-5p::rno-miR-126a-5p::ssc-miR-126-5p::xtr-miR-126-5p | MIMAT0000137::MIMAT0000444::MIMAT0000831::MIMAT0003157::MIMAT0003587::MIMAT0003723::MIMAT0004328::MIMAT0006730::MIMAT0007125::MIMAT0011307::MIMAT0012743::MIMAT0013176::MIMAT0018377::MIMAT0021730::MIMAT0022602::MIMAT0026206 | aca-miR-126*(18)::bta-miR-126*(18)::dre-miR-126*(16)::dre-miR-126a*(18)::gga-miR-126*(18)::hsa-miR-126*(17)::mmu-miR-126*(5)::oan-miR-126*(18)::rno-miR-126*(18)::ssc-miR-126*(18)::xtr-miR-126*(18) | CAUUAUUACUUUUGGUACGCG |
| 000452 | hsa-miR-127 | age-miR-127::bta-miR-127::cfa-miR-127::eca-miR-127::hsa-miR-127-3p::lla-miR-127::mml-miR-127::mmu-miR-127-3p::mne-miR-127::ppy-miR-127::ptr-miR-127::rno-miR-127-3p::sla-miR-127::ssc-miR-127 | MIMAT0000139::MIMAT0000446::MIMAT0000833::MIMAT0002281::MIMAT0002282::MIMAT0002283::MIMAT0002284::MIMAT0002285::MIMAT0002286::MIMAT0002287::MIMAT0003787::MIMAT0006713::MIMAT0013126::MIMAT0013932 | hsa-miR-127(9.2)::mmu-miR-127(17)::rno-miR-127(18) | UCGGAUCCGUCUGAGCUUGGCU |
| 000454 | hsa-miR-130a | bta-miR-130a::ccr-miR-130a::cfa-miR-130a::cgr-miR-130a-3p::dre-miR-130a::eca-miR-130a::gga-miR-130c-3p::hsa-miR-130a-3p::mmu-miR-130a-3p::oan-miR-130b-3p::ppy-miR-130a::ptr-miR-130a::rno-miR-130a-3p::ssc-miR-130a::tgu-miR-130c::xtr-miR-130a | MIMAT0000141::MIMAT0000425::MIMAT0000836::MIMAT0001826::MIMAT0003591::MIMAT0006631::MIMAT0007159::MIMAT0007734::MIMAT0007758::MIMAT0008025::MIMAT0009223::MIMAT0013047::MIMAT0014581::MIMAT0015753::MIMAT0023755::MIMAT0026210 | gga-miR-130c(18)::hsa-miR-130a(17)::mmu-miR-130a(17)::oan-miR-130b(18)::rno-miR-130a(18) | CAGUGCAAUGUUAAAAGGGCAU |
| 000456 | hsa-miR-130b | bta-miR-130b::cfa-miR-130b::cgr-miR-130b-3p::eca-miR-130b::hsa-miR-130b-3p::mml-miR-130b::mmu-miR-130b-3p::ppy-miR-130b::ptr-miR-130b::rno-miR-130b-3p::ssc-miR-130b::xtr-miR-130b | MIMAT0000387::MIMAT0000691::MIMAT0000837::MIMAT0003593::MIMAT0006187::MIMAT0006659::MIMAT0008026::MIMAT0009224::MIMAT0012996::MIMAT0013922::MIMAT0015754::MIMAT0023757 | hsa-miR-130b(17)::mmu-miR-130b(17)::rno-miR-130b(18) | CAGUGCAAUGAUGAAAGGGCAU |
| 000457 | hsa-miR-132 | bta-miR-132::ccr-miR-132a::cgr-miR-132-3p::dre-miR-132-3p::eca-miR-132::fru-miR-132::hsa-miR-132-3p::mdo-miR-132::mml-miR-132::mmu-miR-132-3p::ppy-miR-132::rno-miR-132-3p::ssc-miR-132::tgu-miR-132::tni-miR-132::xtr-miR-132 | MIMAT0000144::MIMAT0000426::MIMAT0000838::MIMAT0001829::MIMAT0003059::MIMAT0003060::MIMAT0003594::MIMAT0003812::MIMAT0004149::MIMAT0006188::MIMAT0013021::MIMAT0015755::MIMAT0018619::MIMAT0023759::MIMAT0025361::MIMAT0026213 | dre-miR-132(18)::hsa-miR-132(17)::mmu-miR-132(17)::rno-miR-132(18) | UAACAGUCUACAGCCAUGGUCG |
| 000460 | hsa-miR-135a | aca-miR-135-5p::age-miR-135::bta-miR-135a::cfa-miR-135a-5p::dre-miR-135a::eca-miR-135a::gga-miR-135a::ggo-miR-135a::hsa-miR-135a-5p::lla-miR-135::mdo-miR-135a::mml-miR-135a::mmu-miR-135a-5p::oan-miR-135b-5p::pma-miR-135a-5p::ppa-miR-135::ppy-miR-135a::ptr-miR-135a::rno-miR-135a-5p::ssc-miR-135::tgu-miR-135a-5p::tgu-miR-135b::xtr-miR-135 | MIMAT0000147::MIMAT0000428::MIMAT0000841::MIMAT0001099::MIMAT0002121::MIMAT0002122::MIMAT0002248::MIMAT0002249::MIMAT0002250::MIMAT0002251::MIMAT0002252::MIMAT0002253::MIMAT0003348::MIMAT0003598::MIMAT0004108::MIMAT0006870::MIMAT0009228::MIMAT0010196::MIMAT0013078::MIMAT0014559::MIMAT0014560::MIMAT0015757::MIMAT0019463::MIMAT0021750 | aca-miR-135(18)::gga-miR-135a-1(5)::gga-miR-135a-2(5)::gga-miR-135a-3(5)::ggo-miR-135(18)::hsa-miR-135(3)::hsa-miR-135a(17)::mml-miR-135(10.1)::mmu-miR-135(3)::mmu-miR-135a(17)::oan-miR-135b(18)::pma-miR-135a(18)::ppy-miR-135(18)::ptr-miR-135(11)::rno-miR-135a(18)::ssc-miR-135a-1(8.1)::ssc-miR-135a-2(8.1)::tgu-miR-135a(18) | UAUGGCUUUUUAUUCCUAUGUGA |
| 000463 | hsa-miR-141 | bta-miR-141::eca-miR-141::hsa-miR-141-3p::mmu-miR-141-3p::ptr-miR-141::rno-miR-141-3p | MIMAT0000153::MIMAT0000432::MIMAT0000846::MIMAT0008036::MIMAT0009232::MIMAT0012970 | hsa-miR-141(17)::mmu-miR-141(17)::rno-miR-141(18) | UAACACUGUCUGGUAAAGAUGG |
| 000464 | hsa-miR-142-3p | dre-miR-142a-3p::eca-miR-142-3p::hsa-miR-142-3p::mdo-miR-142::mml-miR-142-3p::mmu-miR-142-3p::ppy-miR-142-3p::ptr-miR-142::rno-miR-142-3p::xtr-miR-142-3p | MIMAT0000155::MIMAT0000434::MIMAT0000848::MIMAT0003160::MIMAT0003603::MIMAT0004112::MIMAT0006200::MIMAT0008037::MIMAT0013023::MIMAT0015766 | gga-miR-142-3p(8.1) | UGUAGUGUUUCCUACUUUAUGGA |
| 000468 | hsa-miR-146a | cfa-miR-146a::cgr-miR-146a::eca-miR-146a::gga-miR-146a::hsa-miR-146a-5p::mdo-miR-146a::mml-miR-146a::mmu-miR-146a-5p::ppy-miR-146a::ptr-miR-146a::rno-miR-146a-5p::ssc-miR-146a-5p::tgu-miR-146c | MIMAT0000158::MIMAT0000449::MIMAT0000852::MIMAT0001163::MIMAT0006203::MIMAT0006684::MIMAT0008038::MIMAT0012746::MIMAT0013065::MIMAT0014609::MIMAT0015767::MIMAT0022963::MIMAT0023774 | gga-miR-146(7.1)::hsa-miR-146(6)::hsa-miR-146a(17)::mmu-miR-146(9.2)::mmu-miR-146a(17)::rno-miR-146(9.2)::rno-miR-146a(18) | UGAGAACUGAAUUCCAUGGGUU |
| 000469 | hsa-miR-147 | hsa-miR-147a::mne-miR-147::ppa-miR-147::ppy-miR-147a::ptr-miR-147a::sla-miR-147 | MIMAT0000251::MIMAT0002474::MIMAT0002475::MIMAT0002476::MIMAT0002477::MIMAT0002478 | hsa-miR-147(17)::ppy-miR-147(18)::ptr-miR-147(11) | GUGUGUGGAAAUGCUUCUGC |
| 000470 | hsa-miR-148a | aca-miR-148a-3p::bta-miR-148a::cfa-miR-148a::eca-miR-148a::gga-miR-148a::hsa-miR-148a-3p::mdo-miR-148::mml-miR-148a::mmu-miR-148a-3p::oan-miR-148-3p::ppy-miR-148a::ptr-miR-148a::ssc-miR-148a-3p::tgu-miR-148::xtr-miR-148a | MIMAT0000243::MIMAT0000516::MIMAT0001120::MIMAT0002124::MIMAT0003522::MIMAT0003664::MIMAT0006208::MIMAT0006622::MIMAT0007138::MIMAT0008041::MIMAT0012737::MIMAT0012935::MIMAT0014503::MIMAT0015771::MIMAT0021781 | aca-miR-148a(18)::hsa-miR-148(3)::hsa-miR-148a(17)::mmu-miR-148a(17)::oan-miR-148(18)::ssc-miR-148a(18) | UCAGUGCACUACAGAACUUUGU |
| 000471 | hsa-miR-148b | aca-miR-148b-3p::bta-miR-148b::cfa-miR-148b::cgr-miR-148b-3p::eca-miR-148b-3p::hsa-miR-148b-3p::mml-miR-148b::mmu-miR-148b-3p::ppy-miR-148b::ptr-miR-148b::rno-miR-148b-3p::ssc-miR-148b-3p::xtr-miR-148b | MIMAT0000579::MIMAT0000580::MIMAT0000759::MIMAT0003605::MIMAT0003814::MIMAT0006209::MIMAT0006663::MIMAT0008042::MIMAT0012972::MIMAT0013901::MIMAT0015772::MIMAT0021783::MIMAT0023779 | aca-miR-148b(18)::hsa-miR-148b(17)::mmu-miR-148b(17)::rno-miR-148b(9.2)::ssc-miR-148b(18) | UCAGUGCAUCACAGAACUUUGU |
| 000473 | hsa-miR-150 | aca-miR-150-5p::cfa-miR-150::eca-miR-150::ggo-miR-150::hsa-miR-150-5p::mml-miR-150::mmu-miR-150-5p::ppy-miR-150::ptr-miR-150::rno-miR-150-5p::ssc-miR-150 | MIMAT0000160::MIMAT0000451::MIMAT0000853::MIMAT0006211::MIMAT0006602::MIMAT0008044::MIMAT0013011::MIMAT0015774::MIMAT0021784::MIMAT0024217::MIMAT0025365 | aca-miR-150(18)::hsa-miR-150(17)::mmu-miR-150(17)::rno-miR-150(18) | UCUCCCAACCCUUGUACCAGUG |
| 000475 | hsa-miR-152 | cfa-miR-152::cgr-miR-152-3p::ggo-miR-152::hsa-miR-152::mml-miR-152::mmu-miR-152-3p::ppy-miR-152::ptr-miR-152::rno-miR-152-3p::ssc-miR-152 | MIMAT0000162::MIMAT0000438::MIMAT0000854::MIMAT0006214::MIMAT0006738::MIMAT0008046::MIMAT0013887::MIMAT0023785::MIMAT0024099::MIMAT0024356 | mmu-miR-152(17)::rno-miR-152(18) | UCAGUGCAUGACAGAACUUGG |
| 000477 | hsa-miR-154 | cgr-miR-154-5p::eca-miR-154::ggo-miR-154::hsa-miR-154-5p::mml-miR-154::mmu-miR-154-5p::mne-miR-154::ppa-miR-154::ppy-miR-154::ptr-miR-154::rno-miR-154-5p | MIMAT0000164::MIMAT0000452::MIMAT0000856::MIMAT0002296::MIMAT0002297::MIMAT0002298::MIMAT0002299::MIMAT0002300::MIMAT0006215::MIMAT0013129::MIMAT0023786 | hsa-miR-154(17)::mmu-miR-154(17)::rno-miR-154(18) | UAGGUUAUCCGUGUUGCCUUCG |
| 000478 | hsa-miR-154# | cgr-miR-154-3p::hsa-miR-154-3p::mmu-miR-154-3p::rno-miR-154-3p | MIMAT0000453::MIMAT0004537::MIMAT0017136::MIMAT0023787 | hsa-miR-154*(17)::mmu-miR-154*(17)::rno-miR-154*(18) | AAUCAUACACGGUUGACCUAUU |
| 000480 | hsa-miR-181a | aca-miR-181a::cgr-miR-181a-5p::dre-miR-181a-5p::eca-miR-181a::fru-miR-181a-5p::gga-miR-181a-5p::ggo-miR-181a-5p::hsa-miR-181a-5p::lla-miR-181a-5p::mdo-miR-181a::mdo-miR-181c::mml-miR-181a-5p::mmu-miR-181a-5p::mne-miR-181a-5p::oan-miR-181a-5p::pma-miR-181a-5p::ppa-miR-181a-5p::ppy-miR-181a-5p::ptr-miR-181a-5p::rno-miR-181a-5p::sla-miR-181a::tgu-miR-181a-5p::tni-miR-181a-5p::xtr-miR-181a-5p | MIMAT0000210::MIMAT0000256::MIMAT0000858::MIMAT0001168::MIMAT0001623::MIMAT0002503::MIMAT0002504::MIMAT0002505::MIMAT0002506::MIMAT0002507::MIMAT0002508::MIMAT0002626::MIMAT0002631::MIMAT0003050::MIMAT0003111::MIMAT0003625::MIMAT0004121::MIMAT0004154::MIMAT0006833::MIMAT0013178::MIMAT0014546::MIMAT0019486::MIMAT0021809::MIMAT0023797 | dre-miR-181a(18)::fru-miR-181a(18)::gga-miR-181a(18)::ggo-miR-181a(18)::hsa-miR-181a(17)::lla-miR-181a(18)::mml-miR-181a(18)::mmu-miR-181a(17)::mne-miR-181a(18)::oan-miR-181a(18)::pma-miR-181a(18)::ppa-miR-181a(18)::ppy-miR-181a(18)::ptr-miR-181a(18)::rno-miR-181a(18)::tgu-miR-181a(18)::tni-miR-181a(18)::xtr-miR-181a(18) | AACAUUCAACGCUGUCGGUGAGU |
| 000482 | hsa-miR-181c | cgr-miR-181c-5p::ggo-miR-181c::hsa-miR-181c-5p::mml-miR-181c::mmu-miR-181c-5p::ppa-miR-181c::ppy-miR-181c::ptr-miR-181c::rno-miR-181c-5p::ssc-miR-181c | MIMAT0000258::MIMAT0000674::MIMAT0000857::MIMAT0002144::MIMAT0002509::MIMAT0002510::MIMAT0002511::MIMAT0002512::MIMAT0015778::MIMAT0023801 | hsa-miR-181c(17)::mmu-miR-181c(17)::rno-miR-181c(18) | AACAUUCAACCUGUCGGUGAGU |
| 000483 | hsa-miR-182# | dre-miR-182-3p::fru-miR-182::hsa-miR-182-3p::pol-miR-182-3p::tni-miR-182::xtr-miR-182-3p | MIMAT0000260::MIMAT0001272::MIMAT0003093::MIMAT0003094::MIMAT0003611::MIMAT0025439 | dre-miR-182*(18)::hsa-miR-182*(17)::xtr-miR-182*(18) | UGGUUCUAGACUUGCCAACUA |
| 000485 | hsa-miR-184 | aca-miR-184-3p::bta-miR-184::cfa-miR-184::cgr-miR-184::eca-miR-184::gga-miR-184::hsa-miR-184::mdo-miR-184::mml-miR-184::mmu-miR-184-3p::mne-miR-184::ppy-miR-184::ptr-miR-184::rno-miR-184::ssc-miR-184::tgu-miR-184 | MIMAT0000213::MIMAT0000454::MIMAT0000861::MIMAT0001158::MIMAT0002127::MIMAT0002301::MIMAT0002302::MIMAT0002303::MIMAT0004119::MIMAT0006218::MIMAT0009246::MIMAT0009842::MIMAT0012894::MIMAT0014561::MIMAT0021814::MIMAT0023809 | aca-miR-184(18)::mmu-miR-184(17) | UGGACGGAGAACUGAUAAGGGU |
| 000489 | hsa-miR-190 | bta-miR-190a::cfa-miR-190a::dre-miR-190a::eca-miR-190a::fru-miR-190::gga-miR-190::ggo-miR-190a::hsa-miR-190a::mdo-miR-190a::mml-miR-190a::mmu-miR-190a-5p::oan-miR-190a-5p::ppa-miR-190::ppy-miR-190a::ptr-miR-190a::rno-miR-190a-5p::sha-miR-190::tgu-miR-190::tni-miR-190 | MIMAT0000220::MIMAT0000458::MIMAT0000865::MIMAT0001155::MIMAT0001854::MIMAT0002312::MIMAT0002313::MIMAT0002314::MIMAT0002315::MIMAT0003109::MIMAT0003110::MIMAT0006843::MIMAT0009251::MIMAT0009872::MIMAT0012740::MIMAT0012896::MIMAT0014589::MIMAT0015783::MIMAT0022769 | dre-miR-190(18)::eca-miR-190(18)::ggo-miR-190(18)::hsa-miR-190(17)::mml-miR-190(10.1)::mmu-miR-190(17)::mmu-miR-190-5p(18)::oan-miR-190a(18)::ppy-miR-190(18)::ptr-miR-190(11)::rno-miR-190(18) | UGAUAUGUUUGAUAUAUUAGGU |
| 000491 | hsa-miR-192 | cfa-miR-192::eca-miR-192::ggo-miR-192::hsa-miR-192-5p::mml-miR-192::mmu-miR-192-5p::oan-miR-192-5p::ppy-miR-192::ptr-miR-192::rno-miR-192-5p::ssc-miR-192 | MIMAT0000222::MIMAT0000517::MIMAT0000867::MIMAT0006224::MIMAT0006632::MIMAT0007016::MIMAT0008057::MIMAT0013049::MIMAT0013910::MIMAT0015786::MIMAT0024119 | hsa-miR-192(17)::mmu-miR-192(17)::oan-miR-192(18)::rno-miR-192(18) | CUGACCUAUGAAUUGACAGCC |
| 000493 | hsa-miR-194 | aca-miR-194-5p::age-miR-194::bta-miR-194::ccr-miR-194::cfa-miR-194::cgr-miR-194::eca-miR-194::gga-miR-194::ggo-miR-194::hsa-miR-194-5p::mml-miR-194::mmu-miR-194-5p::mne-miR-194::oan-miR-194-5p::ppy-miR-194::ptr-miR-194::rno-miR-194-5p::tgu-miR-194::xla-miR-194::xtr-miR-194 | MIMAT0000224::MIMAT0000460::MIMAT0000869::MIMAT0001133::MIMAT0002727::MIMAT0002729::MIMAT0002731::MIMAT0002733::MIMAT0002735::MIMAT0002737::MIMAT0003617::MIMAT0006681::MIMAT0006999::MIMAT0009254::MIMAT0011141::MIMAT0013050::MIMAT0014558::MIMAT0021828::MIMAT0023827::MIMAT0026252 | aca-miR-194(18)::hsa-miR-194(17)::mmu-miR-194(17)::oan-miR-194(18)::rno-miR-194(18) | UGUAACAGCAACUCCAUGUGGA |
| 000494 | hsa-miR-195 | eca-miR-195::ggo-miR-195::hsa-miR-195-5p::mml-miR-195::mmu-miR-195a-5p::ppa-miR-195::ppy-miR-195::ptr-miR-195::rno-miR-195-5p::ssc-miR-195 | MIMAT0000225::MIMAT0000461::MIMAT0000870::MIMAT0002316::MIMAT0002317::MIMAT0006228::MIMAT0008060::MIMAT0013027::MIMAT0013928::MIMAT0015790 | hsa-miR-195(17)::mmu-miR-195(17)::mmu-miR-195-5p(18)::rno-miR-195(18) | UAGCAGCACAGAAAUAUUGGC |
| 000497 | hsa-miR-197 | age-miR-197::bta-miR-197::cfa-miR-197::eca-miR-197::ggo-miR-197::hsa-miR-197-3p::mml-miR-197::ppa-miR-197::ppy-miR-197::ptr-miR-197 | MIMAT0000227::MIMAT0002600::MIMAT0002601::MIMAT0002603::MIMAT0002604::MIMAT0006230::MIMAT0006698::MIMAT0009257::MIMAT0012959::MIMAT0024180 | hsa-miR-197(17)::mmu-miR-197(14) | UUCACCACCUUCUCCACCCAGC |
| 000498 | hsa-miR-199a | ccr-miR-199-5p::dre-miR-199-5p::eca-miR-199a-5p::fru-miR-199::gga-miR-199-5p::ggo-miR-199a::hsa-miR-199a-5p::lla-miR-199a::mml-miR-199a::mml-miR-199a-5p::mmu-miR-199a-5p::mne-miR-199a::pma-miR-199a-5p::ppa-miR-199a::ppy-miR-199a::ppy-miR-199a-5p::ptr-miR-199a-5p::rno-miR-199a-5p::sla-miR-199a::ssc-miR-199a-5p::tni-miR-199::xtr-miR-199a-5p | MIMAT0000229::MIMAT0000231::MIMAT0000872::MIMAT0001152::MIMAT0001277::MIMAT0002527::MIMAT0002528::MIMAT0002529::MIMAT0002530::MIMAT0002531::MIMAT0002532::MIMAT0002533::MIMAT0002534::MIMAT0002959::MIMAT0002960::MIMAT0003618::MIMAT0006231::MIMAT0012960::MIMAT0013874::MIMAT0015792::MIMAT0019505::MIMAT0026255 | dre-miR-199(18)::dre-miR-199a(6)::gga-miR-199(18)::gga-miR-199a(8.1)::gga-miR-199a-1(5)::gga-miR-199a-2(5)::hsa-miR-199a(9.2)::mmu-miR-199a(9.2)::pma-miR-199a(18)::ptr-miR-199a(11)::rno-miR-199a(9.2)::ssc-miR-199a(18)::xtr-miR-199a(18) | CCCAGUGUUCAGACUACCUGUUC |
| 000500 | hsa-miR-199b | bta-miR-199b::eca-miR-199b-5p::hsa-miR-199b-5p::mdo-miR-199b | MIMAT0000263::MIMAT0003821::MIMAT0004125::MIMAT0013780 | hsa-miR-199b(9.2) | CCCAGUGUUUAGACUAUCUGUUC |
| 000502 | hsa-miR-200a | aca-miR-200a-3p::dre-miR-200a::eca-miR-200a::fru-miR-200a::gga-miR-200a::ggo-miR-200a::hsa-miR-200a-3p::mdo-miR-200a-3p::mml-miR-200a::mmu-miR-200a-3p::ppy-miR-200a::ptr-miR-200a::rno-miR-200a-3p::tni-miR-200a::xtr-miR-200a | MIMAT0000519::MIMAT0000682::MIMAT0000874::MIMAT0001171::MIMAT0001861::MIMAT0002981::MIMAT0002982::MIMAT0003693::MIMAT0004158::MIMAT0006233::MIMAT0008063::MIMAT0012909::MIMAT0015796::MIMAT0021845::MIMAT0024300 | aca-miR-200a(18)::hsa-miR-200a(17)::mdo-miR-200a(18)::mmu-miR-200a(17)::rno-miR-200a(18) | UAACACUGUCUGGUAACGAUGU |
| 000507 | hsa-miR-203 | cfa-miR-203::hsa-miR-203a::mml-miR-203::mmu-miR-203-3p::ppy-miR-203::ptr-miR-203::rno-miR-203a-3p | MIMAT0000236::MIMAT0000264::MIMAT0000876::MIMAT0006234::MIMAT0008065::MIMAT0009866::MIMAT0015797 | hsa-miR-203(18)::mmu-miR-203(17)::rno-miR-203(18) | GUGAAAUGUUUAGGACCACUAG |
| 000508 | hsa-miR-204 | aca-miR-204a-5p::bta-miR-204::cfa-miR-204::cgr-miR-204::dre-miR-204::eca-miR-204b::fru-miR-204a::gga-miR-204::gga-miR-211::ggo-miR-204::hsa-miR-204-5p::mdo-miR-204::mml-miR-204::mmu-miR-204-5p::mne-miR-204::oan-miR-204-5p::pma-miR-204-5p::ppa-miR-204::ppy-miR-204::ptr-miR-204::rno-miR-204-5p::sla-miR-204::ssc-miR-204::tgu-miR-204::tni-miR-204a::xtr-miR-204 | MIMAT0000237::MIMAT0000265::MIMAT0000877::MIMAT0001156::MIMAT0001279::MIMAT0002164::MIMAT0002535::MIMAT0002536::MIMAT0002537::MIMAT0002538::MIMAT0002539::MIMAT0002540::MIMAT0003101::MIMAT0003102::MIMAT0003368::MIMAT0003696::MIMAT0004127::MIMAT0004338::MIMAT0006235::MIMAT0006598::MIMAT0006841::MIMAT0013112::MIMAT0014570::MIMAT0019515::MIMAT0021853::MIMAT0023844 | aca-miR-204a(18)::fru-miR-204(18)::gga-miR-204-1(5)::gga-miR-204-2(5)::hsa-miR-204(17)::mmu-miR-204(17)::oan-miR-204(18)::pma-miR-204(18)::rno-miR-204(18) | UUCCCUUUGUCAUCCUAUGCCU |
| 000509 | hsa-miR-205 | aca-miR-205a::age-miR-205::bta-miR-205::ccr-miR-205::cfa-miR-205::dre-miR-205::eca-miR-205::fru-miR-205::gga-miR-205a::ggo-miR-205::hsa-miR-205-5p::lla-miR-205::mdo-miR-205a::mml-miR-205::mmu-miR-205-5p::mne-miR-205::ppa-miR-205::ppy-miR-205::ptr-miR-205::ssc-miR-205::tni-miR-205::xla-miR-205::xtr-miR-205a | MIMAT0000238::MIMAT0000266::MIMAT0001184::MIMAT0001280::MIMAT0002146::MIMAT0002541::MIMAT0002542::MIMAT0002543::MIMAT0002544::MIMAT0002545::MIMAT0002546::MIMAT0002965::MIMAT0002966::MIMAT0003545::MIMAT0003697::MIMAT0006236::MIMAT0009845::MIMAT0011142::MIMAT0012749::MIMAT0012962::MIMAT0015798::MIMAT0021855::MIMAT0026263 | hsa-miR-205(17)::mmu-miR-205(17)::rno-miR-205(15) | UCCUUCAUUCCACCGGAGUCUG |
| 000510 | hsa-miR-206 | bta-miR-206::ccr-miR-206::cfa-miR-206::cgr-miR-206::dre-miR-206::eca-miR-206::gga-miR-206::ggo-miR-206::hsa-miR-206::mml-miR-206::mmu-miR-206-3p::mne-miR-206::oan-miR-206-3p::pol-miR-206-3p::ppy-miR-206::ptr-miR-206::rno-miR-206-3p::xtr-miR-206 | MIMAT0000239::MIMAT0000462::MIMAT0000879::MIMAT0001139::MIMAT0001866::MIMAT0002318::MIMAT0002319::MIMAT0003623::MIMAT0006237::MIMAT0006606::MIMAT0006995::MIMAT0008066::MIMAT0009260::MIMAT0013098::MIMAT0023846::MIMAT0024181::MIMAT0025445::MIMAT0026264 | mmu-miR-206(17)::oan-miR-206(18)::rno-miR-206(18) | UGGAAUGUAAGGAAGUGUGUGG |
| 000511 | hsa-miR-208 | aca-miR-208-3p::bta-miR-208a::cfa-miR-208a::eca-miR-208a::hsa-miR-208a::mml-miR-208a::mmu-miR-208a-3p::oan-miR-208-3p::ppy-miR-208a::ptr-miR-208a | MIMAT0000241::MIMAT0000520::MIMAT0006238::MIMAT0006921::MIMAT0008067::MIMAT0009261::MIMAT0009868::MIMAT0012899::MIMAT0015799::MIMAT0021860 | aca-miR-208(18)::hsa-miR-208(10)::mmu-miR-208(10)::mmu-miR-208a(15)::oan-miR-208(18)::rno-miR-208(15) | AUAAGACGAGCAAAAAGCUUGU |
| 000512 | hsa-miR-210 | bfl-miR-210-3p::cgr-miR-210-3p::ggo-miR-210::hsa-miR-210::mml-miR-210::mmu-miR-210-3p::ptr-miR-210::rno-miR-210-3p::ssc-miR-210 | MIMAT0000267::MIMAT0000658::MIMAT0000881::MIMAT0006240::MIMAT0007761::MIMAT0008070::MIMAT0009494::MIMAT0023849::MIMAT0024146 | bfl-miR-210(18)::mmu-miR-210(17)::rno-miR-210(18) | CUGUGCGUGUGACAGCGGCUGA |
| 000514 | hsa-miR-211 | hsa-miR-211-5p::mne-miR-211::ppy-miR-211::ptr-miR-211 | MIMAT0000268::MIMAT0002548::MIMAT0002549::MIMAT0008071 | hsa-miR-211(17) | UUCCCUUUGUCAUCCUUCGCCU |
| 000515 | hsa-miR-212 | eca-miR-212::hsa-miR-212-3p::mdo-miR-212 | MIMAT0000269::MIMAT0004148::MIMAT0013030 | hsa-miR-212(17)::mmu-miR-212(9.2)::rno-miR-212(9.2) | UAACAGUCUCCAGUCACGGCC |
| 000516 | hsa-miR-213 | cgr-miR-181a-3p::dre-miR-181a-3p::fru-miR-181a-3p::gga-miR-181a-3p::ggo-miR-181a-3p::hsa-miR-181a-3p::lla-miR-181a-3p::mml-miR-181a-3p::mmu-miR-181a-1-3p::mne-miR-181a-3p::oan-miR-181a-3p::ppa-miR-181a-3p::ppy-miR-181a-3p::ptr-miR-181a-3p::rno-miR-181a-1-3p::tgu-miR-181a-3p::tni-miR-181a-3p | MIMAT0000270::MIMAT0000660::MIMAT0000884::MIMAT0001150::MIMAT0001282::MIMAT0002622::MIMAT0002624::MIMAT0002627::MIMAT0002629::MIMAT0002632::MIMAT0002634::MIMAT0002636::MIMAT0003005::MIMAT0003006::MIMAT0006834::MIMAT0014641::MIMAT0023798 | dre-miR-181a*(18)::fru-miR-181a*(18)::fru-miR-213(8)::gga-miR-181a*(18)::gga-miR-213(8)::ggo-miR-181a*(18)::ggo-miR-213(8)::hsa-miR-181a*(17)::hsa-miR-213(8)::lla-miR-181a*(18)::lla-miR-213(8)::mml-miR-181a*(18)::mml-miR-213(8)::mmu-miR-181a*(9.2)::mmu-miR-181a-1*(17)::mmu-miR-213(8)::mne-miR-181a*(18)::mne-miR-213(8)::oan-miR-181a*(18)::ppa-miR-181a*(18)::ppa-miR-213(8)::ppy-miR-181a*(18)::ppy-miR-213(8)::ptr-miR-181a*(18)::ptr-miR-213(8)::rno-miR-181a*(15)::rno-miR-181a-1*(18)::rno-miR-213(8)::tgu-miR-181a*(18)::tni-miR-181a*(18)::tni-miR-213(8) | ACCAUCGACCGUUGAUUGUACC |
| 000518 | hsa-miR-215 | eca-miR-215::gga-miR-215::ggo-miR-215::hsa-miR-215::mml-miR-215::mne-miR-215::oan-miR-215-5p::ppy-miR-215::ptr-miR-215::ssc-miR-215::tgu-miR-215 | MIMAT0000272::MIMAT0001134::MIMAT0002728::MIMAT0002730::MIMAT0002732::MIMAT0002734::MIMAT0002736::MIMAT0007000::MIMAT0010192::MIMAT0013190::MIMAT0014577 | oan-miR-215(18) | AUGACCUAUGAAUUGACAGAC |
| 000521 | hsa-miR-218 | age-miR-218::cfa-miR-218::eca-miR-218::gga-miR-218::ggo-miR-218::hsa-miR-218-5p::lca-miR-218::lla-miR-218::mdo-miR-218::mml-miR-218::mmu-miR-218-5p::mne-miR-218::oan-miR-218-5p::ola-miR-218a::ppa-miR-218::ppy-miR-218::ptr-miR-218::rno-miR-218a-5p::sla-miR-218::ssc-miR-218::ssc-miR-218-5p::tgu-miR-218::xtr-miR-218 | MIMAT0000275::MIMAT0000663::MIMAT0000888::MIMAT0001144::MIMAT0002565::MIMAT0002566::MIMAT0002567::MIMAT0002568::MIMAT0002569::MIMAT0002570::MIMAT0002571::MIMAT0002572::MIMAT0002573::MIMAT0002574::MIMAT0003631::MIMAT0004133::MIMAT0006672::MIMAT0006815::MIMAT0012928::MIMAT0014522::MIMAT0022529::MIMAT0022961::MIMAT0025371 | bta-miR-218(13)::gga-miR-218-1(5)::gga-miR-218-2(5)::gga-miR-218-3(5)::hsa-miR-218(17)::mmu-miR-218(17)::oan-miR-218(18)::rno-miR-218(16)::rno-miR-218a(18) | UUGUGCUUGAUCUAACCAUGU |
| 000522 | hsa-miR-219 | bfl-miR-219::cfa-miR-219-5p::cte-miR-219::eca-miR-219-5p::gga-miR-219::ggo-miR-219::hsa-miR-219-5p::mdo-miR-219::mml-miR-219::mml-miR-219-5p::mmu-miR-219-5p::ppy-miR-219::ppy-miR-219-5p::ptr-miR-219-5p::rno-miR-219-5p::tca-miR-219-5p::xtr-miR-219 | MIMAT0000276::MIMAT0000664::MIMAT0000889::MIMAT0001170::MIMAT0002575::MIMAT0002576::MIMAT0002577::MIMAT0003632::MIMAT0004134::MIMAT0006245::MIMAT0006611::MIMAT0008377::MIMAT0009193::MIMAT0009556::MIMAT0010012::MIMAT0013099::MIMAT0015804 | cap-miR-219(14)::cfa-miR-219(18)::dre-miR-219(6)::hsa-miR-219(9.2)::mmu-miR-219(15)::rno-miR-219(9.2) | UGAUUGUCCAAACGCAAUUCU |
| 000523 | hsa-miR-220 |  |  | hsa-miR-220(10)::hsa-miR-220a(15)::ppa-miR-220(15)::ptr-miR-220(11)::ptr-miR-220a(15) | CCACACCGUAUCUGACACUUU |
| 000524 | hsa-miR-221 | cgr-miR-221-3p::dre-miR-221::eca-miR-221::fru-miR-221::gga-miR-221::ggo-miR-221::hsa-miR-221-3p::mdo-miR-221::mml-miR-221::mmu-miR-221-3p::ola-miR-221::ppa-miR-221::ppy-miR-221::ptr-miR-221::rno-miR-221-3p::tgu-miR-221::tni-miR-221::xtr-miR-221 | MIMAT0000278::MIMAT0000669::MIMAT0000890::MIMAT0001108::MIMAT0001288::MIMAT0002583::MIMAT0002584::MIMAT0002585::MIMAT0002586::MIMAT0002906::MIMAT0002907::MIMAT0003700::MIMAT0004160::MIMAT0008076::MIMAT0013203::MIMAT0014512::MIMAT0022530::MIMAT0023859 | hsa-miR-221(17)::mmu-miR-221(17)::rno-miR-221(18) | AGCUACAUUGUCUGCUGGGUUUC |
| 000527 | hsa-miR-296 | hsa-miR-296-5p::mml-miR-296-5p::mmu-miR-296-5p::ppy-miR-296-5p::rno-miR-296-5p | MIMAT0000374::MIMAT0000690::MIMAT0000898::MIMAT0006251::MIMAT0015809 | hsa-miR-296(9.2)::mmu-miR-296(9.2)::rno-miR-296(9.2)::rno-miR-296*(18) | AGGGCCCCCCCUCAAUCCUGU |
| 000528 | hsa-miR-301 | aca-miR-301a-3p::ccr-miR-301a::cfa-miR-301a::cgr-miR-301a-3p::eca-miR-301a::hsa-miR-301a-3p::mdo-miR-301::mml-miR-301a::mmu-miR-301a-3p::oan-miR-301-3p::ptr-miR-301a::rno-miR-301a-3p::tgu-miR-301-3p::xtr-miR-301 | MIMAT0000379::MIMAT0000552::MIMAT0000688::MIMAT0003635::MIMAT0006257::MIMAT0006910::MIMAT0008084::MIMAT0009853::MIMAT0012739::MIMAT0013032::MIMAT0014595::MIMAT0021916::MIMAT0023891::MIMAT0026288 | aca-miR-301a(18)::hsa-miR-301(9.2)::hsa-miR-301a(17)::mmu-miR-301(9.2)::mmu-miR-301a(17)::oan-miR-301(18)::rno-miR-301a(18)::tgu-miR-301(18) | CAGUGCAAUAGUAUUGUCAAAGC |
| 000529 | hsa-miR-302a | hsa-miR-302a-3p::mml-miR-302a::mmu-miR-302a-3p::ptr-miR-302a | MIMAT0000380::MIMAT0000684::MIMAT0006259::MIMAT0008086 | hsa-miR-302(3.1)::hsa-miR-302a(17)::mmu-miR-302(9.2)::mmu-miR-302a(17) | UAAGUGCUUCCAUGUUUUGGUGA |
| 000531 | hsa-miR-302b | aca-miR-302::bta-miR-302b::eca-miR-302b::gga-miR-302b-3p::hsa-miR-302b-3p::mml-miR-302b::mmu-miR-302b-3p::ppy-miR-302b::ptr-miR-302b | MIMAT0000715::MIMAT0003357::MIMAT0003374::MIMAT0006260::MIMAT0008087::MIMAT0009280::MIMAT0012912::MIMAT0015817::MIMAT0021919 | gga-miR-302b(18)::hsa-miR-302b(17)::mmu-miR-302b(17) | UAAGUGCUUCCAUGUUUUAGUAG |
| 000533 | hsa-miR-302c | bta-miR-302c::eca-miR-302c::gga-miR-302c-3p::hsa-miR-302c-3p::mml-miR-302c::ppy-miR-302c::ptr-miR-302c | MIMAT0000717::MIMAT0003359::MIMAT0006261::MIMAT0008088::MIMAT0009281::MIMAT0012913::MIMAT0015818 | gga-miR-302c(18)::hsa-miR-302c(17) | UAAGUGCUUCCAUGUUUCAGUGG |
| 000534 | hsa-miR-302c# | cfa-miR-302c::hsa-miR-302c-5p | MIMAT0000716::MIMAT0009857 | hsa-miR-302c*(17) | UUUAACAUGGGGGUACCUGCUG |
| 000535 | hsa-miR-302d | hsa-miR-302d-3p::mml-miR-302d::mmu-miR-302d-3p::ppy-miR-302d::ptr-miR-302d | MIMAT0000718::MIMAT0003377::MIMAT0006262::MIMAT0008089::MIMAT0015819 | hsa-miR-302d(17)::mmu-miR-302d(17) | UAAGUGCUUCCAUGUUUGAGUGU |
| 000539 | hsa-miR-324-5p | bta-miR-324::cfa-miR-324::eca-miR-324-5p::hsa-miR-324-5p::mml-miR-324-5p::mmu-miR-324-5p::ppy-miR-324-5p::rno-miR-324-5p::ssc-miR-324 | MIMAT0000553::MIMAT0000555::MIMAT0000761::MIMAT0006266::MIMAT0009285::MIMAT0009896::MIMAT0013033::MIMAT0013957::MIMAT0015827 | | CGCAUCCCCUAGGGCAUUGGUGU |
| 000540 | hsa-miR-325 | hsa-miR-325::mml-miR-325::ppy-miR-325 | MIMAT0000771::MIMAT0006268::MIMAT0015829 | | CCUAGUAGGUGUCCAGUAAGUGU |
| 000542 | hsa-miR-326 | bta-miR-326::cfa-miR-326::ggo-miR-326::hsa-miR-326::ppy-miR-326::ptr-miR-326::ssc-miR-326 | MIMAT0000756::MIMAT0002140::MIMAT0008099::MIMAT0009286::MIMAT0009894::MIMAT0015830::MIMAT0024215 | | CCUCUGGGCCCUUCCUCCAG |
| 000543 | hsa-miR-328 | bta-miR-328::cfa-miR-328::cgr-miR-328::eca-miR-328::hsa-miR-328::mmu-miR-328-3p::ppy-miR-328::ptr-miR-328::rno-miR-328a-3p::ssc-miR-328 | MIMAT0000564::MIMAT0000565::MIMAT0000752::MIMAT0006688::MIMAT0008100::MIMAT0009287::MIMAT0012929::MIMAT0013949::MIMAT0015831::MIMAT0023914 | mmu-miR-328(17)::rno-miR-328(15)::rno-miR-328a(18) | CUGGCCCUCUCUGCCCUUCCGU |
| 000544 | hsa-miR-330 | bta-miR-330::ggo-miR-330::hsa-miR-330-3p::mml-miR-330-3p::ppy-miR-330-3p::ptr-miR-330 | MIMAT0000751::MIMAT0006271::MIMAT0008102::MIMAT0009290::MIMAT0015834::MIMAT0024088 | hsa-miR-330(9.2) | GCAAAGCACACGGCCUGCAGAGA |
| 000545 | hsa-miR-331 | bta-miR-331::cfa-miR-331::eca-miR-331::hsa-miR-331-3p::mml-miR-331-3p::mmu-miR-331-3p::ptr-miR-331::rno-miR-331-3p::ssc-miR-331-3p | MIMAT0000570::MIMAT0000571::MIMAT0000760::MIMAT0004339::MIMAT0006273::MIMAT0008103::MIMAT0009895::MIMAT0013188::MIMAT0013930 | hsa-miR-331(9.2)::mmu-miR-331(9.2)::rno-miR-331(18) | GCCCCUGGGCCUAUCCUAGAA |
| 000546 | hsa-miR-335 | bta-miR-335::cfa-miR-335::eca-miR-335::hsa-miR-335-5p::mml-miR-335::mmu-miR-335-5p::ppy-miR-335::ptr-miR-335::rno-miR-335 | MIMAT0000575::MIMAT0000765::MIMAT0000766::MIMAT0006274::MIMAT0006624::MIMAT0008104::MIMAT0009291::MIMAT0012942::MIMAT0015837 | hsa-miR-335(17)::mmu-miR-335(9.2) | UCAAGAGCAAUAACGAAAAAUGU |
| 000553 | hsa-miR-346 | bta-miR-346::cfa-miR-346::eca-miR-346::hsa-miR-346::mml-miR-346::ppy-miR-346::ptr-miR-346 | MIMAT0000773::MIMAT0006285::MIMAT0008112::MIMAT0009297::MIMAT0009898::MIMAT0012902::MIMAT0015847 | | UGUCUGCCCGCAUGCCUGCCUCU |
| 000554 | hsa-miR-361 | bta-miR-361::cfa-miR-361::cgr-miR-361::eca-miR-361-5p::ggo-miR-361::hsa-miR-361-5p::mml-miR-361-5p::mmu-miR-361-5p::ppy-miR-361-5p::rno-miR-361-5p::ssc-miR-361-5p | MIMAT0000703::MIMAT0000704::MIMAT0003117::MIMAT0003830::MIMAT0006286::MIMAT0006751::MIMAT0013208::MIMAT0013933::MIMAT0015848::MIMAT0023933::MIMAT0024136 | hsa-miR-361(9.2)::mmu-miR-361(17)::rno-miR-361(18) | UUAUCAGAAUCUCCAGGGGUAC |
| 000555 | hsa-miR-367 | aca-miR-367::eca-miR-367::hsa-miR-367-3p::mml-miR-367::mmu-miR-367-3p::ppy-miR-367::ptr-miR-367::tgu-miR-367 | MIMAT0000719::MIMAT0003181::MIMAT0006292::MIMAT0008118::MIMAT0012918::MIMAT0015854::MIMAT0021949::MIMAT0025395 | hsa-miR-367(17)::mmu-miR-367(17) | AAUUGCACUUUAGCAAUGGUGA |
| 000557 | hsa-miR-369-3p | bta-miR-369-3p::cgr-miR-369-3p::eca-miR-369-3p::hsa-miR-369-3p::mml-miR-369-3p::mmu-miR-369-3p::oar-miR-369-3p::ppy-miR-369-3p::ptr-miR-369::rno-miR-369-3p::ssc-miR-369 | MIMAT0000721::MIMAT0003186::MIMAT0003207::MIMAT0003802::MIMAT0006294::MIMAT0008119::MIMAT0013141::MIMAT0015714::MIMAT0015856::MIMAT0019332::MIMAT0023938 | hsa-miR-369(6) | AAUAAUACAUGGUUGAUCUUU |
| 000560 | hsa-miR-372 | hsa-miR-372::mml-miR-372::ppy-miR-372::ptr-miR-372 | MIMAT0000724::MIMAT0006298::MIMAT0008122::MIMAT0015860 | | AAAGUGCUGCGACAUUUGAGCGU |
| 000561 | hsa-miR-373 | hsa-miR-373-3p::mml-miR-373::ppy-miR-373 | MIMAT0000726::MIMAT0006299::MIMAT0015861 | hsa-miR-373(17) | GAAGUGCUUCGAUUUUGGGGUGU |
| 000563 | hsa-miR-374 | bta-miR-374a::eca-miR-374a::hsa-miR-374a-5p::mml-miR-374a::ppy-miR-374a::ptr-miR-374a::ssc-miR-374a-5p | MIMAT0000727::MIMAT0004342::MIMAT0006300::MIMAT0008123::MIMAT0013213::MIMAT0013913::MIMAT0015862 | bta-miR-374(11)::hsa-miR-374(9.2)::hsa-miR-374a(17)::ssc-miR-374a(18) | UUAUAAUACAACCUGAUAAGUG |
| 000564 | hsa-miR-375 | cfa-miR-375::ggo-miR-375::hsa-miR-375::mml-miR-375::mmu-miR-375-3p::ppy-miR-375::ptr-miR-375::rno-miR-375-3p | MIMAT0000728::MIMAT0000739::MIMAT0005307::MIMAT0006302::MIMAT0008125::MIMAT0009871::MIMAT0015863::MIMAT0024189 | mmu-miR-375(17)::rno-miR-375(18) | UUUGUUCGUUCGGCUCGCGUGA |
| 000565 | hsa-miR-376a | bta-miR-376a::cfa-miR-376a::eca-miR-376a::hsa-miR-376a-3p::mml-miR-376a::oar-miR-376a-3p::ppy-miR-376a::ptr-miR-376a::ssc-miR-376a-3p | MIMAT0000729::MIMAT0006303::MIMAT0006722::MIMAT0008126::MIMAT0009946::MIMAT0013143::MIMAT0015716::MIMAT0015864::MIMAT0019287 | cfa-miR-376(12)::hsa-miR-376a(17)::ssc-miR-376a(18) | AUCAUAGAGGAAAAUCCACGU |
| 000566 | hsa-miR-377 | bta-miR-377::eca-miR-377::hsa-miR-377-3p::mml-miR-377::mmu-miR-377-3p::oar-miR-377-3p::ppy-miR-377::ptr-miR-377 | MIMAT0000730::MIMAT0000741::MIMAT0006306::MIMAT0008129::MIMAT0009304::MIMAT0013146::MIMAT0015867::MIMAT0019322 | hsa-miR-377(17)::mmu-miR-377(17) | AUCACACAAAGGCAACUUUUGU |
| 000567 | hsa-miR-378 | cgr-miR-378-5p::hsa-miR-378a-5p::mmu-miR-378a-5p::rno-miR-378a-5p | MIMAT0000731::MIMAT0000742::MIMAT0003378::MIMAT0023943 | hsa-miR-378(9.2)::hsa-miR-378*(17)::mmu-miR-378(9.2)::mmu-miR-378*(17)::mmu-miR-378-5p(18)::rno-miR-378(9.2)::rno-miR-378*(18) | CUCCUGACUCCAGGUCCUGUGU |
| 000569 | hsa-miR-380-3p | hsa-miR-380-3p::ptr-miR-380 | MIMAT0000735::MIMAT0008132 | hsa-miR-380(17) | UAUGUAAUAUGGUCCACAUCUU |
| 000570 | hsa-miR-380-5p | bta-miR-380-5p::hsa-miR-380-5p | MIMAT0000734::MIMAT0003803 | hsa-miR-380*(17)::mmu-miR-380-5p(9.2) | UGGUUGACCAUAGAACAUGCGC |
| 000571 | hsa-miR-381 | bta-miR-381::cfa-miR-381::cgr-miR-381::eca-miR-381::hsa-miR-381-3p::mml-miR-381::mmu-miR-381-3p::ppy-miR-381::ptr-miR-381 | MIMAT0000736::MIMAT0000746::MIMAT0006310::MIMAT0008133::MIMAT0009307::MIMAT0009891::MIMAT0013149::MIMAT0015871::MIMAT0023946 | hsa-miR-381(18)::mmu-miR-381(17) | UAUACAAGGGCAAGCUCUCUGU |
| 000572 | hsa-miR-382 | bta-miR-382::cgr-miR-382::eca-miR-382::hsa-miR-382-5p::mml-miR-382::mmu-miR-382-5p::oar-miR-382-5p::ptr-miR-382::rno-miR-382-5p | MIMAT0000737::MIMAT0000747::MIMAT0003201::MIMAT0006311::MIMAT0008134::MIMAT0009308::MIMAT0013150::MIMAT0019306::MIMAT0023947 | hsa-miR-382(17)::mmu-miR-382(17)::rno-miR-382(18) | GAAGUUGUUCGUGGUGGAUUCG |
| 000573 | hsa-miR-383 | bta-miR-383::cfa-miR-383::eca-miR-383::gga-miR-383::hsa-miR-383::mdo-miR-383::mml-miR-383::ppy-miR-383::ptr-miR-383::tgu-miR-383::xtr-miR-383 | MIMAT0000738::MIMAT0003363::MIMAT0003639::MIMAT0004139::MIMAT0006312::MIMAT0006629::MIMAT0008135::MIMAT0009309::MIMAT0013185::MIMAT0014531::MIMAT0015873 | | AGAUCAGAAGGUGAUUGUGGCU |
| 000574 | hsa-miR-384 | hsa-miR-384::mml-miR-384 | MIMAT0001075::MIMAT0006313 | | AUUCCUAGAAAUUGUUCAUA |
| 000577 | hsa-miR-98 | age-miR-98::bta-miR-98::cfa-miR-98::cgr-miR-98::eca-miR-98::ggo-miR-98::hsa-miR-98-5p::mml-miR-98::mmu-miR-98-5p::oan-miR-98::ppa-miR-98::ppy-miR-98::ptr-miR-98::rno-miR-98-5p::ssc-miR-98::xtr-miR-98 | MIMAT0000096::MIMAT0000545::MIMAT0000819::MIMAT0002404::MIMAT0002405::MIMAT0002406::MIMAT0002407::MIMAT0002408::MIMAT0002409::MIMAT0003581::MIMAT0003809::MIMAT0006756::MIMAT0007178::MIMAT0013247::MIMAT0013905::MIMAT0024015 | hsa-miR-98(18)::mmu-miR-98(17)::rno-miR-98(18) | UGAGGUAGUAAGUUGUAUUGUU |
| 000580 | hsa-miR-20a | bta-miR-20a::ccr-miR-20a-5p::cfa-miR-20a::cgr-miR-20a::dre-miR-20a-5p::eca-miR-20a::fru-miR-20::gga-miR-20a::hsa-miR-20a-5p::mdo-miR-20::mmu-miR-20a-5p::rno-miR-20a-5p::tgu-miR-20a::tni-miR-20::xtr-miR-20a-5p | MIMAT0000075::MIMAT0000529::MIMAT0000602::MIMAT0001111::MIMAT0001786::MIMAT0003083::MIMAT0003084::MIMAT0003527::MIMAT0003669::MIMAT0004169::MIMAT0006651::MIMAT0013088::MIMAT0014553::MIMAT0023847::MIMAT0026265 | cfa-miR-20(12)::dre-miR-20a(18)::gga-miR-20(5.1)::hsa-miR-20(6)::hsa-miR-20a(17)::mmu-miR-20(7.1)::mmu-miR-20a(17)::rno-miR-20(7.1)::rno-miR-20a(18)::xtr-miR-20a(18) | UAAAGUGCUUAUAGUGCAGGUAG |
| 000583 | hsa-miR-9 | aae-miR-9a::aga-miR-9a::age-miR-9::ame-miR-9a::api-miR-9a::asu-miR-9-5p::bmo-miR-9a-5p::cfa-miR-9::cte-miR-9-5p::dan-miR-9a::der-miR-9a::dgr-miR-9a::dme-miR-9a-5p::dmo-miR-9a::dpe-miR-9a::dps-miR-9a::dpu-miR-9-5p::dre-miR-9-5p::dse-miR-9a::dsi-miR-9a::dvi-miR-9a::dwi-miR-9a::dya-miR-9a::eca-miR-9a::fru-miR-9::gga-miR-9-5p::ggo-miR-9::hco-miR-9::hsa-miR-9-5p::lgi-miR-9::lla-miR-9::lmi-miR-9a-5p::mdo-miR-9-5p::mml-miR-9::mmu-miR-9-5p::mne-miR-9::ngi-miR-9a::nlo-miR-9a::nvi-miR-9a::ola-miR-9a-5p::ola-miR-9b-5p::pol-miR-9b-5p::ppy-miR-9::ptr-miR-9::rno-miR-9a-5p::ssc-miR-9-1::ssc-miR-9-2::tgu-miR-9-5p::tni-miR-9::xtr-miR-9a-5p | MIMAT0000114::MIMAT0000142::MIMAT0000441::MIMAT0000781::MIMAT0001195::MIMAT0001211::MIMAT0001491::MIMAT0001526::MIMAT0001769::MIMAT0002168::MIMAT0002169::MIMAT0002275::MIMAT0002276::MIMAT0002277::MIMAT0002278::MIMAT0002279::MIMAT0002910::MIMAT0002911::MIMAT0003553::MIMAT0004087::MIMAT0004194::MIMAT0006160::MIMAT0006674::MIMAT0008458::MIMAT0008505::MIMAT0008589::MIMAT0008670::MIMAT0008717::MIMAT0008800::MIMAT0008843::MIMAT0008929::MIMAT0009034::MIMAT0009070::MIMAT0009508::MIMAT0009562::MIMAT0010146::MIMAT0012671::MIMAT0012967::MIMAT0014323::MIMAT0014502::MIMAT0014748::MIMAT0015697::MIMAT0015729::MIMAT0018405::MIMAT0018442::MIMAT0021431::MIMAT0022546::MIMAT0022636::MIMAT0023348::MIMAT0025420 | asu-miR-9(18)::bmo-miR-9(14)::bmo-miR-9a(18)::bta-miR-9(13)::cap-miR-9(14)::cte-miR-9(18)::dme-miR-9a(16)::dpu-miR-9(18)::dre-miR-9(18)::gga-miR-9(18)::hsa-miR-9(17)::lmi-miR-9a(18)::mdo-miR-9(18)::mmu-miR-9(17)::rno-miR-9(18)::tca-miR-9a(16)::tgu-miR-9(18)::xtr-miR-9a(18) | UCUUUGGUUAUCUAGCUGUAUGA |
| 000587 | hsa-miR-29c | bta-miR-29c::ccr-miR-29a::cfa-miR-29c::dre-miR-29a::eca-miR-29c::fru-miR-29a::ggo-miR-29c::hsa-miR-29c-3p::mml-miR-29c::mmu-miR-29c-3p::ppy-miR-29c::ptr-miR-29c::rno-miR-29c-3p::ssc-miR-29c::tni-miR-29a | MIMAT0000536::MIMAT0000681::MIMAT0000803::MIMAT0001802::MIMAT0002166::MIMAT0003053::MIMAT0003054::MIMAT0003829::MIMAT0006169::MIMAT0006705::MIMAT0008082::MIMAT0012964::MIMAT0015735::MIMAT0024084::MIMAT0026286 | gga-miR-29c(5)::hsa-miR-29c(17)::mmu-miR-29c(17)::rno-miR-29c(18) | UAGCACCAUUUGAAAUCGGUUA |
| 000590 | hsa-miR-129 | aca-miR-129a-5p::ccr-miR-129::cfa-miR-129::cgr-miR-129::eca-miR-129a-5p::eca-miR-129b-5p::hsa-miR-129-5p::mml-miR-129::mml-miR-129-5p::mmu-miR-129-5p::oan-miR-129-5p::ppy-miR-129-5p::ptr-miR-129::rno-miR-129-5p::sha-miR-129::ssc-miR-129b::tgu-miR-129-5p::xtr-miR-129 | MIMAT0000209::MIMAT0000242::MIMAT0000600::MIMAT0003590::MIMAT0006185::MIMAT0006623::MIMAT0006801::MIMAT0008010::MIMAT0012775::MIMAT0012932::MIMAT0013045::MIMAT0014625::MIMAT0015751::MIMAT0020586::MIMAT0021734::MIMAT0022827::MIMAT0023751::MIMAT0026209 | aca-miR-129a(18)::hsa-miR-129(9.2)::oan-miR-129(18)::ppy-miR-129(18)::rno-miR-129(18)::tgu-miR-129*(18) | CUUUUUGCGGUCUGGGCUUGC |
| 000592 | hsa-miR-136 | bta-miR-136::cfa-miR-136::ggo-miR-136::hsa-miR-136-5p::mml-miR-136::oar-miR-136::ppa-miR-136::ppy-miR-136::ptr-miR-136::rno-miR-136-5p::ssc-miR-136 | MIMAT0000448::MIMAT0000842::MIMAT0001417::MIMAT0002158::MIMAT0002292::MIMAT0002293::MIMAT0002294::MIMAT0002295::MIMAT0006192::MIMAT0006714::MIMAT0009230 | hsa-miR-136(17)::mmu-miR-136(9.2)::rno-miR-136(18) | ACUCCAUUUGUUUUGAUGAUGGA |
| 000600 | hsa-miR-299-5p | bta-miR-299::cfa-miR-299::hsa-miR-299-5p::mml-miR-299-5p::mmu-miR-299a-5p::oar-miR-299-5p::ppy-miR-299-5p::ptr-miR-299::rno-miR-299a-5p | MIMAT0000377::MIMAT0000687::MIMAT0002890::MIMAT0000901::MIMAT0006255::MIMAT0009274::MIMAT0009885::MIMAT0012801::MIMAT0015813::MIMAT0019251 | hsa-miR-299(6)::mmu-miR-299(9.2)::mmu-miR-299*(17)::mmu-miR-299-5p(18)::rno-miR-299(18) | UGGUUUACCGUCCCACAUACAU |
| 000602 | hsa-miR-30b | aca-miR-30b-5p::bta-miR-30b-5p::ccr-miR-30b::cfa-miR-30b::cgr-miR-30b-5p::dre-miR-30b::eca-miR-30b::fru-miR-30b::gga-miR-30b::hsa-miR-30b-5p::mmu-miR-30b-5p::oan-miR-30b-5p::ppy-miR-30b::rno-miR-30b-5p::ssc-miR-30b-5p::tgu-miR-30e::tni-miR-30b::xtr-miR-30b | MIMAT0000130::MIMAT0000420::MIMAT0000806::MIMAT0001130::MIMAT0001804::MIMAT0002947::MIMAT0002948::MIMAT0003547::MIMAT0003576::MIMAT0006617::MIMAT0007043::MIMAT0007756::MIMAT0013005::MIMAT0015736::MIMAT0021922::MIMAT0023896::MIMAT0025411::MIMAT0026289 | aca-miR-30b(18)::bta-miR-30b(13)::hsa-miR-30b(17)::mmu-miR-30b(17)::oan-miR-30b(18)::rno-miR-30b(9.2)::ssc-miR-30b(14) | UGUAAACAUCCUACACUCAGCU |
| 000604 | hsa-miR-424 | eca-miR-424::hsa-miR-424-5p::mml-miR-424::ppy-miR-424::ptr-miR-424::ssc-miR-424-5p | MIMAT0001341::MIMAT0006323::MIMAT0008143::MIMAT0013217::MIMAT0013920::MIMAT0015884 | eca-miR-322(14)::hsa-miR-424(17)::ssc-miR-424(18) | CAGCAGCAAUUCAUGUUUUGAA |
| 001006 | RNU48 | RNU48 | NR_002745 | U48 | GAUGACCCCAGGUAACUCUGAGUGUGUCGCUGAUGCCAUCACCGCAGCGCUCUGACC |
| 001011 | hsa-miR-200a# | cfa-miR-200a::hsa-miR-200a-5p::mmu-miR-200a-5p | MIMAT0001620::MIMAT0004619::MIMAT0009865 | hsa-miR-200a*(17)::mmu-miR-200a*(17) | CAUCUUACCGGACAGUGCUGGA |
| 001014 | hsa-miR-20b | eca-miR-20b::gga-miR-20b::ggo-miR-20b::hsa-miR-20b-5p::mml-miR-20b::mmu-miR-20b-5p::oan-miR-20b-5p::ppy-miR-20b::ptr-miR-20b::rno-miR-20b-5p::tgu-miR-20b::xla-miR-20::xtr-miR-20b | MIMAT0001348::MIMAT0001411::MIMAT0001413::MIMAT0003187::MIMAT0003211::MIMAT0003707::MIMAT0006164::MIMAT0006853::MIMAT0008069::MIMAT0013202::MIMAT0014591::MIMAT0015732::MIMAT0024227 | hsa-miR-20b(17)::mmu-miR-20b(17)::oan-miR-20b(18) | CAAAGUGCUCAUAGUGCAGGUAG |
| 001015 | hsa-miR-299-3p | eca-miR-299::hsa-miR-299-3p | MIMAT0000687::MIMAT0013130 | | UAUGUGGGAUGGUAAACCGCUU |
| 001020 | hsa-miR-365 | aca-miR-365-3p::bta-miR-365-3p::ccr-miR-365::cfa-miR-365::cgr-miR-365-3p::dre-miR-365::eca-miR-365::fru-miR-365::gga-miR-365::hsa-miR-365a-3p::hsa-miR-365b-3p::mdo-miR-365::mml-miR-365::mmu-miR-365-3p::oan-miR-365-3p::ppy-miR-365::ptr-miR-365::rno-miR-365-3p::ssc-miR-365-3p::tgu-miR-365::tni-miR-365::xtr-miR-365 | MIMAT0000710::MIMAT0000711::MIMAT0001540::MIMAT0001549::MIMAT0001875::MIMAT0002967::MIMAT0002968::MIMAT0003361::MIMAT0003637::MIMAT0004137::MIMAT0004341::MIMAT0006291::MIMAT0006823::MIMAT0008117::MIMAT0013038::MIMAT0013904::MIMAT0014582::MIMAT0015853::MIMAT0021948::MIMAT0022834::MIMAT0023936::MIMAT0026294 | aca-miR-365(18)::bta-miR-365(13)::hsa-miR-365(17)::mmu-miR-365(17)::oan-miR-365(18)::rno-miR-365(18)::ssc-miR-365(15) | UAAUGCCCCUAAAAAUCCUUAU |
| 001021 | hsa-miR-369-5p | cgr-miR-369-5p::hsa-miR-369-5p::mml-miR-369-5p::mmu-miR-369-5p::ppy-miR-369-5p::rno-miR-369-5p | MIMAT0001621::MIMAT0003185::MIMAT0003206::MIMAT0006293::MIMAT0015855::MIMAT0023937 | | AGAUCGACCGUGUUAUAUUCGC |
| 001023 | hsa-miR-412 | hsa-miR-412::mml-miR-412::ppy-miR-412::ptr-miR-412 | MIMAT0002170::MIMAT0006318::MIMAT0008139::MIMAT0015879 | mmu-miR-412(9.2)::rno-miR-412(15) | ACUUCACCUGGUCCACUAGCCGU |
| 001024 | hsa-miR-429 | hsa-miR-429::mml-miR-429::ppy-miR-429 | MIMAT0001536::MIMAT0006325::MIMAT0015886 | | UAAUACUGUCUGGUAAAACCGU |
| 001026 | hsa-miR-432 | bta-miR-432::cfa-miR-432::eca-miR-432::hsa-miR-432-5p::mml-miR-432::oar-miR-432::ppy-miR-432::ptr-miR-432 | MIMAT0001416::MIMAT0002814::MIMAT0006327::MIMAT0008146::MIMAT0009317::MIMAT0009904::MIMAT0013157::MIMAT0015888 | hsa-miR-432(17) | UCUUGGAGUAGGUCAUUGGGUGG |
| 001027 | hsa-miR-432# | hsa-miR-432-3p | MIMAT0002815 | hsa-miR-432*(17) | CUGGAUGGCUCCUCCAUGUCU |
| 001028 | hsa-miR-433 | bta-miR-433::cfa-miR-433::eca-miR-433::hsa-miR-433::mml-miR-433::mmu-miR-433-3p::oar-miR-433-3p::ppy-miR-433::ptr-miR-433::rno-miR-433-3p | MIMAT0001420::MIMAT0001627::MIMAT0001628::MIMAT0006328::MIMAT0006712::MIMAT0008147::MIMAT0009318::MIMAT0013158::MIMAT0015889::MIMAT0019242 | mmu-miR-433(17)::rno-miR-433(18) | AUCAUGAUGGGCUCCUCGGUGU |
| 001029 | hsa-miR-448 | bta-miR-448::cfa-miR-448::eca-miR-448::hsa-miR-448::mml-miR-448::mmu-miR-448-3p::ppy-miR-448::ptr-miR-448 | MIMAT0001532::MIMAT0001533::MIMAT0001535::MIMAT0006329::MIMAT0008148::MIMAT0009319::MIMAT0013218::MIMAT0015890 | mmu-miR-448(15)::rno-miR-448(15) | UUGCAUAUGUAGGAUGUCCCAU |
| 001030 | hsa-miR-449 | bta-miR-449a::cfa-miR-449::eca-miR-449a::hsa-miR-449a::mdo-miR-449::mml-miR-449a::mmu-miR-449a-5p::ppy-miR-449a::rno-miR-449a-5p | MIMAT0001541::MIMAT0001542::MIMAT0001543::MIMAT0001544::MIMAT0004140::MIMAT0006330::MIMAT0009320::MIMAT0013102::MIMAT0015891 | hsa-miR-449(9.2)::mmu-miR-449(9.2)::mmu-miR-449a(17)::rno-miR-449(9.2)::rno-miR-449a(18) | UGGCAGUGUAUUGUUAGCUGGU |
| 001036 | hsa-miR-485-5p | eca-miR-485-5p::hsa-miR-485-5p::mml-miR-485-5p::mmu-miR-485-5p::ppy-miR-485-5p::rno-miR-485-5p | MIMAT0002175::MIMAT0003128::MIMAT0003203::MIMAT0006342::MIMAT0013159::MIMAT0015902 | mmu-miR-485(17)::rno-miR-485(18) | AGAGGCUGGCCGUGAUGAAUUC |
| 001037 | hsa-miR-490 | bta-miR-490::cfa-miR-490::eca-miR-490-3p::gga-miR-490::ggo-miR-490::hsa-miR-490-3p::mml-miR-490-3p::mmu-miR-490-3p::ppy-miR-490-3p::ptr-miR-490::rno-miR-490-3p::ssc-miR-490::ssc-miR-490-3p | MIMAT0002806::MIMAT0003366::MIMAT0003780::MIMAT0006351::MIMAT0008165::MIMAT0009331::MIMAT0009875::MIMAT0012823::MIMAT0012945::MIMAT0015910::MIMAT0022960::MIMAT0024205::MIMAT0025376 | hsa-miR-490(9.2)::mmu-miR-490(15)::rno-miR-490(18) | CAACCUGGAGGACUCCAUGCUG |
| 001039 | hsa-miR-492 | hsa-miR-492::mml-miR-492::ppy-miR-492 | MIMAT0002812::MIMAT0006354::MIMAT0015913 | | AGGACCUGCGGGACAAGAUUCUU |
| 001043 | hsa-miR-497 | cfa-miR-497::eca-miR-497::hsa-miR-497-5p::mdo-miR-497::mml-miR-497::ppy-miR-497::sha-miR-497::ssc-miR-497 | MIMAT0002820::MIMAT0006359::MIMAT0006691::MIMAT0012762::MIMAT0013043::MIMAT0013926::MIMAT0015918::MIMAT0022821 | hsa-miR-497(17) | CAGCAGCACACUGUGGUUUGU |
| 001046 | hsa-miR-500 | hsa-miR-500a-3p | MIMAT0002871 | hsa-miR-500(9.2)::hsa-miR-500*(15)::hsa-miR-500a*(17) | AUGCACCUGGGCAAGGAUUCUG |
| 001047 | hsa-miR-501 | hsa-miR-501-5p::mml-miR-501 | MIMAT0002872::MIMAT0006364 | hsa-miR-501(9.2) | AAUCCUUUGUCCCUGGGUGAGA |
| 001048 | hsa-miR-503 | ggo-miR-503::hsa-miR-503-5p::mml-miR-503::ppy-miR-503::ptr-miR-503 | MIMAT0002874::MIMAT0006367::MIMAT0008176::MIMAT0015926::MIMAT0024203 | hsa-miR-503(18) | UAGCAGCGGGAACAGUUCUGCAG |
| 001050 | hsa-miR-506 | hsa-miR-506-3p::mml-miR-506::pbi-miR-506::ppy-miR-506::ptr-miR-506::ssy-miR-506 | MIMAT0002878::MIMAT0005752::MIMAT0005762::MIMAT0005770::MIMAT0005779::MIMAT0015929 | hsa-miR-506(17) | UAAGGCACCCUUCUGAGUAGA |
| 001051 | hsa-miR-507 | hsa-miR-507::mml-miR-507::pbi-miR-507::ppy-miR-507::ptr-miR-507::ssy-miR-507 | MIMAT0002879::MIMAT0005753::MIMAT0005763::MIMAT0005771::MIMAT0005780::MIMAT0015930 | | UUUUGCACCUUUUGGAGUGAA |
| 001052 | hsa-miR-508 | hsa-miR-508-3p | MIMAT0002880 | hsa-miR-508(9.2) | UGAUUGUAGCCUUUUGGAGUAGA |
| 001090 | mmu-miR-93 | cfa-miR-93::cgr-miR-93-5p::eca-miR-93::hsa-miR-93-5p::mmu-miR-93-5p::rno-miR-93-5p | MIMAT0000093::MIMAT0000540::MIMAT0000817::MIMAT0006696::MIMAT0013060::MIMAT0024013 | hsa-miR-93(17)::mmu-miR-93(17)::rno-miR-93(18)::xtr-miR-93a(10) | CAAAGUGCUGUUCGUGCAGGUAG |
| 001094 | RNU44 | RNU44 | NR_002750 | U44 | CCUGGAUGAUGAUAGCAAAUGCUGACUGAACAUGAAGGUCUUAAUUAGCUCUAACUGACU |
| 001097 | hsa-miR-146b | cfa-miR-146b::eca-miR-146b-5p::hsa-miR-146b-5p::mdo-miR-146b::mml-miR-146b-5p::mmu-miR-146b-5p::oan-miR-146b-5p::pma-miR-146-5p::ppy-miR-146b-5p::ptr-miR-146b | MIMAT0002809::MIMAT0003475::MIMAT0006204::MIMAT0006667::MIMAT0007007::MIMAT0008039::MIMAT0012747::MIMAT0012891::MIMAT0015768::MIMAT0019478 | hsa-miR-146b(9.2)::mmu-miR-146b(17)::oan-miR-146b(18)::pma-miR-146(18) | UGAGAACUGAAUUCCAUAGGCU |
| 001101 | hsa-miR-329 | cfa-miR-329b::hsa-miR-329::mml-miR-329::oar-miR-329b-3p::ppy-miR-329::ptr-miR-329 | MIMAT0001629::MIMAT0006269::MIMAT0008101::MIMAT0009899::MIMAT0015832::MIMAT0019264 | | AACACACCUGGUUAACCUCUUU |
| 001102 | hsa-miR-376b | bta-miR-376b::cfa-miR-376b::hsa-miR-376b-3p::mml-miR-376b::ppy-miR-376b::ptr-miR-376b | MIMAT0002172::MIMAT0006304::MIMAT0008127::MIMAT0009887::MIMAT0009945::MIMAT0015865 | hsa-miR-376b(18) | AUCAUAGAGGAAAAUCCAUGUU |
| 001106 | hsa-miR-488 | cfa-miR-488::ggo-miR-488::hsa-miR-488-5p::rno-miR-488-5p | MIMAT0002804::MIMAT0009903::MIMAT0017320::MIMAT0024230 | hsa-miR-488(9.2)::hsa-miR-488*(17)::rno-miR-488*(18) | CCCAGAUAAUGGCACUCUCAA |
| 001109 | hsa-miR-502 | eca-miR-502-5p::hsa-miR-502-5p::mml-miR-502-5p::ppy-miR-502-5p::ptr-miR-502 | MIMAT0002873::MIMAT0006365::MIMAT0008175::MIMAT0013225::MIMAT0015924 | hsa-miR-502(9.2) | AUCCUUGCUAUCUGGGUGCUA |
| 001111 | hsa-miR-511 | hsa-miR-511::ppy-miR-511::ptr-miR-511 | MIMAT0002808::MIMAT0008179::MIMAT0015936 | | GUGUCUUUUGCUCUGCAGUCA |
| 001112 | hsa-miR-515-5p | hsa-miR-515-5p::ppy-miR-515-5p | MIMAT0002826::MIMAT0015943 | | UUCUCCAAAAGAAAGCACUUUCUG |
| 001113 | hsa-miR-517# | hsa-miR-517-5p::ptr-miR-517b | MIMAT0002851::MIMAT0008185 | hsa-miR-517*(17) | CCUCUAGAUGGAAGCACUGUCU |
| 001116 | hsa-miR-520b | hsa-miR-520b::ptr-miR-520b | MIMAT0002843::MIMAT0008198 | mml-miR-520b(15)::mml-miR-520e(15) | AAAGUGCUUCCUUUUAGAGGG |
| 001119 | hsa-miR-520e | hsa-miR-520e::ppy-miR-520e::ptr-miR-520e | MIMAT0002825::MIMAT0008201::MIMAT0015973 | | AAAGUGCUUCCUUUUUGAGGG |
| 001120 | hsa-miR-520f | hsa-miR-520f::ppy-miR-520f::ptr-miR-520f | MIMAT0002830::MIMAT0008202::MIMAT0015974 | mml-miR-520f(15) | AAGUGCUUCCUUUUAGAGGGUU |
| 001121 | hsa-miR-520g | hsa-miR-520g::ppy-miR-520g::ptr-miR-520g::ptr-miR-520h | MIMAT0002858::MIMAT0008203::MIMAT0008204::MIMAT0015975 | | ACAAAGUGCUUCCCUUUAGAGUGU |
| 001122 | hsa-miR-521 | hsa-miR-521::ppy-miR-521::ptr-miR-521 | MIMAT0002854::MIMAT0008205::MIMAT0015977 | | AACGCACUUCCCUUUAGAGUGU |
| 001129 | mmu-miR-137 | aca-miR-137a::bta-miR-137::ccr-miR-137::cgr-miR-137-3p::eca-miR-137::hsa-miR-137::mml-miR-137::mmu-miR-137-3p::oan-miR-137a-3p::ppy-miR-137::ptr-miR-137::rno-miR-137-3p::ssc-miR-137::tgu-miR-137 | MIMAT0000149::MIMAT0000429::MIMAT0000843::MIMAT0006193::MIMAT0006838::MIMAT0008033::MIMAT0009231::MIMAT0012953::MIMAT0014543::MIMAT0015759::MIMAT0021754::MIMAT0023763::MIMAT0025362::MIMAT0026217 | mmu-miR-137(17)::oan-miR-137a(18)::rno-miR-137(18) | UUAUUGCUUAAGAAUACGCGUAG |
| 001138 | mmu-miR-379 | bta-miR-379::cfa-miR-379::cgr-miR-379::eca-miR-379::ggo-miR-379::hsa-miR-379-5p::mml-miR-379::mmu-miR-379-5p::ppy-miR-379::ptr-miR-379::rno-miR-379-5p | MIMAT0000733::MIMAT0000743::MIMAT0003192::MIMAT0006308::MIMAT0006715::MIMAT0008131::MIMAT0009306::MIMAT0013147::MIMAT0015869::MIMAT0023945::MIMAT0024110 | hsa-miR-379(17)::mmu-miR-379(17)::rno-miR-379(18) | UGGUAGACUAUGGAACGUAGG |
| 001141 | mmu-miR-451 | aca-miR-451-5p::cfa-miR-451::dre-miR-451::hsa-miR-451a::mml-miR-451::mmu-miR-451a::ppy-miR-451::ptr-miR-451::rno-miR-451-5p::ssc-miR-451::xtr-miR-451 | MIMAT0001631::MIMAT0001632::MIMAT0001633::MIMAT0001634::MIMAT0003705::MIMAT0006335::MIMAT0008153::MIMAT0009870::MIMAT0015896::MIMAT0018382::MIMAT0021960 | aca-miR-451(18)::hsa-miR-451(17)::mmu-miR-451(18)::rno-miR-451(18) | AAACCGUUACCAUUACUGAGUU |
| 001145 | hsa-miR-512-5p | hsa-miR-512-5p | MIMAT0002822 |  | CACUCAGCCUUGAGGGCACUUUC |
| 001149 | hsa-miR-516-3p | hsa-miR-516a-3p::hsa-miR-516b-3p::ptr-miR-516a | MIMAT0002860::MIMAT0002860::MIMAT0006778::MIMAT0008182 | hsa-miR-516-3p(9.2)::hsa-miR-516b*(17) | UGCUUCCUUUCAGAGGGU |
| 001150 | hsa-miR-516b | hsa-miR-516b-5p::mml-miR-516b::ppy-miR-516b::ptr-miR-516b | MIMAT0002859::MIMAT0008183::MIMAT0012778::MIMAT0015947 | hsa-miR-516-5p(7)::hsa-miR-516b(17)::mml-miR-516(18) | AUCUGGAGGUAAGAAGCACUUU |
| 001152 | hsa-miR-517b | ppy-miR-517b | MIMAT0015949 | hsa-miR-517b(17) | UCGUGCAUCCCUUUAGAGUGUU |
| 001153 | hsa-miR-517c | hsa-miR-517c-3p::mml-miR-517a | MIMAT0002866::MIMAT0006375 | hsa-miR-517c(17)::mml-miR-517(18) | AUCGUGCAUCCUUUUAGAGUGU |
| 001156 | hsa-miR-518b | hsa-miR-518b::ptr-miR-518b | MIMAT0002844::MIMAT0008187 | | CAAAGCGCUCCCCUUUAGAGGU |
| 001158 | hsa-miR-518c# | hsa-miR-518c-5p | MIMAT0002847 | hsa-miR-518c*(17) | UCUCUGGAGGGAAGCACUUUCUG |
| 001159 | hsa-miR-518d | hsa-miR-518d-3p::ptr-miR-518d | MIMAT0002864::MIMAT0008189 | hsa-miR-518d(9.2) | CAAAGCGCUUCCCUUUGGAGC |
| 001163 | hsa-miR-519c | hsa-miR-519c-3p::ptr-miR-519c | MIMAT0002832::MIMAT0008194 | hsa-miR-519c(9.2) | AAAGUGCAUCUUUUUAGAGGAU |
| 001166 | hsa-miR-519e# | hsa-miR-519e-5p | MIMAT0002828 | hsa-miR-519e*(17) | UUCUCCAAAAGGGAGCACUUUC |
| 001167 | hsa-miR-520a | hsa-miR-520a-3p::mml-miR-520a::ppy-miR-520a-3p::ptr-miR-520a | MIMAT0002834::MIMAT0006388::MIMAT0008197::MIMAT0015967 | hsa-miR-520a(9.2) | AAAGUGCUUCCCUUUGGACUGU |
| 001168 | hsa-miR-520a# | hsa-miR-520a-5p::ppy-miR-520a-5p | MIMAT0002833::MIMAT0015966 | hsa-miR-520a*(9.2) | CUCCAGAGGGAAGUACUUUCU |
| 001170 | hsa-miR-520h | hsa-miR-520h::ppy-miR-520h | MIMAT0002867::MIMAT0015976 | | ACAAAGUGCUUCCCUUUAGAGU |
| 001173 | hsa-miR-524 | hsa-miR-524-3p::ppy-miR-524-3p::ptr-miR-524 | MIMAT0002850::MIMAT0008208::MIMAT0015981 | hsa-miR-524(9.2) | GAAGGCGCUUCCCUUUGGAGU |
| 001174 | hsa-miR-525 | hsa-miR-525-5p::mml-miR-525::ppy-miR-525-5p | MIMAT0002838::MIMAT0006402::MIMAT0015982 | hsa-miR-525(9.2) | CUCCAGAGGGAUGCACUUUCU |
| 001178 | mmu-let-7d# | cgr-let-7d-3p::hsa-let-7d-3p::mmu-let-7d-3p::rno-let-7d-3p::ssc-let-7d-3p | MIMAT0000384::MIMAT0000563::MIMAT0004484::MIMAT0023720::MIMAT0025357 | hsa-let-7d*(17)::mmu-let-7d*(17)::rno-let-7d*(18) | CUAUACGACCUGCUGCCUUUCU |
| 001182 | mmu-miR-124a | crm-miR-124::eca-miR-124::hsa-miR-124-3p::mmu-miR-124-3p::ppc-miR-124::ppy-miR-124::rno-miR-124-3p | MIMAT0000134::MIMAT0000422::MIMAT0000828::MIMAT0011541::MIMAT0011645::MIMAT0012906::MIMAT0015747 | hsa-miR-124(17)::mmu-miR-124(17)::mmu-miR-124a(9.2)::rno-miR-124(18) | UAAGGCACGCGGUGAAUGCC |
| 001184 | mmu-miR-129-3p | bta-miR-129-3p::dre-miR-129-3p::eca-miR-129b-3p::hsa-miR-129-2-3p::mmu-miR-129-2-3p::pma-miR-129a-3p::ppy-miR-129-2-3p::rno-miR-129-2-3p::ssc-miR-129a::tgu-miR-129-3p | MIMAT0000544::MIMAT0000601::MIMAT0003158::MIMAT0004605::MIMAT0009222::MIMAT0013046::MIMAT0013959::MIMAT0014499::MIMAT0016972::MIMAT0019451 | dre-miR-129*(18)::hsa-miR-129-3p(17)::mmu-miR-129-3p(15)::pma-miR-129a*(18)::ppy-miR-129-2*(18)::rno-miR-129*(15)::rno-miR-129-2*(18)::ssc-miR-129(16)::tgu-miR-129(18) | AAGCCCUUACCCCAAAAAGCAU |
| 001186 | mmu-miR-134 | cfa-miR-134::cgr-miR-134::eca-miR-134::hsa-miR-134::mml-miR-134::mmu-miR-134-5p::ptr-miR-134::rno-miR-134-5p | MIMAT0000146::MIMAT0000447::MIMAT0000840::MIMAT0006190::MIMAT0008031::MIMAT0009883::MIMAT0013127::MIMAT0023760 | mmu-miR-134(17)::rno-miR-134(18) | UGUGACUGGUUGACCAGAGGGG |
| 001187 | mmu-miR-140 | ccr-miR-140-5p::cgr-miR-140-5p::dre-miR-140-5p::eca-miR-140-5p::fru-miR-140::hsa-miR-140-5p::mdo-miR-140::mml-miR-140-5p::mmu-miR-140-5p::ola-miR-140-5p::pma-miR-140::pol-miR-140-5p::ppy-miR-140-5p::rno-miR-140-5p::tgu-miR-140-5p::tni-miR-140 | MIMAT0000151::MIMAT0000431::MIMAT0000573::MIMAT0001836::MIMAT0003024::MIMAT0003025::MIMAT0006197::MIMAT0012745::MIMAT0012926::MIMAT0014557::MIMAT0015763::MIMAT0019471::MIMAT0022552::MIMAT0023767::MIMAT0025434::MIMAT0026220 | dre-miR-140(18)::mmu-miR-140(17)::rno-miR-140(18)::tgu-miR-140(18) | CAGUGGUUUUACCCUAUGGUAG |
| 001191 | mmu-miR-153 | bta-miR-153::cte-miR-153::dre-miR-153a::eca-miR-153::fru-miR-153a::hsa-miR-153::lgi-miR-153::mdo-miR-153::mmu-miR-153-3p::oan-miR-153-3p::ppy-miR-153::ptr-miR-153::rno-miR-153-3p::tgu-miR-153::tni-miR-153a | MIMAT0000163::MIMAT0000439::MIMAT0000855::MIMAT0001849::MIMAT0002272::MIMAT0003011::MIMAT0003012::MIMAT0007005::MIMAT0008047::MIMAT0009239::MIMAT0009528::MIMAT0009582::MIMAT0012738::MIMAT0012936::MIMAT0014552 | cap-miR-153(14)::mmu-miR-153(17)::oan-miR-153(18)::rno-miR-153(18) | UUGCAUAGUCACAAAAGUGAUC |
| 001193 | mmu-miR-187 | bta-miR-187::cfa-miR-187::eca-miR-187::hsa-miR-187-3p::mml-miR-187::mmu-miR-187-3p::rno-miR-187-3p::ssc-miR-187 | MIMAT0000216::MIMAT0000262::MIMAT0000864::MIMAT0006221::MIMAT0009248::MIMAT0009843::MIMAT0012999::MIMAT0020587 | hsa-miR-187(17)::mmu-miR-187(17)::rno-miR-187(18) | UCGUGUCUUGUGUUGCAGCCGG |
| 001271 | hsa-miR-363 | ccr-miR-363::dre-miR-363::eca-miR-363::hsa-miR-363-3p::mml-miR-363::mmu-miR-363-3p::ppy-miR-363 | MIMAT0000707::MIMAT0000708::MIMAT0001874::MIMAT0006290::MIMAT0013212::MIMAT0015852::MIMAT0026293 | hsa-miR-363(17)::mmu-miR-363(15)::rno-miR-363(15) | AAUUGCACGGUAUCCAUCUGUA |
| 001273 | hsa-miR-362 | bta-miR-362-5p::cfa-miR-362::eca-miR-362-5p::hsa-miR-362-5p::mml-miR-362-5p::ppy-miR-362-5p | MIMAT0000705::MIMAT0006288::MIMAT0009298::MIMAT0009886::MIMAT0013210::MIMAT0015850 | bta-miR-362(13)::hsa-miR-362(9.2) | AAUCCUUGGAACCUAGGUGUGAGU |
| 001274 | hsa-miR-410 | bta-miR-410::cfa-miR-410::cgr-miR-410-3p::eca-miR-410::hsa-miR-410::mml-miR-410::mmu-miR-410-3p::oar-miR-410-3p::ppy-miR-410::ptr-miR-410::rno-miR-410-3p | MIMAT0001091::MIMAT0002171::MIMAT0005311::MIMAT0006316::MIMAT0006728::MIMAT0008137::MIMAT0009311::MIMAT0013153::MIMAT0015877::MIMAT0019334::MIMAT0023953 | mmu-miR-410(17)::rno-miR-410(18) | AAUAUAACACAGAUGGCCUGU |
| 001277 | hsa-miR-485-3p | eca-miR-485-3p::hsa-miR-485-3p::mml-miR-485-3p::oar-miR-485-3p::ppy-miR-485-3p::ptr-miR-485 | MIMAT0002176::MIMAT0006343::MIMAT0008159::MIMAT0013160::MIMAT0015903::MIMAT0019313 | | GUCAUACACGGCUCUCCUCUCU |
| 001278 | hsa-miR-486 | bta-miR-486::cgr-miR-486-5p::eca-miR-486-5p::ggo-miR-486::hsa-miR-486-5p::mml-miR-486-5p::mmu-miR-3107-5p::mmu-miR-486-5p::ppy-miR-486-5p::ptr-miR-486::ssc-miR-486 | MIMAT0002177::MIMAT0003130::MIMAT0006344::MIMAT0008160::MIMAT0009329::MIMAT0013186::MIMAT0013886::MIMAT0014943::MIMAT0015904::MIMAT0023967::MIMAT0024149 | hsa-miR-486(9.2)::mmu-miR-3107(17)::mmu-miR-486(17) | UCCUGUACUGAGCUGCCCCGAG |
| 001279 | hsa-miR-487a | eca-miR-487a::hsa-miR-487a::mml-miR-487a::ppy-miR-487a::ptr-miR-487a | MIMAT0002178::MIMAT0006346::MIMAT0008161::MIMAT0013161::MIMAT0015905 | hsa-miR-487(7.1) | AAUCAUACAGGGACAUCCAGUU |
| 001280 | hsa-miR-455 | cfa-miR-455::cgr-miR-455-5p::hsa-miR-455-5p::mml-miR-455-5p::mmu-miR-455-5p::ppy-miR-455-5p::rno-miR-455-5p::ssc-miR-455-5p | MIMAT0003150::MIMAT0003485::MIMAT0005316::MIMAT0006339::MIMAT0006603::MIMAT0015899::MIMAT0022957::MIMAT0023964 | hsa-miR-455(9.2)::mmu-miR-455(8.1)::mmu-miR-455*(17)::rno-miR-455(18) | UAUGUGCCUUUGGACUACAUCG |
| 001283 | hsa-miR-363# | hsa-miR-363-5p::oan-miR-363-5p::rno-miR-363-5p::xla-miR-363::xtr-miR-363-5p | MIMAT0003209::MIMAT0003385::MIMAT0003701::MIMAT0006859::MIMAT0011147 | hsa-miR-363*(17)::oan-miR-363*(18)::rno-miR-363*(18) | CGGGUGGAUCACGAUGCAAUUU |
| 001284 | hsa-miR-542-3p | cfa-miR-542::eca-miR-542-3p::ggo-miR-542::hsa-miR-542-3p::mml-miR-542-3p::mmu-miR-542-3p::ppy-miR-542-3p::rno-miR-542-3p::ssc-miR-542-3p | MIMAT0003172::MIMAT0003179::MIMAT0003389::MIMAT0006407::MIMAT0006747::MIMAT0013238::MIMAT0013925::MIMAT0015992::MIMAT0024231 | | UGUGACAGAUUGAUAACUGAAA |
| 001285 | hsa-miR-487b | bta-miR-487b::cfa-miR-487b::eca-miR-487b::hsa-miR-487b::mml-miR-487b::mmu-miR-487b-3p::oar-miR-487b-3p::ppy-miR-487b::ptr-miR-487b::rno-miR-487b-3p | MIMAT0003180::MIMAT0003184::MIMAT0003200::MIMAT0003847::MIMAT0006347::MIMAT0006723::MIMAT0008162::MIMAT0013162::MIMAT0015906::MIMAT0019295 | cfa-miR-487(12)::mmu-miR-487b(17)::rno-miR-487b(18) | AAUCGUACAGGGUCAUCCACUU |
| 001286 | hsa-miR-539 | bta-miR-539::cfa-miR-539::hsa-miR-539-5p::mml-miR-539::mmu-miR-539-5p::ppy-miR-539::ptr-miR-539::rno-miR-539-5p | MIMAT0003163::MIMAT0003169::MIMAT0003176::MIMAT0006405::MIMAT0008214::MIMAT0009342::MIMAT0009909::MIMAT0015989 | hsa-miR-539(17)::mmu-miR-539(15)::rno-miR-539(18) | GGAGAAAUUAUCCUUGGUGUGU |
| 001319 | mmu-miR-374-5p | bta-miR-374b::cfa-miR-374b::cgr-miR-374-5p::eca-miR-374b::ggo-miR-374b::hsa-miR-374b-5p::mml-miR-374b::mmu-miR-374b-5p::ptr-miR-374b::rno-miR-374-5p::ssc-miR-374b-5p | MIMAT0003208::MIMAT0003727::MIMAT0004955::MIMAT0006301::MIMAT0006754::MIMAT0008124::MIMAT0009302::MIMAT0013214::MIMAT0013915::MIMAT0023939::MIMAT0024126 | hsa-miR-374b(17)::mmu-miR-374(17)::mmu-miR-374-5p(18)::rno-miR-374(18)::ssc-miR-374b(15) | AUAUAAUACAACCUGCUAAGUG |
| 001338 | rno-miR-7# | hsa-miR-7-1-3p::mmu-miR-7a-1-3p | MIMAT0004553::MIMAT0004670 | hsa-miR-7-1*(17)::mmu-miR-7a*(15)::mmu-miR-7a-1*(17)::rno-miR-7*(9.2) | CAACAAAUCACAGUCUGCCAUA |
| 001352 | mmu-miR-499 | bta-miR-499::cfa-miR-499::eca-miR-499-5p::hsa-miR-499a-5p::mdo-miR-499::mml-miR-499-5p::mmu-miR-499-5p::ppy-miR-499-5p::rno-miR-499-5p::ssc-miR-499-5p | MIMAT0002870::MIMAT0003381::MIMAT0003482::MIMAT0003536::MIMAT0006361::MIMAT0006655::MIMAT0012753::MIMAT0013108::MIMAT0013877::MIMAT0015920 | hsa-miR-499-5p(17)::mmu-miR-499(17)::rno-miR-499(18)::ssc-miR-499(15) | UUAAGACUUGCAGUGAUGUUU |
| 001510 | hsa-miR-656 | bta-miR-656::eca-miR-656::hsa-miR-656::mml-miR-656::ppy-miR-656::ptr-miR-656 | MIMAT0003332::MIMAT0006497::MIMAT0008305::MIMAT0009361::MIMAT0013172::MIMAT0016090 | | AAUAUUAUACAGUCAACCUCU |
| 001511 | hsa-miR-549 | hsa-miR-549a::ppy-miR-549::ptr-miR-549 | MIMAT0003333::MIMAT0008229::MIMAT0015996 | hsa-miR-549(18) | UGACAACUAUGGAUGAGCUCU |
| 001512 | hsa-miR-657 | hsa-miR-657::ptr-miR-657 | MIMAT0003335::MIMAT0008306 | | GGCAGGUUCUCACCCUCUCUAGG |
| 001513 | hsa-miR-658 | hsa-miR-658::ppy-miR-658 | MIMAT0003336::MIMAT0016091 | | GGCGGAGGGAAGUAGGUCCGUUGGU |
| 001514 | hsa-miR-659 | hsa-miR-659-3p | MIMAT0003337 | hsa-miR-659(17) | CUUGGUUCAGGGAGGGUCCCCA |
| 001515 | hsa-miR-660 | cfa-miR-660::eca-miR-660::ggo-miR-660::hsa-miR-660-5p::mml-miR-660::ppy-miR-660::ptr-miR-660 | MIMAT0003338::MIMAT0006499::MIMAT0006760::MIMAT0008308::MIMAT0013241::MIMAT0016092::MIMAT0024164 | hsa-miR-660(17) | UACCCAUUGCAUAUCGGAGUUG |
| 001516 | hsa-miR-425-5p | cfa-miR-425::cgr-miR-425-5p::hsa-miR-425-5p::mml-miR-425::mmu-miR-425-5p::oan-miR-425-5p::ppy-miR-425::ptr-miR-425::rno-miR-425-5p::ssc-miR-425-5p::xtr-miR-425-5p | MIMAT0003393::MIMAT0003640::MIMAT0004750::MIMAT0005314::MIMAT0006324::MIMAT0006639::MIMAT0007154::MIMAT0008144::MIMAT0013917::MIMAT0015885::MIMAT0023958 | hsa-miR-425(17)::mmu-miR-425(17)::oan-miR-425(18)::rno-miR-425(18) | AAUGACACGAUCACUCCCGUUGA |
| 001518 | hsa-miR-532 | bta-miR-532::cfa-miR-532::cgr-miR-532-5p::eca-miR-532-5p::hsa-miR-532-5p::mml-miR-532-5p::mmu-miR-532-5p::ppy-miR-532-5p::ssc-miR-532-5p | MIMAT0002888::MIMAT0002889::MIMAT0003848::MIMAT0006403::MIMAT0006758::MIMAT0013235::MIMAT0013940::MIMAT0015987::MIMAT0023981 | hsa-miR-532(9.2)::mmu-miR-532(9.2) | CAUGCCUUGAGUGUAGGACCGU |
| 001519 | hsa-miR-551a | cfa-miR-551a::eca-miR-551a::ggo-miR-551a::hsa-miR-551a::mml-miR-551a::ppy-miR-551a::ptr-miR-551a | MIMAT0003214::MIMAT0006419::MIMAT0008231::MIMAT0009912::MIMAT0012921::MIMAT0015997::MIMAT0024186 | | GCGACCCACUCUUGGUUUCCA |
| 001520 | hsa-miR-552 | hsa-miR-552::ptr-miR-552 | MIMAT0003215::MIMAT0008233 | | AACAGGUGACUGGUUAGACAA |
| 001521 | hsa-miR-553 | hsa-miR-553::ptr-miR-553 | MIMAT0003216::MIMAT0008234 | | AAAACGGUGAGAUUUUGUUUU |
| 001522 | hsa-miR-554 | hsa-miR-554::mml-miR-554::ptr-miR-554 | MIMAT0003217::MIMAT0006423::MIMAT0008235 | | GCUAGUCCUGACUCAGCCAGU |
| 001523 | hsa-miR-555 | hsa-miR-555::ptr-miR-555 | MIMAT0003219::MIMAT0008236 | | AGGGUAAGCUGAACCUCUGAU |
| 001525 | hsa-miR-557 | hsa-miR-557::ptr-miR-557 | MIMAT0003221::MIMAT0008238 | | GUUUGCACGGGUGGGCCUUGUCU |
| 001526 | hsa-miR-558 | hsa-miR-558::mml-miR-558::ppy-miR-558::ptr-miR-558 | MIMAT0003222::MIMAT0006427::MIMAT0008239::MIMAT0016005 | | UGAGCUGCUGUACCAAAAU |
| 001527 | hsa-miR-559 | hsa-miR-559::ptr-miR-559 | MIMAT0003223::MIMAT0008240 | | UAAAGUAAAUAUGCACCAAAA |
| 001528 | hsa-miR-561 | hsa-miR-561-3p | MIMAT0003225 | hsa-miR-561(17) | CAAAGUUUAAGAUCCUUGAAGU |
| 001529 | hsa-miR-562 | hsa-miR-562::mml-miR-562::ppy-miR-562::ptr-miR-562 | MIMAT0003226::MIMAT0006428::MIMAT0008242::MIMAT0016007 | | AAAGUAGCUGUACCAUUUGC |
| 001530 | hsa-miR-563 | hsa-miR-563 | MIMAT0003227 |  | AGGUUGACAUACGUUUCCC |
| 001531 | hsa-miR-564 | hsa-miR-564::ptr-miR-564 | MIMAT0003228::MIMAT0008243 | | AGGCACGGUGUCAGCAGGC |
| 001533 | hsa-miR-566 | hsa-miR-566::ppy-miR-566::ptr-miR-566 | MIMAT0003230::MIMAT0008244::MIMAT0016010 | | GGGCGCCUGUGAUCCCAAC |
| 001534 | hsa-miR-567 | hsa-miR-567::ptr-miR-567 | MIMAT0003231::MIMAT0008245 | | AGUAUGUUCUUCCAGGACAGAAC |
| 001535 | hsa-miR-551b | aca-miR-551::cfa-miR-551b::eca-miR-551b::gga-miR-551-3p::ggo-miR-551b::hsa-miR-551b-3p::mdo-miR-551b::mml-miR-551b::mmu-miR-551b-3p::oan-miR-551-3p::ppy-miR-551b::ptr-miR-551b::tgu-miR-551-3p | MIMAT0003233::MIMAT0003890::MIMAT0006420::MIMAT0007130::MIMAT0007290::MIMAT0008232::MIMAT0009913::MIMAT0012764::MIMAT0013094::MIMAT0014541::MIMAT0015998::MIMAT0021982::MIMAT0024188 | gga-miR-551(18)::hsa-miR-551b(17)::mmu-miR-551b(17)::oan-miR-551(18)::tgu-miR-551(18) | GCGACCCAUACUUGGUUUCAG |
| 001536 | hsa-miR-569 | hsa-miR-569::mml-miR-569::ppy-miR-569::ptr-miR-569 | MIMAT0003234::MIMAT0006432::MIMAT0008247::MIMAT0016013 | | AGUUAAUGAAUCCUGGAAAGU |
| 001538 | hsa-miR-548a | hsa-miR-548a-3p::ptr-miR-548a | MIMAT0003251::MIMAT0008218 | hsa-miR-548a(9.2) | CAAAACUGGCAAUUACUUUUGC |
| 001539 | hsa-miR-586 | hsa-miR-586::ppy-miR-586 | MIMAT0003252::MIMAT0016028 | | UAUGCAUUGUAUUUUUAGGUCC |
| 001540 | hsa-miR-587 | hsa-miR-587::ppy-miR-587::ptr-miR-587 | MIMAT0003253::MIMAT0008259::MIMAT0016029 | | UUUCCAUAGGUGAUGAGUCAC |
| 001541 | hsa-miR-548b | hsa-miR-548b-3p::ptr-miR-548b | MIMAT0003254::MIMAT0008219 | hsa-miR-548b(9.2) | CAAGAACCUCAGUUGCUUUUGU |
| 001542 | hsa-miR-588 | hsa-miR-588::ppy-miR-588::ptr-miR-588 | MIMAT0003255::MIMAT0008260::MIMAT0016030 | | UUGGCCACAAUGGGUUAGAAC |
| 001543 | hsa-miR-589 | hsa-miR-589-3p | MIMAT0003256 | hsa-miR-589(9.2)::hsa-miR-589*(17) | UCAGAACAAAUGCCGGUUCCCAGA |
| 001544 | hsa-miR-550 | hsa-miR-550a-3p | MIMAT0003257 | hsa-miR-550(9.2)::hsa-miR-550*(15)::hsa-miR-550a*(17) | UGUCUUACUCCCUCAGGCACAU |
| 001545 | hsa-miR-591 | hsa-miR-591::ppy-miR-591::ptr-miR-591 | MIMAT0003259::MIMAT0008262::MIMAT0016034 | | AGACCAUGGGUUCUCAUUGU |
| 001546 | hsa-miR-592 | eca-miR-592::hsa-miR-592::mml-miR-592::ppy-miR-592::ptr-miR-592 | MIMAT0003260::MIMAT0006452::MIMAT0008263::MIMAT0012946::MIMAT0016035 | | UUGUGUCAAUAUGCGAUGAUGU |
| 001547 | hsa-miR-593 | hsa-miR-593-5p | MIMAT0003261 | hsa-miR-593(9.2)::hsa-miR-593*(17) | AGGCACCAGCCAGGCAUUGCUCAGC |
| 001550 | hsa-miR-596 | hsa-miR-596 | MIMAT0003264 |  | AAGCCUGCCCGGCUCCUCGGG |
| 001551 | hsa-miR-597 | hsa-miR-597::ppy-miR-597::ptr-miR-597 | MIMAT0003265::MIMAT0008266::MIMAT0016039 | | UGUGUCACUCGAUGACCACUGU |
| 001553 | hsa-miR-622 | hsa-miR-622::mml-miR-622::ptr-miR-622 | MIMAT0003291::MIMAT0008283::MIMAT0012782 | | ACAGUCUGCUGAGGUUGGAGC |
| 001554 | hsa-miR-599 | bta-miR-599::cfa-miR-599::hsa-miR-599::mdo-miR-599::mml-miR-599::ppy-miR-599::ptr-miR-599 | MIMAT0003267::MIMAT0006456::MIMAT0008268::MIMAT0009354::MIMAT0009919::MIMAT0012765::MIMAT0016041 | | GUUGUGUCAGUUUAUCAAAC |
| 001555 | hsa-miR-623 | hsa-miR-623 | MIMAT0003292 |  | AUCCCUUGCAGGGGCUGUUGGGU |
| 001556 | hsa-miR-600 | hsa-miR-600::ptr-miR-600 | MIMAT0003268::MIMAT0008269 | | ACUUACAGACAAGAGCCUUGCUC |
| 001557 | hsa-miR-624 | ggo-miR-624::hsa-miR-624-5p | MIMAT0003293::MIMAT0024302 | hsa-miR-624(9.2)::hsa-miR-624*(17) | UAGUACCAGUACCUUGUGUUCA |
| 001558 | hsa-miR-601 | hsa-miR-601::mml-miR-601::ppy-miR-601::ptr-miR-601 | MIMAT0003269::MIMAT0006458::MIMAT0008270::MIMAT0016043 | | UGGUCUAGGAUUGUUGGAGGAG |
| 001559 | hsa-miR-626 | hsa-miR-626::ptr-miR-626 | MIMAT0003295::MIMAT0008285 | | AGCUGUCUGAAAAUGUCUU |
| 001560 | hsa-miR-627 | hsa-miR-627::ptr-miR-627 | MIMAT0003296::MIMAT0008286 | | GUGAGUCUCUAAGAAAAGAGGA |
| 001562 | hsa-miR-629 | hsa-miR-629-3p | MIMAT0003298 | hsa-miR-629(9.2)::hsa-miR-629*(17) | GUUCUCCCAACGUAAGCCCAGC |
| 001563 | hsa-miR-630 | hsa-miR-630::ppy-miR-630::ptr-miR-630 | MIMAT0003299::MIMAT0008288::MIMAT0016069 | | AGUAUUCUGUACCAGGGAAGGU |
| 001564 | hsa-miR-631 | hsa-miR-631::mml-miR-631::ppy-miR-631 | MIMAT0003300::MIMAT0006476::MIMAT0016070 | | AGACCUGGCCCAGACCUCAGC |
| 001566 | hsa-miR-603 | hsa-miR-603::ppy-miR-603 | MIMAT0003271::MIMAT0016045 | | CACACACUGCAAUUACUUUUGC |
| 001567 | hsa-miR-604 | hsa-miR-604 | MIMAT0003272 |  | AGGCUGCGGAAUUCAGGAC |
| 001568 | hsa-miR-605 | hsa-miR-605::ppy-miR-605::ptr-miR-605 | MIMAT0003273::MIMAT0008271::MIMAT0016047 | | UAAAUCCCAUGGUGCCUUCUCCU |
| 001569 | hsa-miR-606 | hsa-miR-606 | MIMAT0003274 |  | AAACUACUGAAAAUCAAAGAU |
| 001570 | hsa-miR-607 | hsa-miR-607 | MIMAT0003275 |  | GUUCAAAUCCAGAUCUAUAAC |
| 001571 | hsa-miR-608 | hsa-miR-608 | MIMAT0003276 |  | AGGGGUGGUGUUGGGACAGCUCCGU |
| 001573 | hsa-miR-609 | hsa-miR-609::ptr-miR-609 | MIMAT0003277::MIMAT0008272 | | AGGGUGUUUCUCUCAUCUCU |
| 001574 | hsa-miR-633 | hsa-miR-633::mml-miR-633 | MIMAT0003303::MIMAT0006478 | | CUAAUAGUAUCUACCACAAUAAA |
| 001576 | hsa-miR-634 | hsa-miR-634::ptr-miR-634 | MIMAT0003304::MIMAT0008291 | | AACCAGCACCCCAACUUUGGAC |
| 001578 | hsa-miR-635 | hsa-miR-635::ptr-miR-635 | MIMAT0003305::MIMAT0008292 | | ACUUGGGCACUGAAACAAUGUCC |
| 001581 | hsa-miR-637 | hsa-miR-637::ptr-miR-637 | MIMAT0003307::MIMAT0008293 | | ACUGGGGGCUUUCGGGCUCUGCGU |
| 001582 | hsa-miR-638 | hsa-miR-638 | MIMAT0003308 |  | AGGGAUCGCGGGCGGGUGGCGGCCU |
| 001583 | hsa-miR-639 | hsa-miR-639::ppy-miR-639 | MIMAT0003309::MIMAT0016077 | | AUCGCUGCGGUUGCGAGCGCUGU |
| 001584 | hsa-miR-640 | hsa-miR-640::mml-miR-640::ppy-miR-640::ptr-miR-640 | MIMAT0003310::MIMAT0006482::MIMAT0008294::MIMAT0016078 | | AUGAUCCAGGAACCUGCCUCU |
| 001585 | hsa-miR-641 | hsa-miR-641 | MIMAT0003311 |  | AAAGACAUAGGAUAGAGUCACCUC |
| 001586 | hsa-miR-613 | hsa-miR-613::ptr-miR-613 | MIMAT0003281::MIMAT0008275 | | AGGAAUGUUCCUUCUUUGCC |
| 001587 | hsa-miR-614 | hsa-miR-614::ppy-miR-614::ptr-miR-614 | MIMAT0003282::MIMAT0008276::MIMAT0016055 | | GAACGCCUGUUCUUGCCAGGUGG |
| 001589 | hsa-miR-616 | hsa-miR-616-5p | MIMAT0003284 | hsa-miR-616(9.2)::hsa-miR-616*(17) | ACUCAAAACCCUUCAGUGACUU |
| 001590 | hsa-miR-548c | hsa-miR-548c-3p::ptr-miR-548c | MIMAT0003285::MIMAT0008220 | hsa-miR-548c(9.2) | CAAAAAUCUCAAUUACUUUUGC |
| 001591 | hsa-miR-617 | hsa-miR-617::ptr-miR-617 | MIMAT0003286::MIMAT0008279 | | AGACUUCCCAUUUGAAGGUGGC |
| 001592 | hsa-miR-642 | hsa-miR-642a-5p::mml-miR-642::ptr-miR-642 | MIMAT0003312::MIMAT0006483::MIMAT0008296 | hsa-miR-642(15)::hsa-miR-642a(17) | GUCCCUCUCCAAAUGUGUCUUG |
| 001593 | hsa-miR-618 | hsa-miR-618::mml-miR-618::ppy-miR-618::ptr-miR-618 | MIMAT0003287::MIMAT0006468::MIMAT0008280::MIMAT0016060 | | AAACUCUACUUGUCCUUCUGAGU |
| 001594 | hsa-miR-643 | hsa-miR-643::ptr-miR-643 | MIMAT0003313::MIMAT0008297 | | ACUUGUAUGCUAGCUCAGGUAG |
| 001596 | hsa-miR-644 | hsa-miR-644a | MIMAT0003314 | hsa-miR-644(17) | AGUGUGGCUUUCUUAGAGC |
| 001597 | hsa-miR-645 | hsa-miR-645::ppy-miR-645::ptr-miR-645 | MIMAT0003315::MIMAT0008298::MIMAT0016079 | | UCUAGGCUGGUACUGCUGA |
| 001598 | hsa-miR-621 | hsa-miR-621::ptr-miR-621 | MIMAT0003290::MIMAT0008282 | | GGCUAGCAACAGCGCUUACCU |
| 001599 | hsa-miR-646 | hsa-miR-646::ptr-miR-646 | MIMAT0003316::MIMAT0008299 | | AAGCAGCUGCCUCUGAGGC |
| 001600 | hsa-miR-647 | hsa-miR-647 | MIMAT0003317 |  | GUGGCUGCACUCACUUCCUUC |
| 001601 | hsa-miR-648 | hsa-miR-648::ppy-miR-648 | MIMAT0003318::MIMAT0016082 | | AAGUGUGCAGGGCACUGGU |
| 001602 | hsa-miR-649 | hsa-miR-649::mml-miR-649::ptr-miR-649 | MIMAT0003319::MIMAT0006487::MIMAT0008300 | | AAACCUGUGUUGUUCAAGAGUC |
| 001603 | hsa-miR-650 | hsa-miR-650::mml-miR-650a::ptr-miR-650 | MIMAT0003320::MIMAT0006488::MIMAT0008301 | | AGGAGGCAGCGCUCUCAGGAC |
| 001604 | hsa-miR-651 | ggo-miR-651::hsa-miR-651 | MIMAT0003321::MIMAT0024274 | | UUUAGGAUAAGCUUGACUUUUG |
| 001605 | hsa-miR-548d | hsa-miR-548d-3p | MIMAT0003323 | hsa-miR-548d(9.2) | CAAAAACCACAGUUUCUUUUGC |
| 001606 | hsa-miR-661 | hsa-miR-661 | MIMAT0003324 |  | UGCCUGGGUCUCUGGCCUGCGCGU |
| 001607 | hsa-miR-662 | hsa-miR-662::mml-miR-662 | MIMAT0003325::MIMAT0006501 | | UCCCACGUUGUGGCCCAGCAG |
| 001608 | hsa-miR-449b | bta-miR-449b::hsa-miR-449b-5p::mml-miR-449b::ppy-miR-449b::ptr-miR-449b | MIMAT0003327::MIMAT0006331::MIMAT0008150::MIMAT0009321::MIMAT0015892 | hsa-miR-449b(17) | AGGCAGUGUAUUGUUAGCUGGC |
| 001610 | hsa-miR-411 | eca-miR-411::hsa-miR-411-5p::mml-miR-411::mmu-miR-411-5p::ppy-miR-411::ptr-miR-411::rno-miR-411-5p | MIMAT0003329::MIMAT0004747::MIMAT0005312::MIMAT0006317::MIMAT0008138::MIMAT0013154::MIMAT0015878 | hsa-miR-411(17)::mmu-miR-411(17)::rno-miR-411(18) | UAGUAGACCGUAUAGCGUACG |
| 001611 | hsa-miR-654 | ggo-miR-654::hsa-miR-654-5p::mml-miR-654-5p::ppy-miR-654-5p | MIMAT0003330::MIMAT0006495::MIMAT0016087::MIMAT0024238 | hsa-miR-654(9.2) | UGGUGGGCCGCAGAACAUGUGC |
| 001612 | hsa-miR-655 | eca-miR-655::hsa-miR-655::ppy-miR-655 | MIMAT0003331::MIMAT0013171::MIMAT0016089 | | AUAAUACAUGGUUAACCUCUUU |
| 001613 | hsa-miR-571 | hsa-miR-571::ppy-miR-571 | MIMAT0003236::MIMAT0016014 | | UGAGUUGGCCAUCUGAGUGAG |
| 001614 | hsa-miR-572 | hsa-miR-572::mml-miR-572::ptr-miR-572 | MIMAT0003237::MIMAT0006434::MIMAT0008248 | | GUCCGCUCGGCGGUGGCCCA |
| 001615 | hsa-miR-573 | hsa-miR-573 | MIMAT0003238 |  | CUGAAGUGAUGUGUAACUGAUCAG |
| 001617 | hsa-miR-575 | hsa-miR-575::ptr-miR-575 | MIMAT0003240::MIMAT0008249 | | GAGCCAGUUGGACAGGAGC |
| 001619 | hsa-miR-578 | hsa-miR-578::ppy-miR-578 | MIMAT0003243::MIMAT0016020 | | CUUCUUGUGCUCUAGGAUUGU |
| 001621 | hsa-miR-580 | hsa-miR-580::ptr-miR-580 | MIMAT0003245::MIMAT0008253 | | UUGAGAAUGAUGAAUCAUUAGG |
| 001622 | hsa-miR-581 | hsa-miR-581::ppy-miR-581::ptr-miR-581 | MIMAT0003246::MIMAT0008254::MIMAT0016023 | | UCUUGUGUUCUCUAGAUCAGU |
| 001623 | hsa-miR-583 | hsa-miR-583::ptr-miR-583 | MIMAT0003248::MIMAT0008256 | | CAAAGAGGAAGGUCCCAUUAC |
| 001624 | hsa-miR-584 | hsa-miR-584-5p::mml-miR-584::ppy-miR-584::ptr-miR-584 | MIMAT0003249::MIMAT0006446::MIMAT0008257::MIMAT0016027 | hsa-miR-584(17) | UUAUGGUUUGCCUGGGACUGAG |
| 001625 | hsa-miR-585 | hsa-miR-585 | MIMAT0003250 |  | UGGGCGUAUCUGUAUGCUA |
| 001630 | mmu-miR-491 | bta-miR-491::eca-miR-491-5p::hsa-miR-491-5p::mml-miR-491-5p::mmu-miR-491-5p::ppy-miR-491-5p::ssc-miR-491 | MIMAT0002807::MIMAT0003486::MIMAT0006352::MIMAT0009332::MIMAT0013116::MIMAT0015911::MIMAT0020592 | mmu-miR-491(17) | AGUGGGGAACCCUUCCAUGAGG |
| 001663 | mmu-miR-495 | bta-miR-495::cfa-miR-495::eca-miR-495::hsa-miR-495-3p::mml-miR-495::mmu-miR-495-3p::oar-miR-495-3p::ppy-miR-495::ptr-miR-495::rno-miR-495 | MIMAT0002817::MIMAT0003456::MIMAT0005320::MIMAT0006357::MIMAT0006721::MIMAT0008169::MIMAT0009335::MIMAT0013165::MIMAT0015916::MIMAT0019274 | hsa-miR-495(18)::mmu-miR-495(17) | AAACAAACAUGGUGCACUUCUU |
| 001818 | rno-miR-29c# | hsa-miR-29c-5p::mmu-miR-29c-5p::rno-miR-29c-5p | MIMAT0003154::MIMAT0004632::MIMAT0004673 | hsa-miR-29c*(17)::mmu-miR-29c*(17)::rno-miR-29c*(18) | UGACCGAUUUCUCCUGGUGUUC |
| 001821 | hsa-miR-484 | bta-miR-484::cgr-miR-484::hsa-miR-484::mml-miR-484::mmu-miR-484::ppy-miR-484::ptr-miR-484::rno-miR-484 | MIMAT0002174::MIMAT0003127::MIMAT0003535::MIMAT0005319::MIMAT0006341::MIMAT0008158::MIMAT0015901::MIMAT0023966 | | UCAGGCUCAGUCCCCUCCCGAU |
| 001823 | hsa-miR-512-3p | hsa-miR-512-3p::ppy-miR-512-3p::ptr-miR-512 | MIMAT0002823::MIMAT0008180::MIMAT0015937 | | AAGUGCUGUCAUAGCUGAGGUC |
| 001953 | mmu-miR-496 | bta-miR-496::cfa-miR-496::eca-miR-496::hsa-miR-496::mml-miR-496::mmu-miR-496a-3p::ppy-miR-496::ptr-miR-496 | MIMAT0002818::MIMAT0003738::MIMAT0006358::MIMAT0008170::MIMAT0009336::MIMAT0009906::MIMAT0013166::MIMAT0015917 | mmu-miR-496(17)::mmu-miR-496-3p(18)::rno-miR-496(15) | UGAGUAUUACAUGGCCAAUCUC |
| 001960 | mmu-miR-615 | cgr-miR-615-3p::hsa-miR-615-3p::mml-miR-615-3p::mmu-miR-615-3p::ptr-miR-615 | MIMAT0003283::MIMAT0003783::MIMAT0006466::MIMAT0008277::MIMAT0023988 | mmu-miR-615(9.2) | UCCGAGCCUGGGUCUCCCUCUU |
| 001973 | U6 snRNA | U6 snRNA | NR_004394 |  | GUGCUCGCUUCGGCAGCACAUAUACUAAAAUUGGAACGAUACAGAGAAGAUUAGCAUGGCCCCUGCGCAAGGAUGACACGCAAAUUCGUGAAGCGUUCCAUAUUUU |
| 001979 | hsa-miR-431 | hsa-miR-431-5p::mml-miR-431::mmu-miR-431-5p::ppy-miR-431::ptr-miR-431::rno-miR-431 | MIMAT0001418::MIMAT0001625::MIMAT0001626::MIMAT0006326::MIMAT0008145::MIMAT0015887 | hsa-miR-431(17)::mmu-miR-431(17) | UGUCUUGCAGGCCGUCAUGCA |
| 001982 | hsa-miR-524-5p | hsa-miR-524-5p::ppy-miR-524-5p | MIMAT0002849::MIMAT0015980 | hsa-miR-524*(9.2) | CUACAAAGGGAAGCACUUUCUC |
| 001983 | hsa-miR-582-5p | bta-miR-582::eca-miR-582-5p::hsa-miR-582-5p::mml-miR-582-5p::ppy-miR-582-5p | MIMAT0003247::MIMAT0006443::MIMAT0009351::MIMAT0013103::MIMAT0016024 | hsa-miR-582(9.2) | UUACAGUUGUUCAACCAGUUACU |
| 001984 | hsa-miR-590-5p | ggo-miR-590::hsa-miR-590-5p::mml-miR-590-5p::mmu-miR-590-5p::ppy-miR-590-5p | MIMAT0003258::MIMAT0004895::MIMAT0006450::MIMAT0016032::MIMAT0024206 | hsa-miR-590(9.2) | GAGCUUAUUCAUAAAAGUGCAG |
| 001986 | hsa-miR-766 | ggo-miR-766::hsa-miR-766-3p::ppy-miR-766::ptr-miR-766 | MIMAT0003888::MIMAT0008321::MIMAT0016106::MIMAT0024224 | hsa-miR-766(17) | ACUCCAGCCCCACAGCCUCAGC |
| 001987 | hsa-miR-595 | hsa-miR-595::ptr-miR-595 | MIMAT0003263::MIMAT0008265 | | GAAGUGUGCCGUGGUGUGUCU |
| 001988 | hsa-miR-598 | eca-miR-598::ggo-miR-598::hsa-miR-598::mml-miR-598::ppy-miR-598::ptr-miR-598 | MIMAT0003266::MIMAT0006455::MIMAT0008267::MIMAT0012922::MIMAT0016040::MIMAT0024089 | | UACGUCAUCGUUGUCAUCGUCA |
| 001990 | hsa-miR-758 | bta-miR-758::cfa-miR-758::eca-miR-758::hsa-miR-758-3p::ppy-miR-758::ptr-miR-758::rno-miR-758-3p | MIMAT0003879::MIMAT0005335::MIMAT0008318::MIMAT0009370::MIMAT0009925::MIMAT0013173::MIMAT0016103 | hsa-miR-758(18)::rno-miR-758(18) | UUUGUGACCUGGUCCACUAACC |
| 001992 | hsa-miR-668 | hsa-miR-668::mml-miR-668::ppy-miR-668::ptr-miR-668 | MIMAT0003881::MIMAT0006504::MIMAT0008313::MIMAT0016095 | | UGUCACUCGGCUCGGCCCACUAC |
| 001993 | hsa-miR-767-5p | bta-miR-767::eca-miR-767-5p::hsa-miR-767-5p::mml-miR-767-5p::ppy-miR-767-5p | MIMAT0003882::MIMAT0006510::MIMAT0009375::MIMAT0013245::MIMAT0016107 | | UGCACCAUGGUUGUCUGAGCAUG |
| 001995 | hsa-miR-767-3p | hsa-miR-767-3p::mml-miR-767-3p::ppy-miR-767-3p | MIMAT0003883::MIMAT0006511::MIMAT0016108 | | UCUGCUCAUACCCCAUGGUUUCU |
| 001996 | hsa-miR-454# | hsa-miR-454-5p | MIMAT0003884 | hsa-miR-454*(17) | ACCCUAUCAAUAUUGUCUCUGC |
| 001998 | hsa-miR-769-5p | hsa-miR-769-5p | MIMAT0003886 |  | UGAGACCUCUGGGUUCUGAGCU |
| 002002 | hsa-miR-770-5p | hsa-miR-770-5p::mml-miR-770-5p::ppy-miR-770-5p::ptr-miR-770 | MIMAT0003948::MIMAT0006514::MIMAT0008322::MIMAT0016109 | | UCCAGUACCACGUGUCAGGGCCA |
| 002003 | hsa-miR-769-3p | hsa-miR-769-3p | MIMAT0003887 |  | CUGGGAUCUCCGGGGUCUUGGUU |
| 002004 | hsa-miR-802 | cfa-miR-802::eca-miR-802::hsa-miR-802::mml-miR-802::ppy-miR-802::ptr-miR-802 | MIMAT0004185::MIMAT0006515::MIMAT0008323::MIMAT0009928::MIMAT0013183::MIMAT0016110 | | CAGUAACAAAGAUUCAUCCUUGU |
| 002005 | hsa-miR-675 | ggo-miR-675b::hsa-miR-675-5p::mml-miR-675::ppy-miR-675a | MIMAT0004284::MIMAT0006507::MIMAT0016098::MIMAT0024305 | hsa-miR-675(17) | UGGUGCGGAGAGGGCCCACAGUG |
| 002021 | hsa-miR-674 | mmu-miR-674-5p::rno-miR-674-5p | MIMAT0003740::MIMAT0005329 | hsa-miR-674(10)::mmu-miR-674(17) | GCACUGAGAUGGGAGUGGUGUA |
| 002083 | hsa-miR-502-3p | bta-miR-502a::cfa-miR-502::eca-miR-502-3p::ggo-miR-502a::hsa-miR-502-3p::mml-miR-502-3p::ppy-miR-501-3p::ppy-miR-502-3p | MIMAT0004775::MIMAT0006366::MIMAT0006761::MIMAT0009338::MIMAT0013226::MIMAT0015923::MIMAT0015925::MIMAT0024187 | | AAUGCACCUGGGCAAGGAUUCA |
| 002084 | hsa-miR-504 | cfa-miR-504::eca-miR-504::hsa-miR-504::mml-miR-504::mmu-miR-504-5p::ppy-miR-504::ptr-miR-504 | MIMAT0002875::MIMAT0004889::MIMAT0006368::MIMAT0008177::MIMAT0009907::MIMAT0013228::MIMAT0015927 | mmu-miR-504(17) | AGACCCUGGUCUGCACUCUAUC |
| 002085 | hsa-miR-33b | bta-miR-33b::cfa-miR-33b::eca-miR-33b::hsa-miR-33b-5p::mml-miR-33b::ptr-miR-33b | MIMAT0003301::MIMAT0006173::MIMAT0008108::MIMAT0009295::MIMAT0009862::MIMAT0013037 | hsa-miR-33b(17) | GUGCAUUGCUGUUGCAUUGC |
| 002087 | hsa-miR-505# | cfa-miR-505::hsa-miR-505-5p | MIMAT0004776::MIMAT0009908 | hsa-miR-505*(17) | GGGAGCCAGGAAGUAUUGAUGU |
| 002088 | hsa-miR-636 | hsa-miR-636 | MIMAT0003306 |  | UGUGCUUGCUCGUCCCGCCCGCA |
| 002089 | hsa-miR-505 | bta-miR-505::eca-miR-505::hsa-miR-505-3p::mml-miR-505::ppy-miR-505::ptr-miR-505 | MIMAT0002876::MIMAT0006369::MIMAT0008178::MIMAT0009341::MIMAT0013229::MIMAT0015928 | hsa-miR-505(17) | CGUCAACACUUGCUGGUUUCCU |
| 002090 | hsa-miR-513-5p | hsa-miR-513a-5p::ppy-miR-513a-5p | MIMAT0002877::MIMAT0015938 | hsa-miR-513-5p(10) | UUCACAGGGAGGUGUCAU |
| 002092 | hsa-miR-508-5p | hsa-miR-508-5p | MIMAT0004778 |  | UACUCCAGAGGGCGUCACUCAUG |
| 002093 | hsa-miR-486-3p | eca-miR-486-3p::hsa-miR-486-3p::mml-miR-486-3p::mmu-miR-486-3p | MIMAT0004762::MIMAT0006345::MIMAT0013187::MIMAT0017206 | mmu-miR-486*(17) | CGGGGCAGCUCAGUACAGGAU |
| 002094 | hsa-miR-218-1# | hsa-miR-218-1-3p | MIMAT0004565 | hsa-miR-218-1*(17) | AUGGUUCCGUCAAGCACCAUGG |
| 002095 | hsa-miR-219-1-3p | hsa-miR-219-1-3p::ptr-miR-219-1-3p::ssc-miR-219 | MIMAT0004567::MIMAT0008074::MIMAT0020590 | | AGAGUUGAGUCUGGACGUCCCG |
| 002096 | hsa-miR-221# | hsa-miR-221-5p::pol-miR-221-5p | MIMAT0004568::MIMAT0025448 | hsa-miR-221*(17) | ACCUGGCAUACAAUGUAGAUUU |
| 002097 | hsa-miR-222# | hsa-miR-222-5p | MIMAT0004569 | hsa-miR-222*(17) | CUCAGUAGCCAGUGUAGAUCCU |
| 002098 | hsa-miR-223# | hsa-miR-223-5p | MIMAT0004570 | hsa-miR-223*(17) | CGUGUAUUUGACAAGCUGAGUU |
| 002099 | hsa-miR-224 | eca-miR-224::hsa-miR-224-5p | MIMAT0000281::MIMAT0013206 | hsa-miR-224(17) | CAAGUCACUAGUGGUUCCGUU |
| 002100 | hsa-miR-136# | hsa-miR-136-3p::rno-miR-136-3p | MIMAT0004606::MIMAT0004733 | hsa-miR-136*(17)::rno-miR-136*(18) | CAUCAUCGUCUCAAAUGAGUCU |
| 002101 | hsa-miR-296-3p | hsa-miR-296-3p::mml-miR-296-3p::mmu-miR-296-3p::ppy-miR-296-3p::ptr-miR-296::rno-miR-296-3p | MIMAT0004576::MIMAT0004679::MIMAT0004742::MIMAT0006252::MIMAT0008079::MIMAT0015810 | rno-miR-296(18) | GAGGGUUGGGUGGAGGCUCUCC |
| 002102 | hsa-miR-34b | hsa-miR-34b-3p::mml-miR-34b::ppy-miR-34b::ptr-miR-34b | MIMAT0004676::MIMAT0006174::MIMAT0008113::MIMAT0015741 | hsa-miR-34b(17) | CAAUCACUAACUCCACUGCCAU |
| 002104 | hsa-miR-185# | hsa-miR-185-3p | MIMAT0004611 | hsa-miR-185*(17) | AGGGGCUGGCUUUCCUCUGGUC |
| 002105 | hsa-miR-186# | hsa-miR-186-3p | MIMAT0004612 | hsa-miR-186*(17) | GCCCAAAGGUGAAUUUUUUGGG |
| 002106 | hsa-miR-188-3p | eca-miR-188-3p::hsa-miR-188-3p::mmu-miR-188-3p | MIMAT0004541::MIMAT0004613::MIMAT0013199 | | CUCCCACAUGCAGGGUUUGCA |
| 002107 | hsa-miR-195# | hsa-miR-195-3p::mmu-miR-195a-3p | MIMAT0004615::MIMAT0017000 | hsa-miR-195*(17)::mmu-miR-195*(17)::mmu-miR-195-3p(18) | CCAAUAUUGGCUGUGCUGCUCC |
| 002108 | hsa-miR-30c-1# | hsa-miR-30c-1-3p::mmu-miR-30c-1-3p::rno-miR-30c-1-3p | MIMAT0004616::MIMAT0004674::MIMAT0004719 | hsa-miR-30c-1*(17)::mmu-miR-30c-1*(17)::rno-miR-30c-1*(18) | CUGGGAGAGGGUUGUUUACUCC |
| 002109 | hsa-miR-32 | aca-miR-32-5p::eca-miR-32::hsa-miR-32-5p::mmu-miR-32-5p::rno-miR-32-5p | MIMAT0000090::MIMAT0000654::MIMAT0000811::MIMAT0013180::MIMAT0021932 | aca-miR-32(18)::hsa-miR-32(17)::mmu-miR-32(17)::rno-miR-32(18) | UAUUGCACAUUACUAAGUUGCA |
| 002110 | hsa-miR-30c-2# | aca-miR-30c-3p::hsa-miR-30c-2-3p::mmu-miR-30c-2-3p::oan-miR-30c-3p::rno-miR-30c-2-3p::ssc-miR-30c-3p::tgu-miR-30c-3p | MIMAT0004550::MIMAT0005438::MIMAT0005442::MIMAT0006912::MIMAT0014686::MIMAT0021925::MIMAT0022922 | aca-miR-30c*(18)::hsa-miR-30c-2*(17)::mmu-miR-30c-2*(17)::oan-miR-30c*(18)::rno-miR-30c-2*(18) | CUGGGAGAAGGCUGUUUACUCU |
| 002111 | hsa-miR-32# | hsa-miR-32-3p | MIMAT0004505 | hsa-miR-32*(17) | CAAUUUAGUGUGUGUGAUAUUU |
| 002112 | hsa-miR-29a | cfa-miR-29a::eca-miR-29a::hsa-miR-29a-3p::mmu-miR-29a-3p::rno-miR-29a-3p | MIMAT0000086::MIMAT0000535::MIMAT0000802::MIMAT0006626::MIMAT0012940 | hsa-miR-29a(17)::mmu-miR-29a(17)::rno-miR-29a(18) | UAGCACCAUCUGAAAUCGGUUA |
| 002113 | hsa-miR-31# | hsa-miR-31-3p | MIMAT0004504 | hsa-miR-31*(17) | UGCUAUGCCAACAUAUUGCCAU |
| 002114 | hsa-miR-130b# | hsa-miR-130b-5p | MIMAT0004680 | hsa-miR-130b*(17) | ACUCUUUCCCUGUUGCACUAC |
| 002115 | hsa-miR-26a-2# | hsa-miR-26a-2-3p | MIMAT0004681 | hsa-miR-26a-2*(17) | CCUAUUCUUGAUUACUUGUUUC |
| 002116 | hsa-miR-361-3p | hsa-miR-361-3p::mml-miR-361-3p::ppy-miR-361-3p::ptr-miR-361 | MIMAT0004682::MIMAT0006287::MIMAT0008115::MIMAT0015849 | | UCCCCCAGGUGUGAUUCUGAUUU |
| 002117 | hsa-miR-362-3p | eca-miR-362-3p::ggo-miR-362::hsa-miR-362-3p::mml-miR-362-3p::ppy-miR-362-3p::ptr-miR-362 | MIMAT0004683::MIMAT0006289::MIMAT0008116::MIMAT0013211::MIMAT0015851::MIMAT0024241 | | AACACACCUAUUCAAGGAUUCA |
| 002118 | hsa-let-7g# | cgr-let-7g-3p::hsa-let-7g-3p | MIMAT0004584::MIMAT0023723 | hsa-let-7g*(17) | CUGUACAGGCCACUGCCUUGC |
| 002119 | hsa-miR-302b# | hsa-miR-302b-5p | MIMAT0000714 | hsa-miR-302b*(17) | ACUUUAACAUGGAAGUGCUUUC |
| 002120 | hsa-miR-302d# | cfa-miR-302d::hsa-miR-302d-5p | MIMAT0004685::MIMAT0009858 | hsa-miR-302d*(17) | ACUUUAACAUGGAGGCACUUGC |
| 002121 | hsa-miR-367# | cfa-miR-367::hsa-miR-367-5p | MIMAT0004686::MIMAT0009859 | hsa-miR-367*(17) | ACUGUUGCUAAUAUGCAACUCU |
| 002122 | hsa-miR-376c | eca-miR-376c::hsa-miR-376c-3p::mml-miR-376c::oar-miR-376c-3p::ppy-miR-376c::ptr-miR-376c | MIMAT0000720::MIMAT0006305::MIMAT0008128::MIMAT0013145::MIMAT0015866::MIMAT0019280 | hsa-miR-376c(18) | AACAUAGAGGAAAUUCCACGU |
| 002124 | hsa-miR-371-3p | hsa-miR-371a-3p::ppy-miR-371-3p::ptr-miR-371 | MIMAT0000723::MIMAT0008121::MIMAT0015859 | hsa-miR-371-3p(17) | AAGUGCCGCCAUCUUUUGAGUGU |
| 002125 | hsa-miR-374a# | hsa-miR-374a-3p | MIMAT0004688 | hsa-miR-374a*(17) | CUUAUCAGAUUGUAUUGUAAUU |
| 002126 | hsa-miR-23b# | hsa-miR-23b-5p::oan-miR-23b-5p | MIMAT0004587::MIMAT0006958 | hsa-miR-23b*(17)::oan-miR-23b*(18) | UGGGUUCCUGGCAUGCUGAUUU |
| 002127 | hsa-miR-376a# | hsa-miR-376a-5p | MIMAT0003386 | hsa-miR-376a*(17) | GUAGAUUCUCCUUCUAUGAGUA |
| 002128 | hsa-miR-377# | cfa-miR-377::hsa-miR-377-5p::mmu-miR-377-5p::rno-miR-377-5p | MIMAT0004689::MIMAT0009890::MIMAT0017079::MIMAT0017203 | hsa-miR-377*(17)::mmu-miR-377*(17)::rno-miR-377*(18) | AGAGGUUGCCCUUGGUGAAUUC |
| 002129 | hsa-miR-30b# | hsa-miR-30b-3p | MIMAT0004589 | hsa-miR-30b*(17) | CUGGGAGGUGGAUGUUUACUUC |
| 002130 | hsa-miR-122# | aca-miR-122-3p::hsa-miR-122-3p | MIMAT0004590::MIMAT0021724 | aca-miR-122*(18)::hsa-miR-122*(17) | AACGCCAUUAUCACACUAAAUA |
| 002131 | hsa-miR-130a# | hsa-miR-130a-5p | MIMAT0004593 | hsa-miR-130a*(17) | UUCACAUUGUGCUACUGUCUGC |
| 002132 | hsa-miR-132# | cgr-miR-132-5p::hsa-miR-132-5p::rno-miR-132-5p | MIMAT0004594::MIMAT0017123::MIMAT0023758 | hsa-miR-132*(17)::rno-miR-132*(18) | ACCGUGGCUUUCGAUUGUUACU |
| 002134 | hsa-miR-148a# | hsa-miR-148a-5p::mmu-miR-148a-5p::ssc-miR-148a-5p | MIMAT0004549::MIMAT0004617::MIMAT0022920 | hsa-miR-148a*(17)::mmu-miR-148a*(17) | AAAGUUCUGAGACACUCCGACU |
| 002135 | hsa-miR-33a | aae-miR-33::aca-miR-33-5p::bmo-miR-33-5p::bta-miR-33a::cqu-miR-33::cte-miR-33::eca-miR-33a::hsa-miR-33a-5p::mdo-miR-33::mmu-miR-33-5p::mse-miR-33::rno-miR-33-5p | MIMAT0000091::MIMAT0000667::MIMAT0000812::MIMAT0009294::MIMAT0009553::MIMAT0012754::MIMAT0013189::MIMAT0013599::MIMAT0014287::MIMAT0014431::MIMAT0021934::MIMAT0024429 | aca-miR-33(18)::bmo-miR-33(18)::cap-miR-33(14)::hsa-miR-33a(17)::mmu-miR-33(17)::rno-miR-33(18) | GUGCAUUGUAGUUGCAUUGCA |
| 002136 | hsa-miR-33a# | hsa-miR-33a-3p::mmu-miR-33-3p | MIMAT0004506::MIMAT0004666 | hsa-miR-33a*(17)::mmu-miR-33*(17) | CAAUGUUUCCACAGUGCAUCAC |
| 002137 | hsa-miR-92a-1# | hsa-miR-92a-1-5p | MIMAT0004507 | hsa-miR-92a-1*(17) | AGGUUGGGAUCGGUUGCAAUGCU |
| 002138 | hsa-miR-92a-2# | hsa-miR-92a-2-5p | MIMAT0004508 | hsa-miR-92a-2*(17) | GGGUGGGGAUUUGUUGCAUUAC |
| 002139 | hsa-miR-93# | hsa-miR-93-3p::mmu-miR-93-3p | MIMAT0004509::MIMAT0004636 | hsa-miR-93*(17)::mmu-miR-93*(17) | ACUGCUGAGCUAGCACUUCCCG |
| 002140 | hsa-miR-96# | hsa-miR-96-3p | MIMAT0004510 | hsa-miR-96*(17) | AAUCAUGUGCAGUGCCAAUAUG |
| 002141 | hsa-miR-99a# | cgr-miR-99a-3p::hsa-miR-99a-3p | MIMAT0004511::MIMAT0024017 | hsa-miR-99a*(17) | CAAGCUCGCUUCUAUGGGUCUG |
| 002142 | hsa-miR-100# | hsa-miR-100-3p | MIMAT0004512 | hsa-miR-100*(17) | CAAGCUUGUAUCUAUAGGUAUG |
| 002143 | hsa-miR-101# | hsa-miR-101-5p | MIMAT0004513 | hsa-miR-101*(17) | CAGUUAUCACAGUGCUGAUGCU |
| 002144 | hsa-miR-138-2# | hsa-miR-138-2-3p | MIMAT0004596 | hsa-miR-138-2*(17) | GCUAUUUCACGACACCAGGGUU |
| 002145 | hsa-miR-141# | hsa-miR-141-5p | MIMAT0004598 | hsa-miR-141*(17) | CAUCUUCCAGUACAGUGUUGGA |
| 002146 | hsa-miR-143# | hsa-miR-143-5p | MIMAT0004599 | hsa-miR-143*(17) | GGUGCAGUGCUGCAUCUCUGGU |
| 002147 | hsa-miR-342-5p | hsa-miR-342-5p::mml-miR-342-5p::ppy-miR-342-5p | MIMAT0004694::MIMAT0006282::MIMAT0015844 | | AGGGGUGCUAUCUGUGAUUGA |
| 002148 | hsa-miR-144# | cgr-miR-144::ggo-miR-144::hsa-miR-144-5p | MIMAT0004600::MIMAT0023773::MIMAT0024219 | hsa-miR-144*(17) | GGAUAUCAUCAUAUACUGUAAG |
| 002149 | hsa-miR-145# | hsa-miR-145-3p::ssc-miR-145-3p | MIMAT0004601::MIMAT0022919 | hsa-miR-145*(17) | GGAUUCCUGGAAAUACUGUUCU |
| 002150 | hsa-miR-920 | hsa-miR-920::ptr-miR-920 | MIMAT0004970::MIMAT0008336 | | GGGGAGCUGUGGAAGCAGUA |
| 002151 | hsa-miR-921 | hsa-miR-921 | MIMAT0004971 |  | CUAGUGAGGGACAGAACCAGGAUUC |
| 002152 | hsa-miR-922 | hsa-miR-922 | MIMAT0004972 |  | GCAGCAGAGAAUAGGACUACGUC |
| 002154 | hsa-miR-924 | hsa-miR-924::ppy-miR-924::ptr-miR-924 | MIMAT0004974::MIMAT0008339::MIMAT0016133 | | AGAGUCUUGUGAUGUCUUGC |
| 002155 | hsa-miR-509-3-5p | ggo-miR-509::hsa-miR-509-3-5p | MIMAT0004975::MIMAT0024234 | | UACUGCAGACGUGGCAAUCAUG |
| 002156 | hsa-miR-337-5p | hsa-miR-337-5p::mml-miR-337-5p::ppy-miR-337-5p | MIMAT0004695::MIMAT0006275::MIMAT0015838 | | GAACGGCUUCAUACAGGAGUU |
| 002157 | hsa-miR-337-3p | hsa-miR-337-3p::mml-miR-337-3p::ppy-miR-337-3p::ptr-miR-337 | MIMAT0000754::MIMAT0006276::MIMAT0008105::MIMAT0015839 | | CUCCUAUAUGAUGCCUUUCUUC |
| 002158 | hsa-miR-125b-2# | cgr-miR-125b-3p::hsa-miR-125b-2-3p | MIMAT0004603::MIMAT0023744 | hsa-miR-125b-2*(17) | UCACAAGUCAGGCUCUUGGGAC |
| 002159 | hsa-miR-135b# | hsa-miR-135b-3p::mmu-miR-135b-3p::rno-miR-135b-3p | MIMAT0004698::MIMAT0017043::MIMAT0017044 | hsa-miR-135b*(17)::mmu-miR-135b*(17)::rno-miR-135b*(18) | AUGUAGGGCUAAAAGCCAUGGG |
| 002160 | hsa-miR-148b# | hsa-miR-148b-5p | MIMAT0004699 | hsa-miR-148b*(17) | AAGUUCUGUUAUACACUCAGGC |
| 002161 | hsa-miR-324-3p | hsa-miR-324-3p::mml-miR-324-3p::ppy-miR-324-3p::ptr-miR-324 | MIMAT0000762::MIMAT0006267::MIMAT0008098::MIMAT0015828 | | ACUGCCCCAGGUGCUGCUGG |
| 002163 | hsa-miR-146a# | hsa-miR-146a-3p | MIMAT0004608 | hsa-miR-146a*(17) | CCUCUGAAAUUCAGUUCUUCAG |
| 002164 | hsa-miR-149# | hsa-miR-149-3p | MIMAT0004609 | hsa-miR-149*(17) | AGGGAGGGACGGGGGCUGUGC |
| 002165 | hsa-miR-29b-1# | cgr-miR-29b-5p::hsa-miR-29b-1-5p | MIMAT0004514::MIMAT0023885 | hsa-miR-29b-1*(17) | GCUGGUUUCAUAUGGUGGUUUAGA |
| 002166 | hsa-miR-29b-2# | hsa-miR-29b-2-5p::rno-miR-29b-2-5p | MIMAT0004515::MIMAT0004717 | hsa-miR-29b-2*(17)::rno-miR-29b-2*(18) | CUGGUUUCACAUGGUGGCUUAG |
| 002167 | hsa-miR-105 | bta-miR-105a::eca-miR-105::hsa-miR-105-5p::mml-miR-105::ppy-miR-105 | MIMAT0000102::MIMAT0002450::MIMAT0002454::MIMAT0009217::MIMAT0013193 | hsa-miR-105(17) | UCAAAUGCUCAGACUCCUGUGGU |
| 002168 | hsa-miR-105# | hsa-miR-105-3p | MIMAT0004516 | hsa-miR-105*(17) | ACGGAUGUUUGAGCAUGUGCUA |
| 002169 | hsa-miR-106a | hsa-miR-106a-5p::tgu-miR-106 | MIMAT0000103::MIMAT0014590 | hsa-miR-106a(17) | AAAAGUGCUUACAGUGCAGGUAG |
| 002170 | hsa-miR-106a# | hsa-miR-106a-3p | MIMAT0004517 | hsa-miR-106a*(17) | CUGCAAUGUAAGCACUUCUUAC |
| 002171 | hsa-miR-16-2# | hsa-miR-16-2-3p | MIMAT0004518 | hsa-miR-16-2*(17) | CCAAUAUUACUGUGCUGCUUUA |
| 002172 | hsa-let-7i# | hsa-let-7i-3p::mmu-let-7i-3p::rno-let-7i-3p | MIMAT0004520::MIMAT0004585::MIMAT0004707 | hsa-let-7i*(17)::mmu-let-7i*(17)::rno-let-7i*(18) | CUGCGCAAGCUACUGCCUUGCU |
| 002173 | hsa-miR-15b# | hsa-miR-15b-3p::mmu-miR-15b-3p::rno-miR-15b-3p | MIMAT0004521::MIMAT0004586::MIMAT0017093 | hsa-miR-15b*(17)::mmu-miR-15b*(17)::rno-miR-15b*(18) | CGAAUCAUUAUUUGCUGCUCUA |
| 002174 | hsa-miR-27b# | aca-miR-27b-5p::cgr-miR-27b-5p::hsa-miR-27b-5p::mmu-miR-27b-5p::oan-miR-27b-5p | MIMAT0004522::MIMAT0004588::MIMAT0006956::MIMAT0021907::MIMAT0023876 | aca-miR-27b*(18)::hsa-miR-27b*(17)::mmu-miR-27b*(17)::oan-miR-27b*(18) | AGAGCUUAGCUGAUUGGUGAAC |
| 002176 | hsa-miR-933 | hsa-miR-933::mml-miR-933::ppy-miR-933::ptr-miR-933 | MIMAT0004976::MIMAT0006535::MIMAT0008340::MIMAT0016134 | | UGUGCGCAGGGAGACCUCUCCC |
| 002177 | hsa-miR-934 | hsa-miR-934::ppy-miR-934::ptr-miR-934 | MIMAT0004977::MIMAT0008341::MIMAT0016135 | | UGUCUACUACUGGAGACACUGG |
| 002178 | hsa-miR-935 | bta-miR-935::hsa-miR-935::ptr-miR-935::rno-miR-935::ssc-miR-935 | MIMAT0004978::MIMAT0008342::MIMAT0009385::MIMAT0012845::MIMAT0013947 | | CCAGUUACCGCUUCCGCUACCGC |
| 002179 | hsa-miR-936 | hsa-miR-936::ptr-miR-936 | MIMAT0004979::MIMAT0008343 | | ACAGUAGAGGGAGGAAUCGCAG |
| 002180 | hsa-miR-937 | hsa-miR-937-3p::ptr-miR-937 | MIMAT0004980::MIMAT0008344 | hsa-miR-937(18) | AUCCGCGCUCUGACUCUCUGCC |
| 002181 | hsa-miR-938 | hsa-miR-938::ppy-miR-938::ptr-miR-938 | MIMAT0004981::MIMAT0008345::MIMAT0016138 | | UGCCCUUAAAGGUGAACCCAGU |
| 002182 | hsa-miR-939 | hsa-miR-939-5p::mml-miR-939::ptr-miR-939 | MIMAT0004982::MIMAT0006540::MIMAT0008346 | hsa-miR-939(18) | UGGGGAGCUGAGGCUCUGGGGGUG |
| 002183 | hsa-miR-941 | hsa-miR-941 | MIMAT0004984 |  | CACCCGGCUGUGUGCACAUGUGC |
| 002184 | hsa-miR-339-3p | ggo-miR-339::hsa-miR-339-3p::mml-miR-339-3p | MIMAT0004702::MIMAT0006280::MIMAT0024161 | | UGAGCGCCUCGACGACAGAGCCG |
| 002185 | hsa-miR-335# | hsa-miR-335-3p::mmu-miR-335-3p | MIMAT0004703::MIMAT0004704 | hsa-miR-335*(17) | UUUUUCAUUAUUGCUCCUGACC |
| 002186 | hsa-miR-345 | hsa-miR-345-5p::ppy-miR-345::ptr-miR-345 | MIMAT0000772::MIMAT0008111::MIMAT0015846 | hsa-miR-345(17) | GCUGACUCCUAGUCCAGGGCUC |
| 002187 | hsa-miR-942 | hsa-miR-942::ptr-miR-942 | MIMAT0004985::MIMAT0008348 | | UCUUCUCUGUUUUGGCCAUGUG |
| 002188 | hsa-miR-943 | hsa-miR-943::ptr-miR-943 | MIMAT0004986::MIMAT0008349 | | CUGACUGUUGCCGUCCUCCAG |
| 002189 | hsa-miR-944 | hsa-miR-944::ptr-miR-944 | MIMAT0004987::MIMAT0008350 | | AAAUUAUUGUACAUCGGAUGAG |
| 002190 | hsa-miR-298 | hsa-miR-298::ptr-miR-298 | MIMAT0004901::MIMAT0008081 | | AGCAGAAGCAGGGAGGUUCUCCCA |
| 002191 | hsa-miR-891a | hsa-miR-891a | MIMAT0004902 |  | UGCAACGAACCUGAGCCACUGA |
| 002193 | hsa-miR-886-5p |  |  | hsa-miR-886-5p(15)::mml-miR-886-5p(15)::ppy-miR-886-5p(15) | CGGGUCGGAGUUAGCUCAAGCGG |
| 002194 | hsa-miR-886-3p |  |  | hsa-miR-886-3p(15)::mml-miR-886-3p(15)::ppy-miR-886-3p(15)::ptr-miR-886(15) | CGCGGGUGCUUACUGACCCUU |
| 002195 | hsa-miR-892a | hsa-miR-892a::mml-miR-892a::ptr-miR-892a | MIMAT0004907::MIMAT0006531::MIMAT0008335 | mml-miR-892(18) | CACUGUGUCCUUUCUGCGUAG |
| 002196 | hsa-miR-99b# | hsa-miR-99b-3p::mmu-miR-99b-3p::rno-miR-99b-3p | MIMAT0004525::MIMAT0004678::MIMAT0004725 | hsa-miR-99b*(17)::mmu-miR-99b*(17)::rno-miR-99b*(18) | CAAGCUCGUGUCUGUGGGUCCG |
| 002197 | hsa-miR-124# | hsa-miR-124-5p::mmu-miR-124-5p::oan-miR-124-5p::pma-miR-124-5p::pol-miR-124-5p::rno-miR-124-5p | MIMAT0004527::MIMAT0004591::MIMAT0004728::MIMAT0007113::MIMAT0019444::MIMAT0025430 | hsa-miR-124*(17)::mmu-miR-124*(17)::oan-miR-124*(18)::pma-miR-124*(18)::rno-miR-124*(18) | CGUGUUCACAGCGGACCUUGAU |
| 002198 | hsa-miR-125a-5p | cgr-miR-125a-5p::eca-miR-125a-5p::hsa-miR-125a-5p::mml-miR-125a-5p::mmu-miR-125a-5p::ppy-miR-125a-5p::rno-miR-125a-5p | MIMAT0000135::MIMAT0000443::MIMAT0000829::MIMAT0006181::MIMAT0013008::MIMAT0015748::MIMAT0023741 | | UCCCUGAGACCCUUUAACCUGUGA |
| 002199 | hsa-miR-125a-3p | eca-miR-125a-3p::hsa-miR-125a-3p::mml-miR-125a-3p::mmu-miR-125a-3p::ppy-miR-125a-3p::ptr-miR-125a::rno-miR-125a-3p | MIMAT0004528::MIMAT0004602::MIMAT0004729::MIMAT0006182::MIMAT0007984::MIMAT0013009::MIMAT0015749 | | ACAGGUGAGGUUCUUGGGAGCC |
| 002200 | hsa-miR-541# | hsa-miR-541-5p::mml-miR-541::oar-miR-541-5p | MIMAT0004919::MIMAT0012784::MIMAT0019323 | hsa-miR-541*(17) | AAAGGAUUCUGCUGUCGGUCCCACU |
| 002201 | hsa-miR-541 | hsa-miR-541-3p | MIMAT0004920 | hsa-miR-541(17) | UGGUGGGCACAGAAUCUGGACU |
| 002202 | hsa-miR-889 | eca-miR-889::hsa-miR-889::mml-miR-889::ppy-miR-889::ptr-miR-889 | MIMAT0004921::MIMAT0006528::MIMAT0008331::MIMAT0013175::MIMAT0016124 | | UUAAUAUCGGACAACCAUUGU |
| 002203 | hsa-miR-875-5p | bta-miR-875::cfa-miR-875::hsa-miR-875-5p::mml-miR-875-5p::mmu-miR-875-5p::ppy-miR-875-5p::ptr-miR-875::rno-miR-875 | MIMAT0004922::MIMAT0004937::MIMAT0006517::MIMAT0008326::MIMAT0009379::MIMAT0009931::MIMAT0012842::MIMAT0016113 | | UAUACCUCAGUUUUAUCAGGUG |
| 002204 | hsa-miR-875-3p | hsa-miR-875-3p | MIMAT0004923 |  | CCUGGAAACACUGAGGUUGUG |
| 002205 | hsa-miR-876-5p | bta-miR-876::cfa-miR-876::eca-miR-876-5p::hsa-miR-876-5p::mml-miR-876-5p::ppy-miR-876-5p | MIMAT0004924::MIMAT0006519::MIMAT0009380::MIMAT0009932::MIMAT0013121::MIMAT0016115 | | UGGAUUUCUUUGUGAAUCACCA |
| 002206 | hsa-miR-220b |  |  | hsa-miR-220b(15)::ppy-miR-220b(15)::ptr-miR-220b(15) | CCACCACCGUGUCUGACACUU |
| 002207 | hsa-miR-450b-5p | bta-miR-450b::eca-miR-450b-5p::ggo-miR-450b::hsa-miR-450b-5p::mml-miR-450b-5p::ppy-miR-450b-5p::ssc-miR-450b-5p | MIMAT0004909::MIMAT0006333::MIMAT0013220::MIMAT0013927::MIMAT0015894::MIMAT0024296::MIMAT0024576 | ssc-miR-450b(15) | UUUUGCAAUAUGUUCCUGAAUA |
| 002208 | hsa-miR-450b-3p | hsa-miR-450b-3p::mml-miR-450b-3p::ppy-miR-450b-3p::ptr-miR-450b | MIMAT0004910::MIMAT0006334::MIMAT0008152::MIMAT0015895 | | UUGGGAUCAUUUUGCAUCCAUA |
| 002209 | hsa-miR-890 | hsa-miR-890::ppy-miR-890::ptr-miR-890 | MIMAT0004912::MIMAT0008332::MIMAT0016125 | | UACUUGGAAAGGCAUCAGUUG |
| 002210 | hsa-miR-891b | hsa-miR-891b::mml-miR-891a::ppy-miR-891b::ptr-miR-891b | MIMAT0004913::MIMAT0006530::MIMAT0008334::MIMAT0016127 | mml-miR-891(18) | UGCAACUUACCUGAGUCAUUGA |
| 002211 | hsa-miR-220c |  |  | hsa-miR-220c(15)::ppy-miR-220c(15) | ACACAGGGCUGUUGUGAAGACU |
| 002212 | hsa-miR-888 | hsa-miR-888-5p::mml-miR-888 | MIMAT0004916::MIMAT0006527 | hsa-miR-888(17) | UACUCAAAAAGCUGUCAGUCA |
| 002213 | hsa-miR-888# | hsa-miR-888-3p | MIMAT0004917 | hsa-miR-888*(17) | GACUGACACCUCUUUGGGUGAA |
| 002214 | hsa-miR-892b | hsa-miR-892b::mml-miR-892b::ppy-miR-892b | MIMAT0004918::MIMAT0012783::MIMAT0016129 | | CACUGGCUCCUUUCUGGGUAGA |
| 002215 | hsa-miR-196b | cgr-miR-196b::eca-miR-196b::hsa-miR-196b-5p::mml-miR-196b::mmu-miR-196b-5p::ppy-miR-196b::ptr-miR-196b::rno-miR-196b-5p::ssc-miR-196b::ssc-miR-196b-5p | MIMAT0001080::MIMAT0001081::MIMAT0001082::MIMAT0006229::MIMAT0008061::MIMAT0012939::MIMAT0013923::MIMAT0013923::MIMAT0025369::MIMAT0015791::MIMAT0023833 | hsa-miR-196b(17)::mmu-miR-196b(17)::rno-miR-196b(18) | UAGGUAGUUUCCUGUUGUUGGG |
| 002216 | hsa-miR-128a | aca-miR-128-3p::bta-miR-128::ccr-miR-128::cfa-miR-128::cgr-miR-128-3p::eca-miR-128::gga-miR-128::hsa-miR-128::mdo-miR-128::mml-miR-128b::mmu-miR-128-3p::oan-miR-128-3p::ppy-miR-128::ptr-miR-128::rno-miR-128-3p::sha-miR-128::ssc-miR-128::tgu-miR-128-3p | MIMAT0000140::MIMAT0000424::MIMAT0000834::MIMAT0001123::MIMAT0002157::MIMAT0002230::MIMAT0002231::MIMAT0003541::MIMAT0004104::MIMAT0006184::MIMAT0006633::MIMAT0007006::MIMAT0013076::MIMAT0014513::MIMAT0021733::MIMAT0022798::MIMAT0023750::MIMAT0026208 | aca-miR-128(18)::gga-miR-128b(5)::hsa-miR-128a(10)::hsa-miR-128b(10)::mdo-miR-128b(10)::mmu-miR-128(17)::mmu-miR-128a(10)::mmu-miR-128b(10)::oan-miR-128(18)::rno-miR-128(18)::rno-miR-128a(10)::rno-miR-128b(10)::tgu-miR-128(18) | UCACAGUGAACCGGUCUCUUU |
| 002217 | hsa-miR-18b | aca-miR-18b-5p::eca-miR-18b::hsa-miR-18b-5p::mml-miR-18b::ppy-miR-18b::ptr-miR-18b::ssc-miR-18b::xla-miR-18::xtr-miR-18b | MIMAT0001349::MIMAT0001412::MIMAT0003706::MIMAT0006163::MIMAT0008054::MIMAT0013200::MIMAT0015731::MIMAT0020585::MIMAT0021819 | aca-miR-18b(18)::hsa-miR-18b(17) | UAAGGUGCAUCUAGUGCAGUUAG |
| 002218 | hsa-miR-10b | bta-miR-10b::dre-miR-10b::eca-miR-10b::fru-miR-10b::hsa-miR-10b-5p::mml-miR-10b::mmu-miR-10b-5p::ppy-miR-10b::ptr-miR-10b::tni-miR-10b | MIMAT0000208::MIMAT0000254::MIMAT0001268::MIMAT0002963::MIMAT0002964::MIMAT0003839::MIMAT0006162::MIMAT0007945::MIMAT0013090::MIMAT0015730 | hsa-miR-10b(17)::mmu-miR-10b(17) | UACCCUGUAGAACCGAAUUUGUG |
| 002220 | hsa-miR-216a | aca-miR-216a::bta-miR-216a::dre-miR-216a::eca-miR-216a::hsa-miR-216a-5p::mml-miR-216a::mmu-miR-216a-5p::oan-miR-216-5p::rno-miR-216a-5p::tgu-miR-216a | MIMAT0000273::MIMAT0000662::MIMAT0000886::MIMAT0001284::MIMAT0006242::MIMAT0007223::MIMAT0009265::MIMAT0013072::MIMAT0014576::MIMAT0021875 | hsa-miR-216a(18)::mmu-miR-216a(17)::oan-miR-216(18)::rno-miR-216a(18) | UAAUCUCAGCUGGCAACUGUGA |
| 002222 | hsa-miR-1 | aca-miR-1a-3p::bfl-miR-1-3p::bta-miR-1::ccr-miR-1::dre-miR-1::eca-miR-1::fru-miR-1::hsa-miR-1::lgi-miR-1::mml-miR-1::mmu-miR-1a-3p::oan-miR-1a::pol-miR-1-3p::ppy-miR-1::ptr-miR-1::spu-miR-1::tgu-miR-1::tni-miR-1 | MIMAT0000123::MIMAT0000416::MIMAT0001768::MIMAT0003071::MIMAT0003072::MIMAT0006150::MIMAT0006863::MIMAT0007946::MIMAT0009214::MIMAT0009463::MIMAT0009557::MIMAT0009650::MIMAT0012994::MIMAT0014562::MIMAT0015720::MIMAT0021840::MIMAT0025413::MIMAT0026194 | aca-miR-1a(18)::bfl-miR-1(18)::mmu-miR-1(16)::mmu-miR-1a(17) | UGGAAUGUAAAGAAGUAUGUAU |
| 002225 | hsa-miR-876-3p | hsa-miR-876-3p::mml-miR-876-3p::ppy-miR-876-3p::ptr-miR-876 | MIMAT0004925::MIMAT0006520::MIMAT0008327::MIMAT0016116 | | UGGUGGUUUACAAAGUAAUUCA |
| 002227 | hsa-miR-323-3p | cfa-miR-323::eca-miR-323-3p::hsa-miR-323a-3p::mml-miR-323-3p::mmu-miR-323-3p::oar-miR-323a-3p::ppy-miR-323-3p::ptr-miR-323::rno-miR-323-3p | MIMAT0000550::MIMAT0000551::MIMAT0000755::MIMAT0006265::MIMAT0006718::MIMAT0008097::MIMAT0013132::MIMAT0015826::MIMAT0019260 | hsa-miR-323-3p(17)::rno-miR-323(18) | CACAUUACACGGUCGACCUCU |
| 002228 | hsa-miR-126 | aca-miR-126-3p::cgr-miR-126::eca-miR-126-3p::hsa-miR-126-3p::mml-miR-126::mmu-miR-126-3p::oan-miR-126-3p::ppy-miR-126::ptr-miR-126::rno-miR-126a-3p::ssc-miR-126-3p::tgu-miR-126-3p | MIMAT0000138::MIMAT0000445::MIMAT0000832::MIMAT0006183::MIMAT0007126::MIMAT0007985::MIMAT0013177::MIMAT0014544::MIMAT0015750::MIMAT0018378::MIMAT0021731::MIMAT0023745 | aca-miR-126(18)::hsa-miR-126(17)::oan-miR-126(18)::rno-miR-126(18)::ssc-miR-126(18)::tgu-miR-126(18) | UCGUACCGUGAGUAAUAAUGCG |
| 002229 | hsa-miR-127-5p | hsa-miR-127-5p::mmu-miR-127-5p | MIMAT0004530::MIMAT0004604 | mmu-miR-127*(17) | CUGAAGCUCAGAGGGCUCUGAU |
| 002230 | hsa-miR-330-5p | cfa-miR-330::eca-miR-330::hsa-miR-330-5p::mml-miR-330-5p::mmu-miR-330-5p::ppy-miR-330-5p::rno-miR-330-5p | MIMAT0004641::MIMAT0004642::MIMAT0004693::MIMAT0006270::MIMAT0009893::MIMAT0013012::MIMAT0015833 | mmu-miR-330(17)::rno-miR-330(18) | UCUCUGGGCCUGUGUCUUAGGC |
| 002231 | hsa-miR-9# | hsa-miR-9-3p::mmu-miR-9-3p::rno-miR-9a-3p::tgu-miR-9-3p | MIMAT0000143::MIMAT0000442::MIMAT0004708::MIMAT0014627 | hsa-miR-9*(17)::mmu-miR-9*(17)::rno-miR-9*(18)::tgu-miR-9*(18) | AUAAAGCUAGAUAACCGAAAGU |
| 002233 | hsa-miR-331-5p | hsa-miR-331-5p::mml-miR-331-5p::mmu-miR-331-5p::ppy-miR-331-5p | MIMAT0004643::MIMAT0004700::MIMAT0006272::MIMAT0015835 | | CUAGGUAUGGUCCCAGGGAUCC |
| 002234 | hsa-miR-140-3p | aca-miR-140-3p::eca-miR-140-3p::hsa-miR-140-3p::mml-miR-140-3p::mmu-miR-140-3p::ppy-miR-140-3p::rno-miR-140-3p | MIMAT0000152::MIMAT0000574::MIMAT0004597::MIMAT0006198::MIMAT0012927::MIMAT0015764::MIMAT0021766 | mmu-miR-140*(17)::rno-miR-140*(18) | UACCACAGGGUAGAACCACGG |
| 002235 | hsa-miR-509-5p | eca-miR-509-5p::hsa-miR-509-5p::ppy-miR-509-5p | MIMAT0004779::MIMAT0013233::MIMAT0015933 | | UACUGCAGACAGUGGCAAUCA |
| 002237 | hsa-miR-548d-5p | hsa-miR-548d-5p | MIMAT0004812 |  | AAAAGUAAUUGUGGUUUUUGCC |
| 002238 | hsa-miR-411# | hsa-miR-411-3p::mmu-miR-411-3p | MIMAT0001093::MIMAT0004813 | hsa-miR-411*(17)::mmu-miR-411*(17) | UAUGUAACACGGUCCACUAACC |
| 002239 | hsa-miR-654-3p | bta-miR-654::hsa-miR-654-3p::mml-miR-654-3p::mmu-miR-654-3p::ppy-miR-654-3p::ptr-miR-654 | MIMAT0004814::MIMAT0004898::MIMAT0006496::MIMAT0008304::MIMAT0009359::MIMAT0016088 | | UAUGUCUGCUGACCAUCACCUU |
| 002240 | hsa-miR-542-5p | hsa-miR-542-5p::mml-miR-542-5p::ppy-miR-542-5p | MIMAT0003340::MIMAT0006406::MIMAT0015991 | | UCGGGGAUCAUCAUGUCACGAGA |
| 002241 | hsa-miR-510 | hsa-miR-510 | MIMAT0002882 |  | UACUCAGGAGAGUGGCAAUCAC |
| 002243 | hsa-miR-378 | eca-miR-378::hsa-miR-378a-3p::mml-miR-378a::mmu-miR-378a-3p::ptr-miR-378a::rno-miR-378a-3p | MIMAT0000732::MIMAT0003151::MIMAT0003379::MIMAT0006307::MIMAT0008130::MIMAT0013068 | hsa-miR-378(17)::mml-miR-378(18)::mmu-miR-378(17)::mmu-miR-378-3p(18)::ptr-miR-378(18)::rno-miR-378(18) | ACUGGACUUGGAGUCAGAAGG |
| 002244 | hsa-miR-455-3p | aca-miR-455-3p::ggo-miR-455::hsa-miR-455-3p::mml-miR-455-3p::ppy-miR-455-3p::ptr-miR-455::ssc-miR-455-3p | MIMAT0004784::MIMAT0006340::MIMAT0008157::MIMAT0013960::MIMAT0015900::MIMAT0021965::MIMAT0024222 | ssc-miR-455(18) | GCAGUCCAUGGGCAUAUACAC |
| 002245 | hsa-miR-122 | aca-miR-122-5p::bta-miR-122::cfa-miR-122::dre-miR-122::eca-miR-122::fru-miR-122::ggo-miR-122::hsa-miR-122-5p::mml-miR-122a::mmu-miR-122-5p::oan-miR-122-5p::pol-miR-122-5p::ppy-miR-122::ptr-miR-122::rno-miR-122-5p::tgu-miR-122::tni-miR-122 | MIMAT0000246::MIMAT0000421::MIMAT0000827::MIMAT0001818::MIMAT0002991::MIMAT0002992::MIMAT0003849::MIMAT0006180::MIMAT0006619::MIMAT0006825::MIMAT0007963::MIMAT0012995::MIMAT0014529::MIMAT0015746::MIMAT0021723::MIMAT0024179::MIMAT0025428 | aca-miR-122(18)::bta-miR-122a(9.2)::hsa-miR-122(17)::mmu-miR-122(17)::oan-miR-122(18)::rno-miR-122(18) | UGGAGUGUGACAAUGGUGUUUG |
| 002246 | hsa-miR-133a | aca-miR-133a::bta-miR-133a::dre-miR-133a-3p::eca-miR-133a::fru-miR-133::hsa-miR-133a::mml-miR-133c::mmu-miR-133a-3p::ppy-miR-133c::ptr-miR-133a::rno-miR-133a-3p::tni-miR-133 | MIMAT0000145::MIMAT0000427::MIMAT0000839::MIMAT0001830::MIMAT0002243::MIMAT0003057::MIMAT0003058::MIMAT0006777::MIMAT0009225::MIMAT0012997::MIMAT0016973::MIMAT0021749 | dre-miR-133a(18)::mmu-miR-133a(17)::rno-miR-133a(18) | UUUGGUCCCCUUCAACCAGCUG |
| 002247 | hsa-miR-133b | bta-miR-133b::cfa-miR-133b::dre-miR-133b-3p::eca-miR-133b::hsa-miR-133b::mml-miR-133b::mmu-miR-133b-3p::oan-miR-133b-3p::ppy-miR-133b::ptr-miR-133b::rno-miR-133b-3p | MIMAT0000769::MIMAT0000770::MIMAT0001831::MIMAT0003126::MIMAT0006189::MIMAT0006997::MIMAT0008030::MIMAT0009226::MIMAT0009835::MIMAT0013097::MIMAT0015756 | dre-miR-133b(18)::mmu-miR-133b(17)::oan-miR-133b(18)::rno-miR-133b(18) | UUUGGUCCCCUUCAACCAGCUA |
| 002248 | hsa-miR-142-5p | dre-miR-142a-5p::eca-miR-142-5p::fru-miR-142::hsa-miR-142-5p::mml-miR-142-5p::mmu-miR-142-5p::oan-miR-142-5p::ppy-miR-142-5p::rno-miR-142-5p::ssc-miR-142-5p::tni-miR-142a | MIMAT0000154::MIMAT0000433::MIMAT0000847::MIMAT0001838::MIMAT0002908::MIMAT0002909::MIMAT0006199::MIMAT0006982::MIMAT0013022::MIMAT0013919::MIMAT0015765 | fru-miR-142a(18)::oan-miR-142(18)::ssc-miR-142(16) | CAUAAAGUAGAAAGCACUACU |
| 002249 | hsa-miR-143 | aca-miR-143-3p::cfa-miR-143::cgr-miR-143::dre-miR-143::eca-miR-143::hsa-miR-143-3p::mml-miR-143::mmu-miR-143-3p::oan-miR-143-3p::ssc-miR-143-3p | MIMAT0000247::MIMAT0000435::MIMAT0001840::MIMAT0006201::MIMAT0006682::MIMAT0007144::MIMAT0013063::MIMAT0013879::MIMAT0021770::MIMAT0023772 | aca-miR-143(18)::hsa-miR-143(17)::mmu-miR-143(17)::oan-miR-143(18)::ssc-miR-143(15) | UGAGAUGAAGCACUGUAGCUC |
| 002250 | hsa-miR-193a-3p | bta-miR-193a-3p::ccr-miR-193a::dre-miR-193a::eca-miR-193a-3p::fru-miR-193::gga-miR-193a::hsa-miR-193a-3p::mml-miR-193a-3p::mmu-miR-193a-3p::ppy-miR-193a-3p::ptr-miR-193a::rno-miR-193-3p::ssc-miR-193a-3p::tni-miR-193 | MIMAT0000223::MIMAT0000459::MIMAT0000868::MIMAT0001856::MIMAT0002969::MIMAT0002970::MIMAT0003795::MIMAT0006226::MIMAT0007740::MIMAT0008058::MIMAT0013026::MIMAT0013895::MIMAT0015788::MIMAT0026251 | bta-miR-193a(13)::mmu-miR-193(17)::mmu-miR-193-3p(18)::rno-miR-193(18) | AACUGGCCUACAAAGUCCCAGU |
| 002251 | hsa-miR-200b | aca-miR-200b-3p::ccr-miR-200b::csa-miR-200::dre-miR-200b::eca-miR-200b::fru-miR-200b::hsa-miR-200b-3p::mdo-miR-200b::mmu-miR-200b-3p::tni-miR-200b | MIMAT0000233::MIMAT0000318::MIMAT0001862::MIMAT0002983::MIMAT0002984::MIMAT0004156::MIMAT0006133::MIMAT0012910::MIMAT0021847::MIMAT0026259 | aca-miR-200b(18)::hsa-miR-200b(17)::mmu-miR-200b(17) | UAAUACUGCCUGGUAAUGAUGA |
| 002252 | hsa-miR-338-3p | ccr-miR-338::dre-miR-338::eca-miR-338-3p::fru-miR-338::hsa-miR-338-3p::mml-miR-338-3p::mmu-miR-338-3p::ppy-miR-338-3p::ptr-miR-338::sha-miR-338::ssc-miR-338::tgu-miR-338-3p::tni-miR-338::xtr-miR-338 | MIMAT0000582::MIMAT0000763::MIMAT0001873::MIMAT0003007::MIMAT0003008::MIMAT0003665::MIMAT0006278::MIMAT0008106::MIMAT0013036::MIMAT0014542::MIMAT0015713::MIMAT0015841::MIMAT0022818::MIMAT0026291 | tgu-miR-338(15) | UCCAGCAUCAGUGAUUUUGUUG |
| 002253 | hsa-miR-101 | bta-miR-101::eca-miR-101::hsa-miR-101-3p::mmu-miR-101a-3p::ppy-miR-101::rno-miR-101a-3p::ssc-miR-101 | MIMAT0000099::MIMAT0000133::MIMAT0000823::MIMAT0002429::MIMAT0003520::MIMAT0010185::MIMAT0012951 | hsa-miR-101(17)::mmu-miR-101a(17)::rno-miR-101a(18)::ssc-miR-101a(14) | UACAGUACUGUGAUAACUGAA |
| 002254 | hsa-miR-151-3p | bta-miR-151-3p::hsa-miR-151a-3p::mml-miR-151-3p::ppy-miR-151a-3p::ptr-miR-151 | MIMAT0000757::MIMAT0003524::MIMAT0006213::MIMAT0008045::MIMAT0015776 | bta-miR-151(18)::hsa-miR-151-3p(17)::ppy-miR-151-3p(18) | CUAGACUGAAGCUCCUUGAGG |
| 002255 | hsa-miR-149 | bta-miR-149-5p::cfa-miR-149::eca-miR-149::ggo-miR-149::hsa-miR-149-5p::mml-miR-149::mmu-miR-149-5p::ppy-miR-149::ptr-miR-149::ssc-miR-149 | MIMAT0000159::MIMAT0000450::MIMAT0006210::MIMAT0008043::MIMAT0009884::MIMAT0012973::MIMAT0015773::MIMAT0018379::MIMAT0024144::MIMAT0024570 | hsa-miR-149(17)::mmu-miR-149(17) | UCUGGCUCCGUGUCUUCACUCCC |
| 002257 | hsa-miR-339-5p | cgr-miR-339::hsa-miR-339-5p::mml-miR-339-5p::mmu-miR-339-5p::ppy-miR-339-5p::rno-miR-339-5p | MIMAT0000583::MIMAT0000584::MIMAT0000764::MIMAT0006279::MIMAT0015842::MIMAT0023919 | | UCCCUGUCCUCCAGGAGCUCACG |
| 002258 | hsa-miR-340 | cfa-miR-340::cgr-miR-340-5p::eca-miR-340-5p::hsa-miR-340-5p::mml-miR-340::mmu-miR-340-5p::ppy-miR-340::ptr-miR-340::rno-miR-340-5p::ssc-miR-340 | MIMAT0004650::MIMAT0004651::MIMAT0004692::MIMAT0006281::MIMAT0008109::MIMAT0009892::MIMAT0013066::MIMAT0013891::MIMAT0015843::MIMAT0023920 | hsa-miR-340(17) | UUAUAAAGCAAUGAGACUGAUU |
| 002259 | hsa-miR-340# | hsa-miR-340-3p::mmu-miR-340-3p | MIMAT0000586::MIMAT0000750 | hsa-miR-340*(17) | UCCGUCUCAGUUACUUUAUAGC |
| 002260 | hsa-miR-342-3p | cfa-miR-342::eca-miR-342-3p::hsa-miR-342-3p::mml-miR-342-3p::mmu-miR-342-3p::ppy-miR-342-3p::ptr-miR-342::rno-miR-342-3p | MIMAT0000589::MIMAT0000590::MIMAT0000753::MIMAT0006283::MIMAT0006709::MIMAT0008110::MIMAT0013137::MIMAT0015845 | | UCUCACACAGAAAUCGCACCCGU |
| 002261 | hsa-miR-135b | bta-miR-135b::cfa-miR-135b::eca-miR-135b::ggo-miR-135b::hsa-miR-135b-5p::mml-miR-135b::mmu-miR-135b-5p::ppy-miR-135b::ptr-miR-135b::rno-miR-135b-5p | MIMAT0000611::MIMAT0000612::MIMAT0000758::MIMAT0006191::MIMAT0008032::MIMAT0009229::MIMAT0009839::MIMAT0012952::MIMAT0015758::MIMAT0024253 | hsa-miR-135b(17)::mmu-miR-135b(17)::rno-miR-135b(18) | UAUGGCUUUUCAUUCCUAUGUGA |
| 002262 | hsa-miR-147b | bta-miR-147::cfa-miR-147::hsa-miR-147b::mmu-miR-147-3p::ppy-miR-147b::ptr-miR-147b::rno-miR-147 | MIMAT0004857::MIMAT0004928::MIMAT0005297::MIMAT0008040::MIMAT0009237::MIMAT0009874::MIMAT0015770 | mmu-miR-147(17) | GUGUGCGGAAAUGCUUCUGCUA |
| 002263 | hsa-miR-190b | bta-miR-190b::cfa-miR-190b::eca-miR-190b::hsa-miR-190b::mdo-miR-190b::mml-miR-190b::mmu-miR-190b-5p::ppy-miR-190b::ptr-miR-190b | MIMAT0004852::MIMAT0004929::MIMAT0006222::MIMAT0008055::MIMAT0009252::MIMAT0009873::MIMAT0012756::MIMAT0012958::MIMAT0015784 | mmu-miR-190b(17) | UGAUAUGUUUGAUAUUGGGUU |
| 002264 | hsa-miR-872 | cgr-miR-872-5p::eca-miR-872::mmu-miR-872-5p::rno-miR-872-5p | MIMAT0004934::MIMAT0005282::MIMAT0013119::MIMAT0024005 | hsa-miR-872(10)::mmu-miR-872(17)::rno-miR-872(18) | AAGGUUACUUGUUAGUUCAGG |
| 002265 | hsa-miR-544 | bta-miR-544a::cfa-miR-544::eca-miR-544::hsa-miR-544a::mml-miR-544::ppy-miR-544::ptr-miR-544 | MIMAT0003164::MIMAT0006408::MIMAT0008216::MIMAT0009346::MIMAT0009910::MIMAT0013170::MIMAT0015994 | hsa-miR-544(17) | AUUCUGCAUUUUUAGCAAGUUC |
| 002266 | hsa-miR-545# | hsa-miR-545-5p | MIMAT0004785 | hsa-miR-545*(17) | UCAGUAAAUGUUUAUUAGAUGA |
| 002267 | hsa-miR-545 | hsa-miR-545-3p::mml-miR-545::ppy-miR-545::ptr-miR-545 | MIMAT0003165::MIMAT0006409::MIMAT0008217::MIMAT0015995 | hsa-miR-545(17) | UCAGCAAACAUUUAUUGUGUGC |
| 002268 | hsa-miR-874 | bta-miR-874::cfa-miR-874::eca-miR-874::ggo-miR-874::hsa-miR-874::mml-miR-874::mmu-miR-874-3p::ppy-miR-874::ptr-miR-874::rno-miR-874-3p | MIMAT0004853::MIMAT0004911::MIMAT0005284::MIMAT0006516::MIMAT0008325::MIMAT0009378::MIMAT0009930::MIMAT0013069::MIMAT0016112::MIMAT0024140 | mmu-miR-874(17)::rno-miR-874(18) | CUGCCCUGGCCCGAGGGACCGA |
| 002269 | hsa-miR-183 | bfl-miR-183::cfa-miR-183::eca-miR-183::hsa-miR-183-5p::mmu-miR-183-5p::oan-miR-183::ppy-miR-183::rno-miR-183-5p::tgu-miR-183 | MIMAT0000212::MIMAT0000261::MIMAT0000860::MIMAT0006621::MIMAT0006805::MIMAT0009487::MIMAT0012938::MIMAT0015780::MIMAT0025393 | hsa-miR-183(17)::mmu-miR-183(17)::rno-miR-183(18) | UAUGGCACUGGUAGAAUUCACU |
| 002270 | hsa-miR-183# | hsa-miR-183-3p::mmu-miR-183-3p | MIMAT0004539::MIMAT0004560 | hsa-miR-183*(17)::mmu-miR-183*(17) | GUGAAUUACCGAAGGGCCAUAA |
| 002271 | hsa-miR-185 | bta-miR-185::cfa-miR-185::cgr-miR-185-5p::ggo-miR-185::hsa-miR-185-5p::mml-miR-185::mmu-miR-185-5p::ppy-miR-185::ptr-miR-185::rno-miR-185-5p::ssc-miR-185 | MIMAT0000214::MIMAT0000455::MIMAT0000862::MIMAT0006219::MIMAT0006660::MIMAT0007759::MIMAT0008053::MIMAT0009247::MIMAT0015781::MIMAT0023811::MIMAT0024086 | hsa-miR-185(17)::mmu-miR-185(17)::rno-miR-185(18) | UGGAGAGAAAGGCAGUUCCUGA |
| 002272 | hsa-miR-192# | hsa-miR-192-3p::mmu-miR-192-3p::oan-miR-192-3p | MIMAT0004543::MIMAT0007017::MIMAT0017012 | hsa-miR-192*(17)::mmu-miR-192*(17)::oan-miR-192*(18) | CUGCCAAUUCCAUAGGUCACAG |
| 002273 | hsa-miR-198 | hsa-miR-198 | MIMAT0000228 |  | GGUCCAGAGGGGAGAUAGGUUC |
| 002274 | hsa-miR-200b# | cfa-miR-200b::hsa-miR-200b-5p::mmu-miR-200b-5p::rno-miR-200b-5p | MIMAT0004545::MIMAT0004571::MIMAT0009864::MIMAT0017152 | hsa-miR-200b*(17)::mmu-miR-200b*(17)::rno-miR-200b*(18) | CAUCUUACUGGGCAGCAUUGGA |
| 002275 | hsa-miR-370 | bta-miR-370::cfa-miR-370::eca-miR-370::hsa-miR-370::mml-miR-370::mmu-miR-370-3p::ppy-miR-370::ptr-miR-370::rno-miR-370-3p::ssc-miR-370 | MIMAT0000722::MIMAT0001095::MIMAT0003122::MIMAT0006295::MIMAT0008120::MIMAT0009300::MIMAT0009889::MIMAT0013142::MIMAT0015857::MIMAT0025373 | mmu-miR-370(17)::rno-miR-370(18) | GCCUGCUGGGGUGGAACCUGGU |
| 002276 | hsa-miR-222 | bta-miR-222::cfa-miR-222::eca-miR-222::hsa-miR-222-3p::mml-miR-222::mmu-miR-222-3p::oan-miR-222a-3p::ppy-miR-222::rno-miR-222-3p::sha-miR-222 | MIMAT0000279::MIMAT0000670::MIMAT0000891::MIMAT0003530::MIMAT0006250::MIMAT0007133::MIMAT0009851::MIMAT0013204::MIMAT0015808::MIMAT0022796 | hsa-miR-222(17)::mmu-miR-222(17)::oan-miR-222a(18)::rno-miR-222(18) | AGCUACAUCUGGCUACUGGGU |
| 002277 | hsa-miR-320 | bta-miR-320a::cfa-miR-320::cgr-miR-320a::ggo-miR-320a::hsa-miR-320a::mml-miR-320a::mmu-miR-320-3p::ppy-miR-320a::ptr-miR-320a::rno-miR-320-3p | MIMAT0000510::MIMAT0000666::MIMAT0000903::MIMAT0003534::MIMAT0006263::MIMAT0006658::MIMAT0008093::MIMAT0015821::MIMAT0023906::MIMAT0024080 | bta-miR-320(18)::hsa-miR-320(10.1)::mml-miR-320(18)::mmu-miR-320(17)::rno-miR-320(18) | AAAAGCUGGGUUGAGAGGGCGA |
| 002278 | hsa-miR-145 | bta-miR-145::cfa-miR-145::eca-miR-145::hsa-miR-145-5p::mdo-miR-145::mmu-miR-145a-5p::pma-miR-145-5p::rno-miR-145-5p | MIMAT0000157::MIMAT0000437::MIMAT0000851::MIMAT0003542::MIMAT0004116::MIMAT0009863::MIMAT0013064::MIMAT0019476 | hsa-miR-145(17)::mmu-miR-145(17)::mmu-miR-145-5p(18)::pma-miR-145(18)::rno-miR-145(18) | GUCCAGUUUUCCCAGGAAUCCCU |
| 002279 | hsa-miR-31 | bta-miR-31::eca-miR-31::hsa-miR-31-5p | MIMAT0000089::MIMAT0003548::MIMAT0013115 | hsa-miR-31(17) | AGGCAAGAUGCUGGCAUAGCU |
| 002281 | hsa-miR-193a-5p | bta-miR-193a-5p::cfa-miR-193a::eca-miR-193a-5p::hsa-miR-193a-5p::mml-miR-193a-5p::ppy-miR-193a-5p::ssc-miR-193a-5p::tgu-miR-193b-5p | MIMAT0003794::MIMAT0004614::MIMAT0006225::MIMAT0006735::MIMAT0013025::MIMAT0013894::MIMAT0014654::MIMAT0015787 | bta-miR-193a*(13)::tgu-miR-193b*(18) | UGGGUCUUUGCGGGCGAGAUGA |
| 002282 | hsa-let-7g | aca-let-7g::bta-let-7g::cfa-let-7g::cgr-let-7g-5p::eca-let-7g::hsa-let-7g-5p::mml-let-7g::mmu-let-7g-5p::oan-let-7g-5p::ppy-let-7g::ptr-let-7g::ssc-let-7g::tgu-let-7g | MIMAT0000121::MIMAT0000414::MIMAT0003838::MIMAT0006157::MIMAT0006637::MIMAT0007038::MIMAT0007942::MIMAT0013075::MIMAT0013867::MIMAT0014528::MIMAT0015727::MIMAT0021706::MIMAT0023722 | hsa-let-7g(17)::mmu-let-7g(17)::oan-let-7g(18) | UGAGGUAGUAGUUUGUACAGUU |
| 002283 | hsa-let-7d | bta-let-7d::cgr-let-7d-5p::eca-let-7d::hsa-let-7d-5p::mml-let-7d::mmu-let-7d-5p::oan-let-7d-5p::ppy-let-7d::ptr-let-7d::rno-let-7d-5p::ssc-let-7d-5p::tgu-let-7d | MIMAT0000065::MIMAT0000383::MIMAT0000562::MIMAT0003810::MIMAT0006154::MIMAT0007234::MIMAT0007939::MIMAT0013110::MIMAT0014536::MIMAT0015724::MIMAT0023719::MIMAT0025356 | hsa-let-7d(17)::mmu-let-7d(17)::oan-let-7d(18)::rno-let-7d(18) | AGAGGUAGUAGGUUGCAUAGUU |
| 002284 | hsa-miR-138 | bta-miR-138::cfa-miR-138a::eca-miR-138::ggo-miR-138::hsa-miR-138-5p::mml-miR-138::mmu-miR-138-5p::oan-miR-138-5p::pma-miR-138a::ppy-miR-138::ptr-miR-138::rno-miR-138-5p::sha-miR-138::tgu-miR-138 | MIMAT0000150::MIMAT0000430::MIMAT0000844::MIMAT0003813::MIMAT0006194::MIMAT0006654::MIMAT0007216::MIMAT0008034::MIMAT0012925::MIMAT0014515::MIMAT0015760::MIMAT0019469::MIMAT0022771::MIMAT0024098 | hsa-miR-138(17)::mmu-miR-138(17)::oan-miR-138(18)::rno-miR-138(18) | AGCUGGUGUUGUGAAUCAGGCCG |
| 002285 | hsa-miR-186 | bta-miR-186::cfa-miR-186::eca-miR-186::hsa-miR-186-5p::mml-miR-186::mmu-miR-186-5p::oan-miR-186-5p::ppy-miR-186::rno-miR-186-5p | MIMAT0000215::MIMAT0000456::MIMAT0000863::MIMAT0003818::MIMAT0006220::MIMAT0006694::MIMAT0006931::MIMAT0012956::MIMAT0015782 | hsa-miR-186(17)::mmu-miR-186(17)::oan-miR-186(18)::rno-miR-186(18) | CAAAGAAUUCUCCUUUUGGGCU |
| 002286 | hsa-miR-200c# | hsa-miR-200c-5p::mmu-miR-200c-5p | MIMAT0004657::MIMAT0004663 | hsa-miR-200c*(17)::mmu-miR-200c*(17) | CGUCUUACCCAGCAGUGUUUGG |
| 002287 | hsa-miR-155# | hsa-miR-155-3p | MIMAT0004658 | hsa-miR-155*(17) | CUCCUACAUAUUAGCAUUAACA |
| 002288 | hsa-miR-10a# | hsa-miR-10a-3p::mmu-miR-10a-3p::rno-miR-10a-3p | MIMAT0004555::MIMAT0004659::MIMAT0004709 | hsa-miR-10a*(17)::mmu-miR-10a*(17) | CAAAUUCGUAUCUAGGGGAAUA |
| 002289 | hsa-miR-139-5p | cgr-miR-139-5p::eca-miR-139-5p::ggo-miR-139::mml-miR-139-5p::mmu-miR-139-5p::ppy-miR-139-5p::rno-miR-139-5p::ssc-miR-139-5p | MIMAT0000656::MIMAT0000845::MIMAT0002159::MIMAT0006195::MIMAT0012983::MIMAT0015761::MIMAT0023765::MIMAT0024106 | hsa-miR-139-5p(18) | UCUACAGUGCACGUGUCUCCAG |
| 002290 | hsa-miR-208b | bta-miR-208b::cfa-miR-208b::eca-miR-208b::hsa-miR-208b::mml-miR-208b::mmu-miR-208b-3p::ppy-miR-208b::ptr-miR-208b::ssc-miR-208b | MIMAT0004939::MIMAT0004960::MIMAT0006239::MIMAT0008068::MIMAT0009262::MIMAT0009869::MIMAT0012900::MIMAT0013912::MIMAT0015800 | mmu-miR-208b(17) | AUAAGACGAACAAAAGGUUUGU |
| 002292 | hsa-miR-653 | hsa-miR-653::mml-miR-653::mmu-miR-653-5p::ppy-miR-653::ptr-miR-653 | MIMAT0003328::MIMAT0004943::MIMAT0006494::MIMAT0008303::MIMAT0016086 | mmu-miR-653(17) | GUGUUGAAACAAUCUCUACUG |
| 002293 | hsa-miR-214# | aca-miR-214-5p::cgr-miR-214-5p::hsa-miR-214-5p::mmu-miR-214-5p | MIMAT0004564::MIMAT0004664::MIMAT0021871::MIMAT0023851 | aca-miR-214*(18)::hsa-miR-214*(17)::mmu-miR-214*(17) | UGCCUGUCUACACUUGCUGUGC |
| 002294 | hsa-miR-218-2# | hsa-miR-218-2-3p::mmu-miR-218-2-3p::rno-miR-218a-2-3p | MIMAT0004566::MIMAT0004740::MIMAT0005444 | hsa-miR-218-2*(17)::mmu-miR-218-2*(17)::rno-miR-218*(15)::rno-miR-218-2*(16)::rno-miR-218a-2*(18) | CAUGGUUCUGUCAAGCACCGCG |
| 002295 | hsa-miR-223 | bta-miR-223::eca-miR-223::hsa-miR-223-3p::mmu-miR-223-3p | MIMAT0000280::MIMAT0000665::MIMAT0009270::MIMAT0013205 | hsa-miR-223(17)::mmu-miR-223(17) | UGUCAGUUUGUCAAAUACCCCA |
| 002296 | hsa-miR-885-5p | bta-miR-885::cfa-miR-885::eca-miR-885-5p::hsa-miR-885-5p::mml-miR-885-5p::ppy-miR-885-5p::ssc-miR-885-5p | MIMAT0004947::MIMAT0006522::MIMAT0009382::MIMAT0009933::MIMAT0013081::MIMAT0013902::MIMAT0016118 | | UCCAUUACACUACCCUGCCUCU |
| 002297 | hsa-miR-422a | hsa-miR-422a::ptr-miR-422a | MIMAT0001339::MIMAT0008141 | | ACUGGACUUAGGGUCAGAAGGC |
| 002298 | hsa-miR-129# | eca-miR-129a-3p::hsa-miR-129-1-3p::mml-miR-129-3p::mmu-miR-129-1-3p::ppy-miR-129-1-3p | MIMAT0004548::MIMAT0006186::MIMAT0012933::MIMAT0015752::MIMAT0016994 | hsa-miR-129*(17)::ppy-miR-129-1*(18) | AAGCCCUUACCCCAAAAAGUAU |
| 002299 | hsa-miR-191 | aca-miR-191-5p::bta-miR-191::cgr-miR-191-5p::eca-miR-191::hsa-miR-191-5p::mml-miR-191::mmu-miR-191-5p::ppy-miR-191::ptr-miR-191::rno-miR-191a-5p::ssc-miR-191 | MIMAT0000221::MIMAT0000440::MIMAT0000866::MIMAT0003819::MIMAT0006223::MIMAT0008056::MIMAT0013079::MIMAT0013876::MIMAT0015785::MIMAT0021824::MIMAT0023822 | aca-miR-191(18)::hsa-miR-191(17)::mmu-miR-191(17)::rno-miR-191(18) | CAACGGAAUCCCAAAAGCAGCUG |
| 002300 | hsa-miR-200c | bta-miR-200c::cfa-miR-200c::cgr-miR-200c::eca-miR-200c::hsa-miR-200c-3p::mmu-miR-200c-3p::ptr-miR-200c | MIMAT0000617::MIMAT0000657::MIMAT0003823::MIMAT0006664::MIMAT0012974::MIMAT0023843::MIMAT0024051 | hsa-miR-200c(17)::mmu-miR-200c(17) | UAAUACUGCCGGGUAAUGAUGGA |
| 002301 | hsa-miR-22# | bta-miR-22-5p::gga-miR-22-5p::hsa-miR-22-5p::mmu-miR-22-5p::oan-miR-22-5p::rno-miR-22-5p::ssc-miR-22-5p | MIMAT0003152::MIMAT0003826::MIMAT0004495::MIMAT0004629::MIMAT0006961::MIMAT0007287::MIMAT0015709 | gga-miR-22*(18)::hsa-miR-22*(17)::mmu-miR-22*(17)::oan-miR-22*(18)::rno-miR-22*(18) | AGUUCUUCAGUGGCAAGCUUUA |
| 002302 | hsa-miR-425# | bta-miR-425-3p::hsa-miR-425-3p::ssc-miR-425-3p | MIMAT0001343::MIMAT0003833::MIMAT0013918 | hsa-miR-425*(17) | AUCGGGAAUGUCGUGUCCGCCC |
| 002303 | hsa-miR-450a | bta-miR-450a::cgr-miR-450a::eca-miR-450a::hsa-miR-450a-5p::mml-miR-450a::mmu-miR-450a-5p::ptr-miR-450a::ssc-miR-450a | MIMAT0001545::MIMAT0001546::MIMAT0003834::MIMAT0006332::MIMAT0008151::MIMAT0010188::MIMAT0013219::MIMAT0023961 | bta-miR-450(18)::hsa-miR-450a(17)::mmu-miR-450a(17)::ssc-miR-450(14) | UUUUGCGAUGUGUUCCUAAUAU |
| 002304 | hsa-miR-199a-3p | bta-miR-199a-3p::eca-miR-199a-3p::eca-miR-199b-3p::hsa-miR-199a-3p::hsa-miR-199b-3p::mml-miR-199a-3p::mmu-miR-199a-3p::mmu-miR-199b-3p::ola-miR-199a-3p::pol-miR-199a-3p::ppy-miR-199a-3p::ptr-miR-199a-3p::ptr-miR-199b::rno-miR-199a-3p::ssc-miR-199a-3p | MIMAT0000230::MIMAT0000232::MIMAT0003746::MIMAT0004563::MIMAT0004667::MIMAT0004738::MIMAT0006232::MIMAT0008062::MIMAT0009192::MIMAT0012961::MIMAT0013781::MIMAT0013875::MIMAT0015793::MIMAT0022539::MIMAT0025441 | bta-miR-199a*(9.1)::mmu-miR-199b(17) | ACAGUAGUCUGCACAUUGGUUA |
| 002305 | hsa-miR-30d# | cgr-miR-30d::hsa-miR-30d-3p::mmu-miR-30d-3p::rno-miR-30d-3p | MIMAT0004551::MIMAT0004722::MIMAT0017011::MIMAT0023899 | hsa-miR-30d*(17)::mmu-miR-30d*(17)::rno-miR-30d*(18) | CUUUCAGUCAGAUGUUUGCUGC |
| 002306 | hsa-miR-214 | aca-miR-214-3p::bta-miR-214::cfa-miR-214::eca-miR-214::hsa-miR-214-3p::mmu-miR-214-3p::oan-miR-214-3p::tgu-miR-214 | MIMAT0000271::MIMAT0000661::MIMAT0003825::MIMAT0007171::MIMAT0009847::MIMAT0012963::MIMAT0014603::MIMAT0021872 | aca-miR-214(18)::hsa-miR-214(17)::mmu-miR-214(17)::oan-miR-214(18) | ACAGCAGGCACAGACAGGCAGU |
| 002307 | hsa-let-7a# | bta-let-7a-3p::hsa-let-7a-3p | MIMAT0004330::MIMAT0004481 | bta-let-7a*(18)::hsa-let-7a*(17) | CUAUACAAUCUACUGUCUUUC |
| 002308 | hsa-miR-17 | aca-miR-17-5p::ccr-miR-17-5p::cgr-miR-17-5p::eca-miR-106a::eca-miR-17::hsa-miR-17-5p::mmu-miR-17-5p::oan-miR-17-5p::rno-miR-17-5p::ssc-miR-17-5p::tgu-miR-17a-5p | MIMAT0000070::MIMAT0000649::MIMAT0000786::MIMAT0007278::MIMAT0007755::MIMAT0013084::MIMAT0013194::MIMAT0017390::MIMAT0021803::MIMAT0023795::MIMAT0026236 | aca-miR-17(18)::hsa-miR-17(17)::mmu-miR-17(17)::oan-miR-17(18)::rno-miR-17(12)::ssc-miR-17(14) | CAAAGUGCUUACAGUGCAGGUAG |
| 002309 | hsa-miR-424# | bta-miR-424-3p::cfa-miR-424::hsa-miR-424-3p::ssc-miR-424-3p | MIMAT0004749::MIMAT0006745::MIMAT0013921::MIMAT0015304 | bta-miR-424*(18)::hsa-miR-424*(17)::ssc-miR-424*(18) | CAAAACGUGAGGCGCUGCUAU |
| 002310 | hsa-miR-18b# | hsa-miR-18b-3p | MIMAT0004751 | hsa-miR-18b*(17) | UGCCCUAAAUGCCCCUUCUGGC |
| 002311 | hsa-miR-20b# | hsa-miR-20b-3p | MIMAT0004752 | hsa-miR-20b*(17) | ACUGUAGUAUGGGCACUUCCAG |
| 002312 | hsa-miR-431# | hsa-miR-431-3p::mmu-miR-431-3p | MIMAT0004753::MIMAT0004757 | hsa-miR-431*(17)::mmu-miR-431*(17) | CAGGUCGUCUUGCAGGGCUUCU |
| 002313 | hsa-miR-139-3p | eca-miR-139-3p::mml-miR-139-3p::ppy-miR-139-3p::ptr-miR-139 | MIMAT0006196::MIMAT0008035::MIMAT0012984::MIMAT0015762 | hsa-miR-139-3p(18) | GGAGACGCGGCCCUGUUGGAGU |
| 002314 | hsa-miR-7-2# | hsa-miR-7-2-3p | MIMAT0004554 | hsa-miR-7-2*(17) | CAACAAAUCCCAGUCUACCUAA |
| 002315 | hsa-miR-10b# | cgr-miR-10b-3p::hsa-miR-10b-3p | MIMAT0004556::MIMAT0023738 | hsa-miR-10b*(17) | ACAGAUUCGAUUCUAGGGGAAU |
| 002316 | hsa-miR-34a# | hsa-miR-34a-3p | MIMAT0004557 | hsa-miR-34a*(17) | CAAUCAGCAAGUAUACUGCCCU |
| 002317 | hsa-miR-181a-2# | hsa-miR-181a-2-3p | MIMAT0004558 | hsa-miR-181a-2*(17) | ACCACUGACCGUUGACUGUACC |
| 002318 | hsa-miR-453 | bta-miR-453::hsa-miR-323b-5p::mml-miR-453::ppy-miR-453::ptr-miR-453 | MIMAT0001630::MIMAT0006337::MIMAT0008155::MIMAT0009325::MIMAT0015898 | hsa-miR-453(14) | AGGUUGUCCGUGGUGAGUUCGCA |
| 002322 | hsa-miR-671-3p | cfa-miR-671::cgr-miR-671-3p::eca-miR-671-3p::ggo-miR-671::hsa-miR-671-3p::mml-miR-671-3p::mmu-miR-671-3p::ppy-miR-671-3p::ptr-miR-671::rno-miR-671::ssc-miR-671-3p | MIMAT0004819::MIMAT0004821::MIMAT0005326::MIMAT0006506::MIMAT0008314::MIMAT0009926::MIMAT0012949::MIMAT0016097::MIMAT0023997::MIMAT0024279::MIMAT0025382 | | UCCGGUUCUCAGGGCUCCACC |
| 002323 | hsa-miR-454 | aca-miR-454-3p::bta-miR-454::eca-miR-454::gga-miR-454-3p::hsa-miR-454-3p::mml-miR-454::oan-miR-454-3p::ptr-miR-454::sha-miR-454::tgu-miR-454 | MIMAT0003885::MIMAT0006338::MIMAT0007157::MIMAT0007292::MIMAT0008156::MIMAT0009326::MIMAT0013042::MIMAT0014580::MIMAT0021963::MIMAT0022815 | aca-miR-454(18)::gga-miR-454(18)::hsa-miR-454(17)::oan-miR-454(18) | UAGUGCAAUAUUGCUUAUAGGGU |
| 002324 | hsa-miR-744 | bta-miR-744::hsa-miR-744-5p::mmu-miR-744-5p::ppy-miR-744::ptr-miR-744::ssc-miR-744 | MIMAT0004187::MIMAT0004945::MIMAT0008317::MIMAT0009369::MIMAT0015708::MIMAT0016102 | hsa-miR-744(17)::mmu-miR-744(17) | UGCGGGGCUAGGGCUAACAGCA |
| 002325 | hsa-miR-744# | hsa-miR-744-3p::mmu-miR-744-3p | MIMAT0004820::MIMAT0004946 | hsa-miR-744*(17)::mmu-miR-744*(17) | CUGUUGCCACUAACCUCAACCU |
| 002326 | hsa-miR-216b | aca-miR-216b-5p::bta-miR-216b::cfa-miR-216b::eca-miR-216b::gga-miR-216b::hsa-miR-216b::mml-miR-216b::mmu-miR-216b-5p::ppy-miR-216b::ptr-miR-216b::rno-miR-216b-5p | MIMAT0003729::MIMAT0004959::MIMAT0006243::MIMAT0006592::MIMAT0007748::MIMAT0008072::MIMAT0009266::MIMAT0013073::MIMAT0015802::MIMAT0017846::MIMAT0021876 | aca-miR-216b(18)::cfa-miR-216(12)::mmu-miR-216b(17) | AAAUCUCUGCAGGCAAAUGUGA |
| 002327 | hsa-miR-672 | cgr-miR-672::eca-miR-672::mmu-miR-672-5p::rno-miR-672-5p | MIMAT0003735::MIMAT0005327::MIMAT0013242::MIMAT0023998 | hsa-miR-672(10)::mmu-miR-672(17)::rno-miR-672(18) | UGAGGUUGGUGUACUGUGUGUGA |
| 002329 | hsa-miR-452 | cfa-miR-452::ggo-miR-452::hsa-miR-452-5p::mml-miR-452::ppy-miR-452::ptr-miR-452::ssc-miR-452 | MIMAT0001635::MIMAT0006336::MIMAT0008154::MIMAT0009900::MIMAT0015897::MIMAT0024211::MIMAT0025374 | hsa-miR-452(17) | AACUGUUUGCAGAGGAAACUGA |
| 002330 | hsa-miR-452# | hsa-miR-452-3p | MIMAT0001636 | hsa-miR-452*(17) | CUCAUCUGCAAAGAAGUAAGUG |
| 002331 | hsa-miR-409-5p | bta-miR-409a::eca-miR-409-5p::hsa-miR-409-5p::mml-miR-409-5p::mmu-miR-409-5p::oar-miR-409-5p::ppy-miR-409-5p::rno-miR-409a-5p | MIMAT0001638::MIMAT0003204::MIMAT0004746::MIMAT0006314::MIMAT0009310::MIMAT0013151::MIMAT0015875::MIMAT0019327 | bta-miR-409(16)::rno-miR-409-5p(18) | AGGUUACCCGAGCAACUUUGCAU |
| 002332 | hsa-miR-409-3p | cgr-miR-409-3p::eca-miR-409-3p::hsa-miR-409-3p::mml-miR-409-3p::mmu-miR-409-3p::ppy-miR-409-3p::ptr-miR-409 | MIMAT0001090::MIMAT0001639::MIMAT0006315::MIMAT0008136::MIMAT0013152::MIMAT0015876::MIMAT0023951 | | GAAUGUUGCUCGGUGAACCCCU |
| 002333 | hsa-miR-181c# | hsa-miR-181c-3p | MIMAT0004559 | hsa-miR-181c*(17) | AACCAUCGACCGUUGAGUGGAC |
| 002334 | hsa-miR-182 | bta-miR-182::cfa-miR-182::hsa-miR-182-5p::pma-miR-182::ptr-miR-182::ssc-miR-182 | MIMAT0000259::MIMAT0008050::MIMAT0009244::MIMAT0009841::MIMAT0019490::MIMAT0025366 | hsa-miR-182(17) | UUUGGCAAUGGUAGAACUCACACU |
| 002336 | hsa-miR-196a# | hsa-miR-196a-3p | MIMAT0004562 | hsa-miR-196a*(17) | CGGCAACAAGAAACUGCCUGAG |
| 002337 | hsa-miR-217 | bfl-miR-217::ccr-miR-217::eca-miR-217::hsa-miR-217::mml-miR-217b::oan-miR-217-5p::pma-miR-217a::ppy-miR-217::ptr-miR-217 | MIMAT0000274::MIMAT0007221::MIMAT0008073::MIMAT0009496::MIMAT0013074::MIMAT0015803::MIMAT0019528::MIMAT0024354::MIMAT0026270 | oan-miR-217(18) | UACUGCAUCAGGAACUGAUUGGA |
| 002338 | hsa-miR-483-5p | ggo-miR-483::hsa-miR-483-5p | MIMAT0004761::MIMAT0024191 | | AAGACGGGAGGAAAGAAGGGAG |
| 002339 | hsa-miR-483-3p | bta-miR-483::hsa-miR-483-3p | MIMAT0002173::MIMAT0009327 | | UCACUCCUCUCCUCCCGUCUU |
| 002340 | hsa-miR-423-5p | bta-miR-423-5p::cfa-miR-423a::cgr-miR-423-5p::eca-miR-423-5p::hsa-miR-423-5p::mml-miR-423-5p::mmu-miR-423-5p::ppy-miR-423-5p::ssc-miR-423-5p | MIMAT0004748::MIMAT0004825::MIMAT0006321::MIMAT0006742::MIMAT0012537::MIMAT0013039::MIMAT0013880::MIMAT0015882::MIMAT0023956 | | UGAGGGGCAGAGAGCGAGACUUU |
| 002341 | hsa-miR-708 | bta-miR-708::cfa-miR-708::eca-miR-708::hsa-miR-708-5p::mml-miR-708::mmu-miR-708-5p::ppy-miR-708::ptr-miR-708::rno-miR-708-5p::ssc-miR-708-5p | MIMAT0004828::MIMAT0004926::MIMAT0005331::MIMAT0006643::MIMAT0008315::MIMAT0009367::MIMAT0012785::MIMAT0012993::MIMAT0013945::MIMAT0016100 | hsa-miR-708(17)::mmu-miR-708(17)::rno-miR-708(18)::ssc-miR-708(15) | AAGGAGCUUACAAUCUAGCUGGG |
| 002342 | hsa-miR-708# | hsa-miR-708-3p::mmu-miR-708-3p::rno-miR-708-3p | MIMAT0003498::MIMAT0004927::MIMAT0005332 | hsa-miR-708*(17)::mmu-miR-708*(17)::rno-miR-708*(18) | CAACUAGACUGUGAGCUUCUAG |
| 002343 | hsa-miR-92b# | hsa-miR-92b-5p | MIMAT0004792 | hsa-miR-92b*(17) | AGGGACGGGACGCGGUGCAGUG |
| 002344 | hsa-miR-556-5p | hsa-miR-556-5p::ppy-miR-556-5p | MIMAT0003220::MIMAT0016002 | | GAUGAGCUCAUUGUAAUAUGAG |
| 002345 | hsa-miR-556-3p | hsa-miR-556-3p::ptr-miR-556 | MIMAT0004793::MIMAT0008237 | | AUAUUACCAUUAGCUCAUCUUU |
| 002346 | hsa-miR-551b# | hsa-miR-551b-5p | MIMAT0004794 | hsa-miR-551b*(17) | GAAAUCAAGCGUGGGUGAGACC |
| 002347 | hsa-miR-570 | hsa-miR-570-3p | MIMAT0003235 | hsa-miR-570(17) | CGAAAACAGCAAUUACCUUUGC |
| 002349 | hsa-miR-574-3p | cfa-miR-574::ggo-miR-574::hsa-miR-574-3p::mmu-miR-574-3p::ssc-miR-574 | MIMAT0003239::MIMAT0004894::MIMAT0006673::MIMAT0013951::MIMAT0024162 | | CACGCUCAUGCACACACCCACA |
| 002350 | hsa-miR-576-5p | hsa-miR-576-5p | MIMAT0003241 |  | AUUCUAAUUUCUCCACGUCUUU |
| 002351 | hsa-miR-576-3p | hsa-miR-576-3p::mml-miR-576-3p::ppy-miR-576-3p::ptr-miR-576 | MIMAT0004796::MIMAT0006437::MIMAT0008250::MIMAT0016018 | | AAGAUGUGGAAAAAUUGGAAUC |
| 002352 | hsa-miR-652 | bta-miR-652::cgr-miR-652-3p::eca-miR-652::ggo-miR-652::hsa-miR-652-3p::mml-miR-652::mmu-miR-652-3p::ppy-miR-652::ptr-miR-652::rno-miR-652-3p | MIMAT0003322::MIMAT0003711::MIMAT0005342::MIMAT0006493::MIMAT0008302::MIMAT0013240::MIMAT0016085::MIMAT0023992::MIMAT0024153::MIMAT0024578 | hsa-miR-652(17)::mmu-miR-652(17)::rno-miR-652(18) | AAUGGCGCCACUAGGGUUGUG |
| 002353 | hsa-miR-615-5p | bta-miR-615::cgr-miR-615-5p::eca-miR-615-5p::hsa-miR-615-5p::mml-miR-615-5p::mmu-miR-615-5p::rno-miR-615 | MIMAT0004804::MIMAT0004837::MIMAT0006465::MIMAT0009355::MIMAT0012835::MIMAT0012976::MIMAT0023987 | | GGGGGUCCCCGGUGCUCGGAUC |
| 002354 | hsa-miR-871 | mmu-miR-871-5p | MIMAT0004841 | hsa-miR-871(10)::mmu-miR-871(15) | UAUUCAGAUUAGUGCCAGUCAUG |
| 002355 | hsa-miR-532-3p | eca-miR-532-3p::hsa-miR-532-3p::mml-miR-532-3p::mmu-miR-532-3p::ppy-miR-532-3p::ptr-miR-532::rno-miR-532-3p::ssc-miR-532-3p | MIMAT0004780::MIMAT0004781::MIMAT0005323::MIMAT0006404::MIMAT0008213::MIMAT0013236::MIMAT0013941::MIMAT0015988 | | CCUCCCACACCCAAGGCUUGCA |
| 002356 | hsa-miR-873 | bta-miR-873::eca-miR-873::hsa-miR-873-5p::mmu-miR-873a-5p::ppy-miR-873::ptr-miR-873::rno-miR-873-5p | MIMAT0004936::MIMAT0004953::MIMAT0005339::MIMAT0008324::MIMAT0009377::MIMAT0013120::MIMAT0016111 | hsa-miR-873(17)::mmu-miR-873(17)::mmu-miR-873-5p(18)::rno-miR-873(18) | GCAGGAACUUGUGAGUCUCCU |
| 002357 | hsa-miR-488 | eca-miR-488::hsa-miR-488-3p::mml-miR-488::ppy-miR-488::ptr-miR-488 | MIMAT0004763::MIMAT0006348::MIMAT0008163::MIMAT0012965::MIMAT0015907 | hsa-miR-488(17) | UUGAAAGGCUAUUUCUUGGUC |
| 002358 | hsa-miR-489 | hsa-miR-489::mml-miR-489::ppy-miR-489::ptr-miR-489 | MIMAT0002805::MIMAT0006349::MIMAT0008164::MIMAT0015908 | | GUGACAUCACAUAUACGGCAGC |
| 002360 | hsa-miR-491-3p | eca-miR-491-3p::hsa-miR-491-3p::mml-miR-491-3p::mmu-miR-491-3p::ppy-miR-491-3p::ptr-miR-491 | MIMAT0004765::MIMAT0006353::MIMAT0008166::MIMAT0013117::MIMAT0015912::MIMAT0017255 | mmu-miR-491*(17) | CUUAUGCAAGAUUCCCUUCUAC |
| 002361 | hsa-miR-146b-3p | hsa-miR-146b-3p::mml-miR-146b-3p::ppy-miR-146b-3p | MIMAT0004766::MIMAT0006205::MIMAT0015769 | | UGCCCUGUGGACUCAGUUCUGG |
| 002362 | hsa-miR-202# | cfa-miR-202::hsa-miR-202-5p | MIMAT0002810::MIMAT0009844 | hsa-miR-202*(17) | UUCCUAUGCAUAUACUUCUUUG |
| 002363 | hsa-miR-202 | hsa-miR-202-3p::ptr-miR-202::tgu-miR-202 | MIMAT0002811::MIMAT0008064::MIMAT0014565 | hsa-miR-202(17) | AGAGGUAUAGGGCAUGGGAA |
| 002364 | hsa-miR-493 | bta-miR-493::hsa-miR-493-3p::mml-miR-493::oar-miR-493-3p::ppy-miR-493::ssc-miR-493-3p | MIMAT0003161::MIMAT0006355::MIMAT0009333::MIMAT0015914::MIMAT0019238::MIMAT0025378 | hsa-miR-493(17) | UGAAGGUCUACUGUGUGCCAGG |
| 002365 | hsa-miR-494 | bta-miR-494::cfa-miR-494::eca-miR-494::ggo-miR-494::hsa-miR-494::mml-miR-494::mmu-miR-494-3p::ppy-miR-494::ptr-miR-494 | MIMAT0002816::MIMAT0003182::MIMAT0006356::MIMAT0008168::MIMAT0009334::MIMAT0009905::MIMAT0013164::MIMAT0015915::MIMAT0024160 | mmu-miR-494(17) | UGAAACAUACACGGGAAACCUC |
| 002366 | hsa-miR-193b# | cfa-miR-193b::cgr-miR-193b-5p::ggo-miR-193b::hsa-miR-193b-5p::mmu-miR-193b-5p::oan-miR-193-5p | MIMAT0004767::MIMAT0006699::MIMAT0006820::MIMAT0017271::MIMAT0023825::MIMAT0024138 | hsa-miR-193b*(17)::mmu-miR-193b*(17)::oan-miR-193*(18) | CGGGGUUUUGAGGGCGAGAUGA |
| 002367 | hsa-miR-193b | hsa-miR-193b-3p::mml-miR-193b::ppy-miR-193b::ptr-miR-193b | MIMAT0002819::MIMAT0006227::MIMAT0008059::MIMAT0015789 | hsa-miR-193b(17) | AACUGGCCCUCAAAGUCCCGCU |
| 002368 | hsa-miR-497# | cgr-miR-497-3p::hsa-miR-497-3p | MIMAT0004768::MIMAT0023971 | hsa-miR-497*(17) | CAAACCACACUGUGGUGUUAGA |
| 002369 | hsa-miR-515-3p | hsa-miR-515-3p::ppy-miR-515-3p::ptr-miR-515 | MIMAT0002827::MIMAT0008181::MIMAT0015944 | | GAGUGCCUUCUUUUGGAGCGUU |
| 002370 | hsa-miR-519e | hsa-miR-519e-3p::ppy-miR-519e::ptr-miR-519e | MIMAT0002829::MIMAT0008196::MIMAT0015965 | hsa-miR-519e(17) | AAGUGCCUCCUUUUAGAGUGUU |
| 002371 | hsa-miR-518e# | hsa-miR-518e-5p::hsa-miR-519a-5p::hsa-miR-519b-5p::hsa-miR-519c-5p::hsa-miR-522-5p::hsa-miR-523-5p::ppy-miR-519b-5p::ppy-miR-519c-5p | MIMAT0002831::MIMAT0005449::MIMAT0005450::MIMAT0005451::MIMAT0005452::MIMAT0005454::MIMAT0015961::MIMAT0015963 | hsa-miR-518e*(17)::hsa-miR-519a*(17)::hsa-miR-522*(17)::hsa-miR-523*(17) | CUCUAGAGGGAAGCGCUUUCUG |
| 002372 | hsa-miR-885-3p | eca-miR-885-3p::hsa-miR-885-3p::mml-miR-885-3p::ppy-miR-885-3p::ptr-miR-885 | MIMAT0004948::MIMAT0006523::MIMAT0008328::MIMAT0013082::MIMAT0016119 | | AGGCAGCGGGGUGUAGUGGAUA |
| 002374 | hsa-miR-887 | hsa-miR-887::mml-miR-887::ppy-miR-887::ptr-miR-887 | MIMAT0004951::MIMAT0006526::MIMAT0008330::MIMAT0016122 | | GUGAACGGGCGCCAUCCCGAGG |
| 002376 | hsa-miR-543 | bta-miR-543::cfa-miR-543::eca-miR-543::hsa-miR-543::mml-miR-543::mmu-miR-543-3p::ppy-miR-543::ptr-miR-543 | MIMAT0003168::MIMAT0004954::MIMAT0006720::MIMAT0008215::MIMAT0009344::MIMAT0012787::MIMAT0013169::MIMAT0015993 | mmu-miR-543(17) | AAACAUUCGCGGUGCACUUCUU |
| 002378 | hsa-miR-125b-1# | hsa-miR-125b-1-3p::mmu-miR-125b-1-3p::rno-miR-125b-1-3p | MIMAT0004592::MIMAT0004669::MIMAT0004730 | hsa-miR-125b-1*(17)::mmu-miR-125b-3p(15)::rno-miR-125b-3p(18) | ACGGGUUAGGCUCUUGGGAGCU |
| 002379 | hsa-miR-194# | hsa-miR-194-3p::mmu-miR-194-2-3p | MIMAT0004671::MIMAT0017073 | hsa-miR-194*(17)::mmu-miR-194-2*(17) | CCAGUGGGGCUGCUGUUAUCUG |
| 002380 | hsa-miR-106b# | cgr-miR-106b-3p::hsa-miR-106b-3p::mmu-miR-106b-3p::rno-miR-106b-3p | MIMAT0004582::MIMAT0004672::MIMAT0004727::MIMAT0023733 | hsa-miR-106b*(17)::mmu-miR-106b*(17)::rno-miR-106b*(18) | CCGCACUGUGGGUACUUGCUGC |
| 002381 | hsa-miR-302a# | cfa-miR-302a::hsa-miR-302a-5p | MIMAT0000683::MIMAT0009855 | hsa-miR-302a*(17) | ACUUAAACGUGGAUGUACUUGCU |
| 002382 | hsa-miR-526b | hsa-miR-526b-5p::mml-miR-526b::ppy-miR-526b::ptr-miR-526b | MIMAT0002835::MIMAT0008211::MIMAT0012776::MIMAT0015985 | hsa-miR-526b(17) | CUCUUGAGGGAAGCACUUUCUGU |
| 002384 | hsa-miR-519b-3p | hsa-miR-519b-3p::ppy-miR-519a::ppy-miR-519b-3p::ptr-miR-519b | MIMAT0002837::MIMAT0008193::MIMAT0015959::MIMAT0015962 | | AAAGUGCAUCCUUUUAGAGGUU |
| 002385 | hsa-miR-525-3p | hsa-miR-525-3p::ptr-miR-525 | MIMAT0002839::MIMAT0008209 | | GAAGGCGCUUCCCUUUAGAGCG |
| 002386 | hsa-miR-523 | hsa-miR-523-3p::ptr-miR-523 | MIMAT0002840::MIMAT0008207 | hsa-miR-523(17) | GAACGCGCUUCCCUAUAGAGGGU |
| 002387 | hsa-miR-518f# | hsa-miR-518f-5p | MIMAT0002841 | hsa-miR-518f*(17) | CUCUAGAGGGAAGCACUUUCUC |
| 002388 | hsa-miR-518f | hsa-miR-518f-3p::ppy-miR-518f::ptr-miR-518f | MIMAT0002842::MIMAT0008191::MIMAT0015958 | hsa-miR-518f(17) | GAAAGCGCUUCUCUUUAGAGG |
| 002389 | hsa-miR-518d-5p | hsa-miR-518d-5p::hsa-miR-520c-5p::hsa-miR-526a::ptr-miR-526a | MIMAT0002845::MIMAT0005455::MIMAT0005456::MIMAT0008210 | | CUCUAGAGGGAAGCACUUUCUG |
| 002390 | hsa-miR-219-2-3p | cfa-miR-219-3p::hsa-miR-219-2-3p::mml-miR-219-3p::mmu-miR-219-2-3p::ptr-miR-219-2-3p::rno-miR-219-2-3p | MIMAT0004675::MIMAT0005446::MIMAT0006246::MIMAT0006729::MIMAT0009194::MIMAT0022841 | cfa-miR-219*(18)::mmu-miR-219-3p(17) | AGAAUUGUGGCUGGACAUCUGU |
| 002391 | hsa-miR-374b# | hsa-miR-374b-3p | MIMAT0004956 | hsa-miR-374b*(17) | CUUAGCAGGUUGUAUUAUCAUU |
| 002392 | hsa-miR-301b | cfa-miR-301b::eca-miR-301b-3p::hsa-miR-301b::mml-miR-301b::ppy-miR-301b::ptr-miR-301b | MIMAT0004958::MIMAT0006258::MIMAT0008085::MIMAT0009854::MIMAT0013001::MIMAT0015816 | | CAGUGCAAUGAUAUUGUCAAAGC |
| 002393 | hsa-miR-520d-5p | hsa-miR-520d-5p::mml-miR-518a-5p::ppy-miR-520d-5p | MIMAT0002855::MIMAT0006377::MIMAT0015971 | mml-miR-520d-5p(15) | CUACAAAGGGAAGCCCUUUC |
| 002395 | hsa-miR-518e | hsa-miR-518e-3p::ptr-miR-518e | MIMAT0002861::MIMAT0008190 | hsa-miR-518e(17) | AAAGCGCUUCCCUUCAGAGUG |
| 002396 | hsa-miR-518a-5p | hsa-miR-518a-5p::hsa-miR-527::ppy-miR-518a-5p::ppy-miR-527::ptr-miR-527 | MIMAT0002862::MIMAT0005457::MIMAT0008212::MIMAT0015951::MIMAT0015986 | | CUGCAAAGGGAAGCCCUUUC |
| 002397 | hsa-miR-518a-3p | hsa-miR-518a-3p::ptr-miR-518a | MIMAT0002863::MIMAT0008186 | | GAAAGCGCUUCCCUUUGCUGGA |
| 002398 | hsa-miR-579 | hsa-miR-579::ppy-miR-579::ptr-miR-579 | MIMAT0003244::MIMAT0008252::MIMAT0016021 | | UUCAUUUGGUAUAAACCGCGAUU |
| 002399 | hsa-miR-582-3p | hsa-miR-582-3p::mml-miR-582-3p::ppy-miR-582-3p | MIMAT0004797::MIMAT0006444::MIMAT0016025 | | UAACUGGUUGAACAACUGAACC |
| 002400 | hsa-miR-520c-3p | hsa-miR-520c-3p::ppy-miR-520c-3p | MIMAT0002846::MIMAT0015970 | | AAAGUGCUUCCUUUUAGAGGGU |
| 002401 | hsa-miR-518c | hsa-miR-518c-3p::ppy-miR-518c::ptr-miR-518c | MIMAT0002848::MIMAT0008188::MIMAT0015954 | hsa-miR-518c(17) | CAAAGCGCUUCUCUUUAGAGUGU |
| 002402 | hsa-miR-517a | hsa-miR-517a-3p::hsa-miR-517b-3p::ppy-miR-517a::ppy-miR-517c::ptr-miR-517a | MIMAT0002852::MIMAT0002857::MIMAT0008184::MIMAT0015948::MIMAT0015950 | hsa-miR-517a(17) | AUCGUGCAUCCCUUUAGAGUGU |
| 002403 | hsa-miR-519d | hsa-miR-519d::ppy-miR-519d::ptr-miR-519d | MIMAT0002853::MIMAT0008195::MIMAT0015964 | | CAAAGUGCCUCCCUUUAGAGUG |
| 002404 | hsa-let-7b# | hsa-let-7b-3p::mmu-let-7b-3p::rno-let-7b-3p | MIMAT0004482::MIMAT0004621::MIMAT0004705 | hsa-let-7b*(17)::mmu-let-7b*(17)::rno-let-7b*(18) | CUAUACAACCUACUGCCUUCCC |
| 002405 | hsa-let-7c# |  |  | hsa-let-7c*(16) | UAGAGUUACACCCUGGGAGUUA |
| 002406 | hsa-let-7e | cfa-let-7e::eca-let-7e::hsa-let-7e-5p::mml-let-7e::mmu-let-7e-5p::ppy-let-7e::ptr-let-7e::rno-let-7e-5p::ssc-let-7e | MIMAT0000066::MIMAT0000524::MIMAT0000777::MIMAT0006155::MIMAT0006608::MIMAT0007940::MIMAT0013007::MIMAT0013866::MIMAT0015725 | hsa-let-7e(17)::mmu-let-7e(17)::rno-let-7e(18) | UGAGGUAGGAGGUUGUAUAGUU |
| 002407 | hsa-let-7e# | hsa-let-7e-3p::mmu-let-7e-3p::rno-let-7e-3p | MIMAT0004485::MIMAT0004706::MIMAT0017016 | hsa-let-7e*(17)::mmu-let-7e*(17)::rno-let-7e*(18) | CUAUACGGCCUCCUAGCUUUCC |
| 002408 | hsa-miR-548b-5p | hsa-miR-548b-5p | MIMAT0004798 |  | AAAAGUAAUUGUGGUUUUGGCC |
| 002409 | hsa-miR-589 | hsa-miR-589-5p::mml-miR-589::ppy-miR-589 | MIMAT0004799::MIMAT0006449::MIMAT0016031 | hsa-miR-589(17) | UGAGAACCACGUCUGCUCUGAG |
| 002410 | hsa-miR-550 | hsa-miR-550a-5p::mml-miR-550::ptr-miR-550 | MIMAT0004800::MIMAT0006418::MIMAT0008230 | hsa-miR-550(15)::hsa-miR-550a(17) | AGUGCCUGAGGGAGUAAGAGCCC |
| 002411 | hsa-miR-593 | hsa-miR-593-3p::mml-miR-593::ppy-miR-593::ptr-miR-593 | MIMAT0004802::MIMAT0006453::MIMAT0008264::MIMAT0016036 | hsa-miR-593(17) | UGUCUCUGCUGGGGUUUCU |
| 002412 | hsa-miR-548a-5p | hsa-miR-548a-5p | MIMAT0004803 |  | AAAAGUAAUUGCGAGUUUUACC |
| 002413 | hsa-miR-522 | hsa-miR-522-3p::mml-miR-518e::ppy-miR-522::ptr-miR-522 | MIMAT0002868::MIMAT0006382::MIMAT0008206::MIMAT0015978 | hsa-miR-522(17)::mml-miR-522(15) | AAAAUGGUUCCCUUUAGAGUGU |
| 002414 | hsa-miR-616 | hsa-miR-616-3p::ptr-miR-616 | MIMAT0004805::MIMAT0008278 | hsa-miR-616(17) | AGUCAUUGGAGGGUUUGAGCAG |
| 002415 | hsa-miR-519a | hsa-miR-519a-3p::ppy-miR-519f::ptr-miR-519a | MIMAT0002869::MIMAT0008192::MIMAT0015960 | hsa-miR-519a(17) | AAAGUGCAUCCUUUUAGAGUGU |
| 002416 | hsa-miR-516a-5p | hsa-miR-516a-5p::ppy-miR-516a-5p | MIMAT0004770::MIMAT0004771::MIMAT0015945 | | UUCUCGAGGAAAGAAGCACUUUC |
| 002417 | hsa-let-7f-1# | hsa-let-7f-1-3p::mmu-let-7f-1-3p | MIMAT0004486::MIMAT0004623 | hsa-let-7f-1*(17)::mmu-let-7f*(15)::mmu-let-7f-1*(17) | CUAUACAAUCUAUUGCCUUCCC |
| 002418 | hsa-let-7f-2# | hsa-let-7f-2-3p | MIMAT0004487 | hsa-let-7f-2*(17) | CUAUACAGUCUACUGUCUUUCC |
| 002419 | hsa-miR-15a# | cgr-miR-15a-3p::hsa-miR-15a-3p::oan-miR-15a-3p | MIMAT0004488::MIMAT0007165::MIMAT0023790 | hsa-miR-15a*(17)::oan-miR-15a*(18) | CAGGCCAUAUUGUGCUGCCUCA |
| 002420 | hsa-miR-16-1# | hsa-miR-16-1-3p::tgu-miR-16a-3p | MIMAT0004489::MIMAT0014647 | hsa-miR-16-1*(17)::tgu-miR-16a*(18) | CCAGUAUUAACUGUGCUGCUGA |
| 002421 | hsa-miR-17# | aca-miR-17-3p::cfa-miR-17::hsa-miR-17-3p::ssc-miR-17-3p::tgu-miR-17a-3p | MIMAT0000071::MIMAT0006649::MIMAT0014564::MIMAT0015268::MIMAT0021804 | aca-miR-17*(18)::hsa-miR-17*(17)::tgu-miR-17a(15) | ACUGCAGUGAAGGCACUUGUAG |
| 002422 | hsa-miR-18a | aca-miR-18a-5p::ccr-miR-18a::cgr-miR-18a-5p::eca-miR-18a::hsa-miR-18a-5p::mmu-miR-18a-5p::ola-miR-18::pma-miR-18a-5p::rno-miR-18a-5p::xtr-miR-18a-5p | MIMAT0000072::MIMAT0000528::MIMAT0000787::MIMAT0003652::MIMAT0013085::MIMAT0019387::MIMAT0021817::MIMAT0022532::MIMAT0023817::MIMAT0026246 | aca-miR-18a(18)::hsa-miR-18a(17)::mmu-miR-18a(17)::pma-miR-18a(18)::rno-miR-18a(18)::xtr-miR-18a(18) | UAAGGUGCAUCUAGUGCAGAUAG |
| 002423 | hsa-miR-18a# | cgr-miR-18a-3p::hsa-miR-18a-3p | MIMAT0002891::MIMAT0023818 | hsa-miR-18a*(17) | ACUGCCCUAAGUGCUCCUUCUGG |
| 002424 | hsa-miR-19a# | hsa-miR-19a-5p | MIMAT0004490 | hsa-miR-19a*(17) | AGUUUUGCAUAGUUGCACUACA |
| 002425 | hsa-miR-19b-1# | cgr-miR-19b-5p::hsa-miR-19b-1-5p::mmu-miR-19b-1-5p | MIMAT0004491::MIMAT0017065::MIMAT0023840 | hsa-miR-19b-1*(17)::mmu-miR-19b-1*(17) | AGUUUUGCAGGUUUGCAUCCAGC |
| 002427 | hsa-miR-499-3p | eca-miR-499-3p::hsa-miR-499a-3p::mml-miR-499-3p::ppy-miR-499-3p::ptr-miR-499::rno-miR-499-3p::ssc-miR-499-3p | MIMAT0004772::MIMAT0006362::MIMAT0008172::MIMAT0013109::MIMAT0015921::MIMAT0017227::MIMAT0017373 | hsa-miR-499-3p(17)::rno-miR-499*(18) | AACAUCACAGCAAGUCUGUGCU |
| 002428 | hsa-miR-500 | bta-miR-500::eca-miR-500::hsa-miR-500a-5p::mml-miR-500::ppy-miR-500::ptr-miR-500 | MIMAT0004773::MIMAT0006363::MIMAT0008173::MIMAT0009337::MIMAT0013223::MIMAT0015922 | hsa-miR-500(15)::hsa-miR-500a(17) | UAAUCCUUGCUACCUGGGUGAGA |
| 002429 | hsa-miR-548c-5p | hsa-miR-548am-5p::hsa-miR-548c-5p::hsa-miR-548o-5p::ptr-miR-548o | MIMAT0004806::MIMAT0022738::MIMAT0022740::MIMAT0024054 | | AAAAGUAAUUGCGGUUUUUGCC |
| 002430 | hsa-miR-624 | hsa-miR-624-3p::ppy-miR-624::ptr-miR-624 | MIMAT0004807::MIMAT0008284::MIMAT0016065 | hsa-miR-624(17) | CACAAGGUAUUGGUAUUACCU |
| 002431 | hsa-miR-625 | hsa-miR-625-5p | MIMAT0003294 | hsa-miR-625(17) | AGGGGGAAAGUUCUAUAGUCC |
| 002432 | hsa-miR-625# | hsa-miR-625-3p | MIMAT0004808 | hsa-miR-625*(17) | GACUAUAGAACUUUCCCCCUCA |
| 002433 | hsa-miR-628-5p | bta-miR-628::cfa-miR-628::cgr-miR-628::eca-miR-628a::ggo-miR-628::hsa-miR-628-5p::mml-miR-628-5p::ppy-miR-628-5p::ssc-miR-628 | MIMAT0004809::MIMAT0006474::MIMAT0009356::MIMAT0009921::MIMAT0012903::MIMAT0013953::MIMAT0016067::MIMAT0023989::MIMAT0024185 | | AUGCUGACAUAUUUACUAGAGG |
| 002434 | hsa-miR-628-3p | hsa-miR-628-3p::mml-miR-628-3p::ppy-miR-628-3p::ptr-miR-628 | MIMAT0003297::MIMAT0006475::MIMAT0008287::MIMAT0016068 | | UCUAGUAAGAGUGGCAGUCGA |
| 002435 | hsa-miR-501-3p | hsa-miR-501-3p::ptr-miR-501 | MIMAT0004774::MIMAT0008174 | | AAUGCACCCGGGCAAGGAUUCU |
| 002436 | hsa-miR-629 | hsa-miR-629-5p | MIMAT0004810 | hsa-miR-629(17) | UGGGUUUACGUUGGGAGAACU |
| 002437 | hsa-miR-20a# | hsa-miR-20a-3p | MIMAT0004493 | hsa-miR-20a*(17) | ACUGCAUUAUGAGCACUUAAAG |
| 002438 | hsa-miR-21# | hsa-miR-21-3p | MIMAT0004494 | hsa-miR-21*(17) | CAACACCAGUCGAUGGGCUGU |
| 002439 | hsa-miR-23a# | cgr-miR-23a-5p::hsa-miR-23a-5p::mmu-miR-23a-5p::rno-miR-23a-5p | MIMAT0004496::MIMAT0004712::MIMAT0017019::MIMAT0023862 | hsa-miR-23a*(17)::mmu-miR-23a*(17)::rno-miR-23a*(18) | GGGGUUCCUGGGGAUGGGAUUU |
| 002440 | hsa-miR-24-1# | hsa-miR-24-1-5p | MIMAT0000079 | hsa-miR-24-1*(17) | UGCCUACUGAGCUGAUAUCAGU |
| 002441 | hsa-miR-24-2# | hsa-miR-24-2-5p | MIMAT0004497 | hsa-miR-24-2*(17) | UGCCUACUGAGCUGAAACACAG |
| 002442 | hsa-miR-25# | hsa-miR-25-5p | MIMAT0004498 | hsa-miR-25*(17) | AGGCGGAGACUUGGGCAAUUG |
| 002443 | hsa-miR-26a-1# | hsa-miR-26a-1-3p::mmu-miR-26a-1-3p | MIMAT0004499::MIMAT0017020 | hsa-miR-26a-1*(17)::mmu-miR-26a-1*(17) | CCUAUUCUUGGUUACUUGCACG |
| 002444 | hsa-miR-26b# | cgr-miR-26b-3p::hsa-miR-26b-3p::mmu-miR-26b-3p::rno-miR-26b-3p | MIMAT0004500::MIMAT0004630::MIMAT0004714::MIMAT0023873 | hsa-miR-26b*(17)::mmu-miR-26b*(17)::rno-miR-26b*(18) | CCUGUUCUCCAUUACUUGGCUC |
| 002445 | hsa-miR-27a# | bta-miR-27a-5p::cgr-miR-27a-5p::hsa-miR-27a-5p::mmu-miR-27a-5p::rno-miR-27a-5p | MIMAT0004501::MIMAT0004633::MIMAT0004715::MIMAT0012532::MIMAT0023874 | hsa-miR-27a*(17)::mmu-miR-27a*(17)::rno-miR-27a*(18) | AGGGCUUAGCUGCUUGUGAGCA |
| 002446 | hsa-miR-28-3p | cfa-miR-28::cgr-miR-28-3p::eca-miR-28-3p::hsa-miR-28-3p::rno-miR-28-3p::ssc-miR-28-3p | MIMAT0004502::MIMAT0004716::MIMAT0006675::MIMAT0013093::MIMAT0015211::MIMAT0023879 | rno-miR-28*(18) | CACUAGAUUGUGAGCUCCUGGA |
| 002447 | hsa-miR-29a# | hsa-miR-29a-5p::mmu-miR-29a-5p::oan-miR-29a-1-5p::rno-miR-29a-5p | MIMAT0004503::MIMAT0004631::MIMAT0004718::MIMAT0006806 | hsa-miR-29a*(17)::mmu-miR-29a*(17)::oan-miR-29a-1*(18)::rno-miR-29a*(18) | ACUGAUUUCUUUUGGUGUUCAG |
| 002619 | hsa-let-7b | bta-let-7b::ccr-let-7b::cfa-let-7b::cgr-let-7b::dre-let-7b::fru-let-7b::gga-let-7b::hsa-let-7b-5p::mdo-let-7b::mml-let-7b::mmu-let-7b-5p::oan-let-7b-5p::pol-let-7b-5p::ppy-let-7b::ptr-let-7b::rno-let-7b-5p::tgu-let-7b::tni-let-7b | MIMAT0000063::MIMAT0000522::MIMAT0000775::MIMAT0001102::MIMAT0001760::MIMAT0003016::MIMAT0003017::MIMAT0004162::MIMAT0004331::MIMAT0006152::MIMAT0006897::MIMAT0007937::MIMAT0009836::MIMAT0014509::MIMAT0015722::MIMAT0023717::MIMAT0025414::MIMAT0026190 | hsa-let-7b(17)::mmu-let-7b(17)::oan-let-7b(18)::rno-let-7b(18) | UGAGGUAGUAGGUUGUGUGGUU |
| 002623 | hsa-miR-155 | bta-miR-155::cfa-miR-155::eca-miR-155::hsa-miR-155-5p::mml-miR-155::oan-miR-155-5p::ppy-miR-155::ptr-miR-155 | MIMAT0000646::MIMAT0006216::MIMAT0006671::MIMAT0006988::MIMAT0008048::MIMAT0009241::MIMAT0013182::MIMAT0015777 | hsa-miR-155(17)::oan-miR-155(18) | UUAAUGCUAAUCGUGAUAGGGGU |
| 002642 | hsa-miR-151-5P | bta-miR-151-5p::cfa-miR-151::cgr-miR-151-5p::eca-miR-151-5p::hsa-miR-151a-5p::mml-miR-151-5p::mmu-miR-151-5p::ppy-miR-151a-5p::rno-miR-151-5p::ssc-miR-151-5p | MIMAT0000613::MIMAT0003523::MIMAT0004536::MIMAT0004697::MIMAT0006212::MIMAT0006615::MIMAT0013004::MIMAT0013882::MIMAT0015775::MIMAT0023782 | bta-miR-151*(18)::hsa-miR-151-5p(17)::ppy-miR-151-5p(18)::rno-miR-151(18) | UCGAGGAGCUCACAGUCUAGU |
| 002643 | hsa-miR-765 | hsa-miR-765::ppy-miR-765::ptr-miR-765 | MIMAT0003945::MIMAT0008320::MIMAT0016105 | | UGGAGGAGAAGGAAGGUGAUG |
| 002658 | hsa-miR-338-5P | aca-miR-338-5p::eca-miR-338-5p::hsa-miR-338-5p::mml-miR-338-5p::mmu-miR-338-5p::ppy-miR-338-5p::rno-miR-338-5p | MIMAT0004646::MIMAT0004647::MIMAT0004701::MIMAT0006277::MIMAT0013035::MIMAT0015840::MIMAT0021937 | rno-miR-338*(18) | AACAAUAUCCUGGUGCUGAGUG |
| 002672 | hsa-miR-620 | hsa-miR-620 | MIMAT0003289 |  | AUGGAGAUAGAUAUAGAAAU |
| 002675 | hsa-miR-577 | hsa-miR-577::mml-miR-577::ppy-miR-577::ptr-miR-577 | MIMAT0003242::MIMAT0006438::MIMAT0008251::MIMAT0016019 | | UAGAUAAAAUAUUGGUACCUG |
| 002676 | hsa-miR-144 | aca-miR-144-3p::dre-miR-144::eca-miR-144::fru-miR-144::hsa-miR-144-3p::mml-miR-144::mmu-miR-144-3p::oan-miR-144-3p::rno-miR-144-3p::tgu-miR-144-3p::tni-miR-144 | MIMAT0000156::MIMAT0000436::MIMAT0000850::MIMAT0001841::MIMAT0003020::MIMAT0003021::MIMAT0006202::MIMAT0006903::MIMAT0013024::MIMAT0014596::MIMAT0021772 | aca-miR-144(18)::hsa-miR-144(17)::mmu-miR-144(17)::oan-miR-144(18)::rno-miR-144(18)::tgu-miR-144(18) | UACAGUAUAGAUGAUGUACU |
| 002677 | hsa-miR-590-3P | cfa-miR-590::eca-miR-590-3p::hsa-miR-590-3p::mmu-miR-590-3p::ppy-miR-590-3p::ptr-miR-590 | MIMAT0004801::MIMAT0004896::MIMAT0006700::MIMAT0008261::MIMAT0013059::MIMAT0016033 | | UAAUUUUAUGUAUAAGCUAGU |
| 002678 | hsa-miR-191# | hsa-miR-191-3p | MIMAT0001618 | hsa-miR-191*(17) | GCUGCGCUUGGAUUUCGUCCCC |
| 002681 | hsa-miR-665 | hsa-miR-665::mml-miR-665::ptr-miR-665 | MIMAT0004952::MIMAT0008312::MIMAT0012786 | | ACCAGGAGGCUGAGGCCCCU |
| 002743 | hsa-miR-520D-3P | hsa-miR-520d-3p::ppy-miR-520d-3p::ptr-miR-520d | MIMAT0002856::MIMAT0008200::MIMAT0015972 | | AAAGUGCUUCUCUUUGGUGGGU |
| 002752 | hsa-miR-1224-3P | hsa-miR-1224-3p::mml-miR-1224-3p::ppy-miR-1224-3p::ptr-miR-1224-3p | MIMAT0005459::MIMAT0005569::MIMAT0005571::MIMAT0016159 | mml-miR-1224*(18) | CCCCACCUCCUCUCUCCUCAG |
| 002756 | hsa-miR-513C | hsa-miR-513c-5p | MIMAT0005789 | hsa-miR-513c(17) | UUCUCAAGGAGGUGUCGUUUAU |
| 002757 | hsa-miR-513B | hsa-miR-513b::mml-miR-513b::pbi-miR-513b::ppy-miR-513b::ssy-miR-513b | MIMAT0005759::MIMAT0005767::MIMAT0005785::MIMAT0005788::MIMAT0015940 | | UUCACAAGGAGGUGUCAUUUAU |
| 002758 | hsa-miR-1226# | hsa-miR-1226-5p | MIMAT0005576 | hsa-miR-1226*(17) | GUGAGGGCAUGCAGGCCUGGAUGGGG |
| 002761 | hsa-miR-1236 | hsa-miR-1236-3p::ppy-miR-1236 | MIMAT0005591::MIMAT0016166 | hsa-miR-1236(18) | CCUCUUCCCCUUGUCUCUCCAG |
| 002763 | hsa-miR-1228# | hsa-miR-1228-5p | MIMAT0005582 | hsa-miR-1228*(17) | GUGGGCGGGGGCAGGUGUGUG |
| 002766 | hsa-miR-1225-3P | hsa-miR-1225-3p::ppy-miR-1225-3p | MIMAT0005573::MIMAT0016161 | | UGAGCCCCUGUGCCGCCCCCAG |
| 002768 | hsa-miR-1233 | hsa-miR-1233-3p::mml-miR-1233::ptr-miR-1233 | MIMAT0005588::MIMAT0007966::MIMAT0012789 | hsa-miR-1233(18) | UGAGCCCUGUCCUCCCGCAG |
| 002769 | hsa-miR-1227 | hsa-miR-1227-3p | MIMAT0005580 | hsa-miR-1227(18) | CGUGCCACCCUUUUCCCCAG |
| 002773 | hsa-miR-1286 | ggo-miR-1286::hsa-miR-1286::ppy-miR-1286::ptr-miR-1286 | MIMAT0005877::MIMAT0008005::MIMAT0016209::MIMAT0024291 | | UGCAGGACCAAGAUGAGCCCU |
| 002775 | hsa-miR-548M | hsa-miR-548m | MIMAT0005917 |  | CAAAGGUAUUUGUGGUUUUUG |
| 002776 | hsa-miR-1179 | bta-miR-1179::eca-miR-1179::ggo-miR-1179::hsa-miR-1179::mml-miR-1179::ptr-miR-1179 | MIMAT0005824::MIMAT0007948::MIMAT0009957::MIMAT0012887::MIMAT0024218::MIMAT0024329 | | AAGCAUUCUUUCAUUGGUUGG |
| 002777 | hsa-miR-1178 | hsa-miR-1178-3p::ppy-miR-1178::ptr-miR-1178 | MIMAT0005823::MIMAT0007947::MIMAT0016143 | hsa-miR-1178(18) | UUGCUCACUGUUCUUCCCUAG |
| 002778 | hsa-miR-1205 | hsa-miR-1205::ppy-miR-1205::ptr-miR-1205 | MIMAT0005869::MIMAT0007959::MIMAT0016154 | | UCUGCAGGGUUUGCUUUGAG |
| 002779 | hsa-miR-1271 | ggo-miR-1271::hsa-miR-1271-5p::mml-miR-1271::ppy-miR-1271::ptr-miR-1271 | MIMAT0005796::MIMAT0007992::MIMAT0016195::MIMAT0024192::MIMAT0024315 | hsa-miR-1271(17) | CUUGGCACCUAGCAAGCACUCA |
| 002781 | hsa-miR-1201 |  |  | hsa-miR-1201(15)::ppy-miR-1201(15)::ptr-miR-1201(15) | AGCCUGAUUAAACACAUGCUCUGA |
| 002783 | hsa-miR-548J | hsa-miR-548j | MIMAT0005875 |  | AAAAGUAAUUGCGGUCUUUGGU |
| 002784 | hsa-miR-1263 | hsa-miR-1263::ppy-miR-1263::ptr-miR-1263 | MIMAT0005915::MIMAT0007987::MIMAT0016187 | | AUGGUACCCUGGCAUACUGAGU |
| 002785 | hsa-miR-1294 | hsa-miR-1294::ptr-miR-1294 | MIMAT0005884::MIMAT0008013 | | UGUGAGGUUGGCAUUGUUGUCU |
| 002789 | hsa-miR-1269 | hsa-miR-1269a | MIMAT0005923 | hsa-miR-1269(17) | CUGGACUGAGCCGUGCUACUGG |
| 002790 | hsa-miR-1265 | hsa-miR-1265::ptr-miR-1265 | MIMAT0005918::MIMAT0007989 | | CAGGAUGUGGUCAAGUGUUGUU |
| 002791 | hsa-miR-1244 | hsa-miR-1244::ppy-miR-1244::ptr-miR-1244 | MIMAT0005896::MIMAT0007970::MIMAT0016169 | | AAGUAGUUGGUUUGUAUGAGAUGGUU |
| 002792 | hsa-miR-1303 | hsa-miR-1303::ptr-miR-1303 | MIMAT0005891::MIMAT0008022 | | UUUAGAGACGGGGUCUUGCUCU |
| 002796 | hsa-miR-1259 |  |  | hsa-miR-1259(15)::ptr-miR-1259(15) | AUAUAUGAUGACUUAGCUUUU |
| 002798 | hsa-miR-548P | hsa-miR-548p::ptr-miR-548p | MIMAT0005934::MIMAT0008228 | | UAGCAAAAACUGCAGUUACUUU |
| 002799 | hsa-miR-1264 | eca-miR-1264::hsa-miR-1264::ppy-miR-1264::ptr-miR-1264 | MIMAT0005791::MIMAT0007988::MIMAT0013195::MIMAT0016188 | | CAAGUCUUAUUUGAGCACCUGUU |
| 002801 | hsa-miR-1255B | hsa-miR-1255b-5p::ptr-miR-1255b | MIMAT0005945::MIMAT0007980 | hsa-miR-1255b(17) | CGGAUGAGCAAAGAAAGUGGUU |
| 002803 | hsa-miR-1282 | bta-miR-1282::eca-miR-1282::hsa-miR-1282::ppy-miR-1282::ptr-miR-1282 | MIMAT0005940::MIMAT0008001::MIMAT0009954::MIMAT0012889::MIMAT0016203 | | UCGUUUGCCUUUUUCUGCUU |
| 002805 | hsa-miR-1255A | hsa-miR-1255a | MIMAT0005906 |  | AGGAUGAGCAAAGAAAGUAGAUU |
| 002807 | hsa-miR-1270 | hsa-miR-1270 | MIMAT0005924 |  | CUGGAGAUAUGGAAGAGCUGUGU |
| 002810 | hsa-miR-1197 | bta-miR-1197::eca-miR-1197::hsa-miR-1197::mmu-miR-1197-3p::oar-miR-1197-3p::ppy-miR-1197::ptr-miR-1197 | MIMAT0005858::MIMAT0005955::MIMAT0007954::MIMAT0009966::MIMAT0013125::MIMAT0016149::MIMAT0019258 | mmu-miR-1197(17) | UAGGACACAUGGUCUACUUCU |
| 002815 | hsa-miR-1324 | hsa-miR-1324::ptr-miR-1324 | MIMAT0005956::MIMAT0008029 | | CCAGACAGAAUUCUAUGCACUUUC |
| 002816 | hsa-miR-548H | hsa-miR-548h-5p::ptr-miR-548h | MIMAT0005928::MIMAT0008222 | hsa-miR-548h(17) | AAAAGUAAUCGCGGUUUUUGUC |
| 002818 | hsa-miR-1254 | hsa-miR-1254::ptr-miR-1254 | MIMAT0005905::MIMAT0007979 | | AGCCUGGAAGCUGGAGCCUGCAGU |
| 002819 | hsa-miR-548K | hsa-miR-548k::ptr-miR-548k | MIMAT0005882::MIMAT0008225 | | AAAAGUACUUGCGGAUUUUGCU |
| 002820 | hsa-miR-1251 | bta-miR-1251::hsa-miR-1251::mmu-miR-1251-5p::ppy-miR-1251::ptr-miR-1251 | MIMAT0005903::MIMAT0007977::MIMAT0009963::MIMAT0014824::MIMAT0016176 | mmu-miR-1251(17) | ACUCUAGCUGCCAAAGGCGCU |
| 002822 | hsa-miR-1285 | hsa-miR-1285-3p::ppy-miR-1285b::ptr-miR-1285 | MIMAT0005876::MIMAT0008004::MIMAT0016208 | hsa-miR-1285(17) | UCUGGGCAACAAAGUGAGACCU |
| 002823 | hsa-miR-1245 | hsa-miR-1245a::ptr-miR-1245 | MIMAT0005897::MIMAT0007971 | hsa-miR-1245(17) | AAGUGAUCUAAAGGCCUACAU |
| 002824 | hsa-miR-1292 | hsa-miR-1292-5p::ptr-miR-1292 | MIMAT0005943::MIMAT0008011 | hsa-miR-1292(18) | UGGGAACGGGUUCCGGCAGACGCUG |
| 002827 | hsa-miR-1301 | hsa-miR-1301::ppy-miR-1301 | MIMAT0005797::MIMAT0016221 | | UUGCAGCUGCCUGGGAGUGACUUC |
| 002829 | hsa-miR-1200 | hsa-miR-1200::ppy-miR-1200 | MIMAT0005863::MIMAT0016150 | | CUCCUGAGCCAUUCUGAGCCUC |
| 002830 | hsa-miR-1182 | hsa-miR-1182::ptr-miR-1182 | MIMAT0005827::MIMAT0007950 | | GAGGGUCUUGGGAGGGAUGUGAC |
| 002832 | hsa-miR-1288 | hsa-miR-1288::ptr-miR-1288 | MIMAT0005942::MIMAT0008006 | | UGGACUGCCCUGAUCUGGAGA |
| 002838 | hsa-miR-1291 | eca-miR-1291a::hsa-miR-1291::ppy-miR-1291::ptr-miR-1291 | MIMAT0005881::MIMAT0008009::MIMAT0012968::MIMAT0016213 | | UGGCCCUGACUGAAGACCAGCAGU |
| 002840 | hsa-miR-1275 | hsa-miR-1275::ppy-miR-1275::ptr-miR-1275 | MIMAT0005929::MIMAT0007996::MIMAT0016199 | | GUGGGGGAGAGGCUGUC |
| 002841 | hsa-miR-1183 | hsa-miR-1183::ppy-miR-1183::ptr-miR-1183 | MIMAT0005828::MIMAT0007951::MIMAT0016147 | | CACUGUAGGUGAUGGUGAGAGUGGGCA |
| 002842 | hsa-miR-1184 | hsa-miR-1184::ptr-miR-1184 | MIMAT0005829::MIMAT0007952 | | CCUGCAGCGACUUGAUGGCUUCC |
| 002843 | hsa-miR-1276 | hsa-miR-1276::ptr-miR-1276 | MIMAT0005930::MIMAT0007997 | | UAAAGAGCCCUGUGGAGACA |
| 002844 | hsa-miR-320B | hsa-miR-320b::mml-miR-320b::ppy-miR-320b::ptr-miR-320b | MIMAT0005792::MIMAT0008094::MIMAT0015822::MIMAT0024312 | | AAAAGCUGGGUUGAGAGGGCAA |
| 002845 | hsa-miR-1272 | hsa-miR-1272::ptr-miR-1272 | MIMAT0005925::MIMAT0007993 | | GAUGAUGAUGGCAGCAAAUUCUGAAA |
| 002847 | hsa-miR-1180 | hsa-miR-1180::ppy-miR-1180 | MIMAT0005825::MIMAT0016145 | | UUUCCGGCUCGCGUGGGUGUGU |
| 002850 | hsa-miR-1256 | hsa-miR-1256::ppy-miR-1256::ptr-miR-1256 | MIMAT0005907::MIMAT0007981::MIMAT0016182 | | AGGCAUUGACUUCUCACUAGCU |
| 002851 | hsa-miR-1278 | hsa-miR-1278 | MIMAT0005936 |  | UAGUACUGUGCAUAUCAUCUAU |
| 002852 | hsa-miR-1262 | hsa-miR-1262::ppy-miR-1262::ptr-miR-1262 | MIMAT0005914::MIMAT0007986::MIMAT0016186 | | AUGGGUGAAUUUGUAGAAGGAU |
| 002854 | hsa-miR-1243 | hsa-miR-1243 | MIMAT0005894 |  | AACUGGAUCAAUUAUAGGAGUG |
| 002857 | hsa-miR-663B | hsa-miR-663b::ppy-miR-663b::ptr-miR-663b | MIMAT0005867::MIMAT0008310::MIMAT0016093 | | GGUGGCCCGGCCGUGCCUGAGG |
| 002860 | hsa-miR-1252 | hsa-miR-1252 | MIMAT0005944 |  | AGAAGGAAAUUGAAUUCAUUUA |
| 002861 | hsa-miR-1298 | bta-miR-1298::eca-miR-1298::hsa-miR-1298::mml-miR-1298::mmu-miR-1298-5p::ppy-miR-1298::ptr-miR-1298::rno-miR-1298 | MIMAT0005800::MIMAT0008017::MIMAT0009970::MIMAT0013196::MIMAT0014809::MIMAT0016219::MIMAT0024316::MIMAT0025060 | mmu-miR-1298(17) | UUCAUUCGGCUGUCCAGAUGUA |
| 002863 | hsa-miR-1290 | hsa-miR-1290::ptr-miR-1290 | MIMAT0005880::MIMAT0008008 | | UGGAUUUUUGGAUCAGGGA |
| 002867 | hsa-miR-1305 | hsa-miR-1305 | MIMAT0005893 |  | UUUUCAACUCUAAUGGGAGAGA |
| 002868 | hsa-miR-1249 | bta-miR-1249::ggo-miR-1249::hsa-miR-1249::mml-miR-1249::mmu-miR-1249-3p::ppy-miR-1249::ptr-miR-1249::rno-miR-1249::ssc-miR-1249 | MIMAT0005901::MIMAT0007975::MIMAT0009976::MIMAT0010560::MIMAT0016174::MIMAT0017892::MIMAT0024247::MIMAT0024324::MIMAT0025385 | mmu-miR-1249(17) | ACGCCCUUCCCCCCCUUCUUCA |
| 002870 | hsa-miR-1248 | bta-miR-1248::hsa-miR-1248::ppy-miR-1248::ptr-miR-1248 | MIMAT0005900::MIMAT0007974::MIMAT0009972::MIMAT0016173 | | ACCUUCUUGUAUAAGCACUGUGCUAAA |
| 002871 | hsa-miR-1289 | eca-miR-1289::hsa-miR-1289::ppy-miR-1289::ptr-miR-1289 | MIMAT0005879::MIMAT0008007::MIMAT0013077::MIMAT0016211 | | UGGAGUCCAGGAAUCUGCAUUUU |
| 002872 | hsa-miR-1204 | hsa-miR-1204::ppy-miR-1204::ptr-miR-1204 | MIMAT0005868::MIMAT0007958::MIMAT0016153 | | UCGUGGCCUGGUCUCCAUUAU |
| 002873 | hsa-miR-1826 |  |  | hsa-miR-1826(15) | AUUGAUCAUCGACACUUCGAACGCAAU |
| 002874 | hsa-miR-1304 | hsa-miR-1304-5p | MIMAT0005892 | hsa-miR-1304(17) | UUUGAGGCUACAGUGAGAUGUG |
| 002877 | hsa-miR-1203 | hsa-miR-1203::ptr-miR-1203 | MIMAT0005866::MIMAT0007957 | | CCCGGAGCCAGGAUGCAGCUC |
| 002878 | hsa-miR-1206 | hsa-miR-1206::ptr-miR-1206 | MIMAT0005870::MIMAT0007960 | | UGUUCAUGUAGAUGUUUAAGC |
| 002879 | hsa-miR-548G | hsa-miR-548g-3p | MIMAT0005912 | hsa-miR-548g(17) | AAAACUGUAAUUACUUUUGUAC |
| 002880 | hsa-miR-1208 | hsa-miR-1208::ppy-miR-1208::ptr-miR-1208 | MIMAT0005873::MIMAT0007962::MIMAT0016157 | | UCACUGUUCAGACAGGCGGA |
| 002881 | hsa-miR-548E | hsa-miR-548e | MIMAT0005874 |  | AAAAACUGAGACUACUUUUGCA |
| 002883 | hsa-miR-1274A |  |  | hsa-miR-1274a(16) | GUCCCUGUUCAGGCGCCA |
| 002884 | hsa-miR-1274B |  |  | hsa-miR-1274b(16)::ptr-miR-1274b(16) | UCCCUGUUCGGGCGCCA |
| 002885 | hsa-miR-1267 | hsa-miR-1267::ptr-miR-1267 | MIMAT0005921::MIMAT0007991 | | CCUGUUGAAGUGUAAUCCCCA |
| 002887 | hsa-miR-1250 | hsa-miR-1250::ppy-miR-1250::ptr-miR-1250 | MIMAT0005902::MIMAT0007976::MIMAT0016175 | | ACGGUGCUGGAUGUGGCCUUU |
| 002888 | hsa-miR-548N | hsa-miR-548n::ptr-miR-548n | MIMAT0005916::MIMAT0008227 | | CAAAAGUAAUUGUGGAUUUUGU |
| 002890 | hsa-miR-1283 | hsa-miR-1283::ppy-miR-1283a::ptr-miR-1283 | MIMAT0005799::MIMAT0008002::MIMAT0016204 | | UCUACAAAGGAAAGCGCUUUCU |
| 002893 | hsa-miR-1247 | hsa-miR-1247-5p::mmu-miR-1247-5p::ppy-miR-1247::ptr-miR-1247 | MIMAT0005899::MIMAT0007973::MIMAT0014800::MIMAT0016172 | hsa-miR-1247(17)::mmu-miR-1247(17) | ACCCGUCCCGUUCGUCCCCGGA |
| 002894 | hsa-miR-1253 | hsa-miR-1253::ptr-miR-1253 | MIMAT0005904::MIMAT0007978 | | AGAGAAGAAGAUCAGCCUGCA |
| 002895 | hsa-miR-720 |  |  | hsa-miR-720(18)::ptr-miR-720(18) | UCUCGCUGGGGCCUCCA |
| 002896 | hsa-miR-1260 | hsa-miR-1260a::ppy-miR-1260a | MIMAT0005911::MIMAT0016184 | hsa-miR-1260(17)::ppy-miR-1260(18) | AUCCCACCUCUGCCACCA |
| 002897 | hsa-miR-664 | hsa-miR-664a-3p::ppy-miR-664::ptr-miR-664a | MIMAT0005949::MIMAT0008311::MIMAT0016094 | hsa-miR-664(17)::hsa-miR-664-3p(18)::ptr-miR-664(18) | UAUUCAUUUAUCCCCAGCCUACA |
| 002901 | hsa-miR-1302 | eca-miR-1302d::hsa-miR-1302::ppy-miR-1302::ptr-miR-1302 | MIMAT0005890::MIMAT0008021::MIMAT0012969::MIMAT0016222 | | UUGGGACAUACUUAUGCUAAA |
| 002902 | hsa-miR-1300 |  |  | hsa-miR-1300(13)::ptr-miR-1300a(13) | UUGAGAAGGAGGCUGCUG |
| 002903 | hsa-miR-1284 | hsa-miR-1284::ppy-miR-1284::ptr-miR-1284 | MIMAT0005941::MIMAT0008003::MIMAT0016206 | | UCUAUACAGACCCUGGCUUUUC |
| 002904 | hsa-miR-548L | hsa-miR-548l::ptr-miR-548l | MIMAT0005889::MIMAT0008226 | | AAAAGUAUUUGCGGGUUUUGUC |
| 002905 | hsa-miR-1293 | hsa-miR-1293::ptr-miR-1293 | MIMAT0005883::MIMAT0008012 | | UGGGUGGUCUGGAGAUUUGUGC |
| 002907 | hsa-miR-1825 | hsa-miR-1825::ptr-miR-1825 | MIMAT0006765::MIMAT0008051 | | UCCAGUGCCCUCCUCUCC |
| 002908 | hsa-miR-1296 | bta-miR-1296::eca-miR-1296::ggo-miR-1296::hsa-miR-1296::ppy-miR-1296::ptr-miR-1296::ssc-miR-1296-5p | MIMAT0005794::MIMAT0008015::MIMAT0009964::MIMAT0012890::MIMAT0016217::MIMAT0022964::MIMAT0024172 | | UUAGGGCCCUGGCUCCAUCUCC |
| 002909 | hsa-miR-548I | hsa-miR-548i::ptr-miR-548i | MIMAT0005935::MIMAT0008223 | | AAAAGUAAUUGCGGAUUUUGCC |
| 002910 | hsa-miR-1257 | hsa-miR-1257::ppy-miR-1257 | MIMAT0005908::MIMAT0016183 | | AGUGAAUGAUGGGUUCUGACC |
| 002927 | hsa-miR-1238 | hsa-miR-1238-3p | MIMAT0005593 | hsa-miR-1238(18) | CUUCCUCGUCUGUCUGCCCC |
